# Linking by‐caught cetacean traits to fishing techniques: Insights from two species of small cetaceans

**DOI:** 10.1002/eap.70216

**Published:** 2026-03-26

**Authors:** Mathieu Brevet, Matthieu Authier, Hélène Peltier, Laurent Dubroca

**Affiliations:** ^1^ Ifremer, HMMN Laboratoire Ressources Halieutiques Port‐en‐Bessin France; ^2^ Observatoire Pelagis, UAR 3462, CNRS‐La Rochelle Université La Rochelle France; ^3^ Present address: UPPA ‐ Université de Pau et des Pays de l'Adour, Avenue de l'Université, BP 576‐64012, Pau Cedex France

**Keywords:** by‐catch risk, cetacean conservation, *Delphinus delphis*, fishing behaviors, incidental by‐catch, phenotype sensitivity, phenotype vulnerability, *Phocoena phocoena*, spatiotemporal management

## Abstract

By‐catch is one of the main threats currently looming over small cetaceans worldwide. Improving knowledge of the interactions between fishing activities and small cetaceans is paramount to design cost‐effective mitigation measures. In particular, not all individuals within a population may be exposed to the same by‐catch risk: in dolphins, juveniles and males appear to be more sensitive to by‐catch. Yet, few studies have investigated how individual‐level characteristics (such as age, sex, body size) correlate with fishing practices in these species. Using French by‐catch observations, declarations, and stranding databases on two small cetaceans (*Delphinus delphis* and *Phocoena phocoena*), we explored phenotypic vulnerability to by‐catch by correlating the phenotypes of by‐caught individuals to fishing‐operation characteristics (including fishing gear, mesh size, the presence of an acoustic deterrent, targeted and fished taxa, and fishing effort). This investigation allowed us to outline by‐catch sensitivity and vulnerability profiles. Again, we found that males and young individuals were more sensitive to by‐catch, with spatiotemporal sensitivity patterns. Smaller individuals appeared to be caught on the northern French coast and in spring, and more males were caught on the southern French coast. We then found larger body‐sized dolphins to be more vulnerable to trawls compared to gillnets. For the latter fisheries, the size and body mass of by‐caught harbor porpoises were positively correlated with mesh size. Targeting soles or hakes was also associated with a larger body size of by‐caught dolphins compared with targeting Sparidae or sea bass. Finally, we found larger individuals to be by‐caught in the presence of an acoustic deterrent device. Our results suggest age‐specific by‐catch sensitivity and vulnerability to fishing techniques, which may be due to biological factors such as social behavior and diet. Our study therefore advocates for a better consideration of spatiotemporal patterns in individuals' sensitivity to by‐catch and age‐ or sex‐specific vulnerabilities to particular fishing activity profiles.

## INTRODUCTION

By‐catch, the unintentional capture or killing of nontarget species in commercial or recreational fisheries, may lead to population declines (Lewison et al., 2004; Soykan et al., 2008). To prevent such declines, it is necessary to implement management measures (Komoroske & Lewison, 2015; Lewison et al., 2004; Read, 2008). In particular, it is key to assess the impact of by‐catch and seek mitigation measures, including technological devices such as acoustic deterrents for marine mammals (e.g., Puente et al., 2023). Recent research has made significant progress in quantifying risks (Hines et al., 2020; Mannocci et al., 2021; Zhou et al., 2019) by (i) identifying which practices are the most conducive to by‐catch (Clay et al., 2019; Gilman et al., 2016; Peltier et al., 2020, 2021); by (ii) estimating rates more accurately (Carretta, 2021; Kindt‐Larsen et al., 2023; Rouby et al., 2022); and by (iii) better understanding exposure within a population (Byrd & Hohn, 2017; Gianuca et al., 2017; Heswall et al., 2021). Specifically, in small cetaceans, by‐catch phenotypic sensitivity (i.e., likelihood of by‐catch as a function of individuals' traits) is often related to sex or age. In common dolphins (*Delphinus delphis*), males are often more prone to by‐catch (Fernández‐Contreras et al., 2010; López et al., 2002; McGovern et al., 2018; Westgate & Read, 2007; but see Meager & Sumpton, 2016 in another area), especially young males (Brown et al., 2014). In bottlenose dolphins (*Tursiops truncatus*), this is the case for juveniles (Byrd & Hohn, 2017), and again especially young males (Fruet et al., 2012). High by‐catch mortality is also frequently reported in mature females (Brown et al., 2014; Murphy & Rogan, 2006 in common dolphins, Fruet et al., 2012 in bottlenose dolphins, Marçalo et al., 2021 in striped dolphins *Stenella coeruleoalba*, Vishnyakova & Gol'din, 2015 in harbor porpoises *Phocoena phocoena*), with potentially important impacts on population dynamics (Moore & Read, 2008).

Fishing techniques modulate by‐catch risk (Brown et al., 2013; Northridge et al., 2017; Moyes et al., 2025). The importance of assessing the trait‐specific (as age, sex, size) impacts of fishing techniques on by‐catch risk, hereafter referred to as the phenotypic vulnerability to by‐catch, and their effects on population dynamics has long been recognized. For example, Hall (1996) predicted that some fishing techniques could affect more adult individuals than juveniles and consequently impact population growth. However, to our knowledge, no recent study has examined how individual traits of by‐caught individuals might be related to fishing techniques (but see Wallace et al., 2008 for turtles or Jones et al., 2010 for elasmobranchs). There is a particular lack of knowledge for small cetaceans (but see De Boer et al., 2012 for bottlenose dolphins).

Previous studies on traits of by‐caught individuals have described various mechanisms linking these traits to the by‐catch incidence. First, it is hypothesized that dolphins might be mechanically affected by fishing gear: Their body size may determine how they become entangled in nets, with mesh size potentially having a selectivity effect, as suggested by Brown et al. (2014). Second, their feeding behavior may influence how they interact with fishing gears (Read et al., 2003; Santana‐Garcon et al., 2018) with differential risk depending on the overlap between the diet of targeted species and their diet (Brophy et al., 2009; Spitz et al., 2013). Finally, existing sex‐ or age‐specific social segregation patterns (Murphy et al., 2013) and related variations in behaviors (such as socializing, resting, spatial behavior, and foraging; Ball et al., 2017; Castro et al., 2020; Castro et al., 2022) or diet (Murphy et al., 2013) may account for differential phenotypic vulnerability to fishing techniques (e.g., Vishnyakova & Gol'din, 2015). Thus, behavioral differences in habitat use related to the individual's phenotype (e.g., nursery habitats, Castro et al., 2020; Castro et al., 2022; or depth‐dependent habitat use, Sprogis et al., 2018) could explain trait‐specific by‐catch risks.

This study investigated (i) the phenotypic sensitivity to by‐catch and (ii) the trait‐specific vulnerability to different fishing techniques of unintentionally captured individuals from two protected species of small cetaceans. We tested the above expectations and hypotheses from the literature, by (i) examining the spatiotemporal distribution of by‐caught individuals' phenotype and (ii) describing existing correlations between phenotypes of by‐caught individuals (sex, body size and mass) and fishing techniques used. To this end, we used by‐catch data from the French observation programme (OBSMER), French declaration data (skipper logbook), and strandings (French stranding network, coordinated by La Rochelle University via the Pelagis observatory) in the Northeast Atlantic waters (FAO area 27), between 2000 and 2023.

## MATERIALS AND METHODS

A graphical summary of the analytical framework is available in Appendix S1: Figure S1.

### Studied species and areas

This study focused on two protected species of small cetaceans: the common dolphin and the harbor porpoise. They were selected on the basis of data availability (number of recorded by‐catches with available data on phenotypes; Appendix S1: Section S1). Only these two species had sufficient sample sizes to conduct statistical analyses (individuals with data on both phenotypes and fishing techniques: N=365 and N=113 for common dolphins and harbor porpoises, respectively). These are indeed the species of greatest local conservation concern, as they are the most frequently by‐caught species in the areas studied (ICES, 2019, 2020, 2022b, 2023; Taylor et al., 2022).

Common dolphins are widespread in the Northeast Atlantic, inhabiting the continental shelf year‐round (Certain et al., 2008, 2011; Lambert et al., 2018), and oceanic waters in summer (Lambert et al., 2017; Gilles et al., 2023). These dolphins can form large social groups (up to several thousand individuals), with some sex and age segregation, particularly outside the mating period (Murphy et al., 2013). The Northeast Atlantic population is estimated to be over 600,000 individuals (ICES, 2020). Their primary diet consists of small, fatty fish such as sardines and anchovies (Meynier et al., 2008), but they also prey on Gadiformes species such as hake, whiting and *Trisopterus* spp. in neritic areas (Brophy et al., 2009; Santos et al., 2013), as well as Myctophids in oceanic zones (Brophy et al., 2009; Spitz et al., 2010). By‐catch is recognized as a major threat to this species (Taylor et al., 2022). Stranding‐based estimates in the Bay of Biscay range from 3650 to 4700 by‐caught dolphins per year between 1997 and 2009 (Peltier et al., 2016), with more recent estimates exceeding 10,000 dolphins caught per year (ICES, 2020). Trammel nets, set gillnets, and pair‐trawls have been identified as particularly prone to by‐catch (ICES, 2019, 2020; Peltier et al., 2020, 2021; De Boer et al., 2012; Fernández‐Contreras et al., 2010). Common dolphins are frequently observed interacting with fishing gears, including depredation interactions (e.g., Lauriano et al., 2009; Milani et al., 2019 for gillnets; De Boer et al., 2012; Fertl & Leatherwood, 1997; Giménez et al., 2021 for trawls).

The harbor porpoise is a common cetacean in the Northeast Atlantic, inhabiting cold temperate and subpolar waters. It is found primarily on continental shelves and in shallow nearshore waters less than 200 m deep, although it also navigates deeper waters (Bjørge & Tolley, 2009; IAMMWG et al., 2015; Jefferson & Curry, 1994). Harbor porpoises are solitary or occur in small groups of about two individuals (Torres Ortiz et al., 2021). The latest abundance estimate in the Northeast Atlantic (excluding southern Ireland) was around 400,000 individuals in summer 2022 (Gilles et al., 2023), to which approximately 10,000 individuals from southern Ireland must be added (Giralt Paradell et al., 2024). In French waters, abundance estimates varied from 2724 to 13,358 (winter to summer) in the Bay of Biscay and from 17,829 to 18,429 (winter to summer) in the English Channel (Laran et al., 2017). The species primarily feeds on herring, anchovy, sprat, sand eel, gobies, cod, and other gadoids (Bjørge & Tolley, 2009; IAMMWG et al., 2015; Santos & Pierce, 2003; IMR/NAMMCO, 2019). By‐catch is a significant threat to the species (Caswell et al., 1998; IAMMWG et al., 2015; ICES, 2020; Taylor et al., 2022), particularly in static nets (IAMMWG et al., 2015; ICES, 2020; Jefferson & Curry, 1994). Interactions with fishing gear, particularly depredation, have been observed in both nets (Maeda et al., 2021; Milani et al., 2019) and trawls (Fertl & Leatherwood, 1997).

The available by‐catch data, as shown in the next section, originated from the activity records of the French fishing fleet and stranding records on the French coasts. The reported by‐catch events are mainly from the Bay of Biscay (primarily in the 27.8.a and 27.8.b ICES divisions: Appendix S1, Figure S2) and the English Channel (27.7.e and 27.7.d ICES divisions, Appendix S1: Figure S2). However, we also analyzed data from nearby areas with more limited data availability (Appendix S1: Figure S2), such as the Celtic Sea (27.8.f, 27.8.g, 27.8.h divisions), South‐West Ireland (27.7.j and 27.7.k divisions), and the southern North Sea (27.4.c division). We have provided detailed information on the distribution of by‐catches by area and period in Appendix S1: Section S2 and Figures S3–S7.

### Data: Sources and retained fishing techniques

Here, we selected characteristics of fishing techniques that have been previously identified as high risk of marine mammal by‐catch (Brown et al., 2013; Northridge et al., 2017) and are available in our datasets: fishing gear (gillnet or trawl), mesh size, presence of acoustic deterrents, targeted or fished taxa, and fishing effort (time spent fishing and volume of catch). Data were obtained from three monitoring programmes: direct declaration by fishermen, onboard observations, and stranding observations.

#### Declaration data

We retrieved by‐catch data declared by skippers between 2019 and 2023. These data are available in the SACROIS workflow from the French SIH system (“Système d'Information Halieutique,” i.e., Fishing Information System; Leblond et al., 2008). The reporting of cetacean by‐catch in logbooks has been mandatory in France since 2011, but the related data flow has only been operational since 2019. We only retained the declarations of single‐individual by‐catch events as only these events were associated with individualized phenotype data and could therefore be used for statistical analyses. This dataset contains a large number of by‐catch events with exploitable data on by‐caught individuals' phenotype (N=170, both studied species combined). Yet, it provides little information on fishing techniques. Information on the fishing gear used (type, mesh size) was provided in most cases, and the targeted taxon was often reported (in 75% of cases, see Appendix S1: Section S3). These three variables were retained for use in all analyses using these declaration data. All records potentially present in both the declaration dataset and the observation programme were discarded from the declaration dataset (as more information is available in the observation dataset; see Appendix S1: Section S3).

#### Onboard observation data

We retrieved by‐catch observation data from the OBSMER observation programme (https://archimer.ifremer.fr/doc/00774/88640/). This programme relies on the voluntary participation of skippers who agree to carry an observer on board during the entire fishing trip. All fisheries are sampled during this programme in proportion to their size, except for oversampled fisheries. Since 2020, sampling has been random, but subject to skippers' subsequent agreements. Sampling is stratified by area, time (by quarter), vessel size class, and fleet (towed or passive gears). However, some fisheries, particularly pelagic trawlers since 2018, have been oversampled compared to others due to the identification of a higher risk of by‐catch in the latter. Details of the OBSMER sampling scheme can be found in Cloâtre et al. (2022) and Cloâtre et al. (2023). Data were collected between 2005 and 2023, resulting in a total sample of 308 by‐caught individuals with phenotypic measurements for the two studied species. This programme collects a significant amount of information on fishing techniques. It does so by recording onboard information about the caught taxa and their associated mass (see Appendix S1: Section S3). In addition, skippers were interviewed about the fishing gear used, associated mesh size, fishing gear dimension, depth and speed of fishing gear, targeted taxa, duration of the fishing operation (i.e., soaking time), and presence of an acoustic deterrent device (ADD). ADDs were present in 19.2% of observed fishing operations with by‐catch (17.2% of missing data), either during trammel nets hauling (*N* = 15; mostly Cetasever PIFIL pingers: https://www.octech.fr/fr/produits-techniques-professionnels-de-la-peche/) or during pair‐trawling (*N* = 44; mostly STM Dolphin Dissuasive Devices 3: https://www.marintec.co.nz/dolphin-deterrent-devices). They were used according to the manufacturer's instructions, which can be found on the aforementioned websites, along with device specifications. Additional information about ADDs, including their precise distribution among gears, time periods, and areas, is provided in Appendix S1: Section S4.

Among these fishing techniques variables, we discarded the fishing gear speed variable as it was only available for trawls (48% of missing data). Additionally, we excluded from analyses metrics that were particularly well described by other retained variables (Kruskal–Wallis tests and Spearman correlation tests with all *p*‐values close to zero, i.e., $<2.2^{-16}$ in R software). In particular, fishing gear dimension strongly differed with the type of fishing gear (Kruskal–Wallis chi‐squared: 250), targeted taxa (Kruskal–Wallis chi‐squared: 226), and correlated strongly with mesh size (Spearman's rank correlation rho: 0.53). Fishing gear depth was also well described by the targeted taxa (Kruskal–Wallis chi‐squared: 194) and was associated with a significant amount of missing data (14%). Other remaining variables (namely: type of fishing gear, mesh size, most caught taxa, total catch mass, soaking time, and presence of an ADD) were used in all subsequent analyses using observation data only.

#### Stranding data

These data are collected daily by the French marine mammal stranding monitoring programme (“Réseau national d'échouages,” Canneyt et al., 2015), coordinated by the Observatoire Pelagis of La Rochelle University (LRUniv). The used dataset only included stranded individuals with external evidence of by‐catch (as described in Peltier et al., 2020). Hereafter, stranded individuals or strandings always refers to individuals with external evidence of by‐catch. Data spanned from 2000 to 2022 (N=3475 common dolphins and N=871 harbor porpoises): only stranded individuals with available phenotypic measurements (sex, length, and/or mass) and information on location and carcass decomposition (a proxy for the date of death) were retained for analyses. Stranding reporting rates are stable in France since the 1990s (Authier et al., 2014).

### Data: Phenotypic traits and external variables

The three datasets contained information on three by‐caught individuals' traits: sex, body mass (BM), and body length (BL). BL was measured (in centimeters) with a tape, from the tip of the rostrum to the median notch of the caudal fin. Sex was determined by assessing the presence of mammary slits and the relative position of genital slits and anus, following the method described in Van Canneyt et al. (2015). It is important to note that BM is not measured accurately: an “expert opinion” estimate is made in situ by the skipper for declarative data, or by observers in the OBSMER and stranding programmes (for the latter, a suspended scale was sometimes used to measure the exact mass where possible). BM estimates were included in the analyses to determine whether the results aligned with those from the BL analyses, thus assessing the usability of such simplified estimates. All traits values were preliminary checked to remove any aberrant measurement (Appendix S1: Section S1). In declarative data, only BM was available for all captured individuals, while the other two datasets (observation and strandings data, respectively) contained information on BM (61% and 10% of data, respectively) and/or BL (90% and 95% of data, respectively) and/or sex (63% and 90% of data, respectively). For individuals with measurements on multiple traits, BM and BL were found to be positively correlated in both species (Spearman correlation test, all *p*‐values <0.001, respective correlation coefficients of 0.60 for by‐caught common dolphin and 0.75 for by‐caught harbor porpoise, 0.82 for stranded common dolphin and 0.81 for stranded harbor porpoise). Considering observation data, median BM and BL of by‐caught individuals did not differ significantly between sexes for either species (Wilcoxon tests for comparing median values; for BL: *p* =0.24 for common dolphin, *p* =0.95 for harbor porpoise; for BM: *p* =0.92 for common dolphin, *p* =0.56 for harbor porpoise). For stranded individuals, median BM and BL were significantly different between sexes only for common dolphins (Wilcoxon tests; for BL: *p* <0.001 for common dolphin, *p* =0.1 for harbor porpoise; for BM: *p* <0.001 for common dolphin, *p* =0.76 for harbor porpoise), but with relatively small differences: Males were slightly larger (in average: 187 cm ±27 SD, 78 kg ±24 SD) than females (in average: 181 cm ±23 SD, 65 kg ±21 SD).

All datasets include information on the ICES division, year, and trimester in which the by‐catch occurred. For stranding data, we considered the ICES division where the body was found to be a sufficiently good proxy for the by‐catch area of origin. We also considered the trimester and year of stranded individuals' by‐catch to be those with the greatest overlap with the mortality interval estimate. The observation dataset also includes information on the sea conditions (Douglas sea state scale) during fishing operations (trawling or hauling for gillnets) and the time of the day when the operation ended. We used spatiotemporal variables (year, trimester, ICES divisions) to test for potential variation in patterns of phenotypic sensitivity to by‐catch and potential random effects in vulnerability to fishing techniques models (see next section).

### Data analyses

We performed statistical analyses and data handling using R software (R Development Core Team, 2008, version 4.4.1) and produced graphs using the ggplot2 package (Wickham, 2016).

#### Phenotypic sensitivity to by‐catch

**FIGURE 1:**
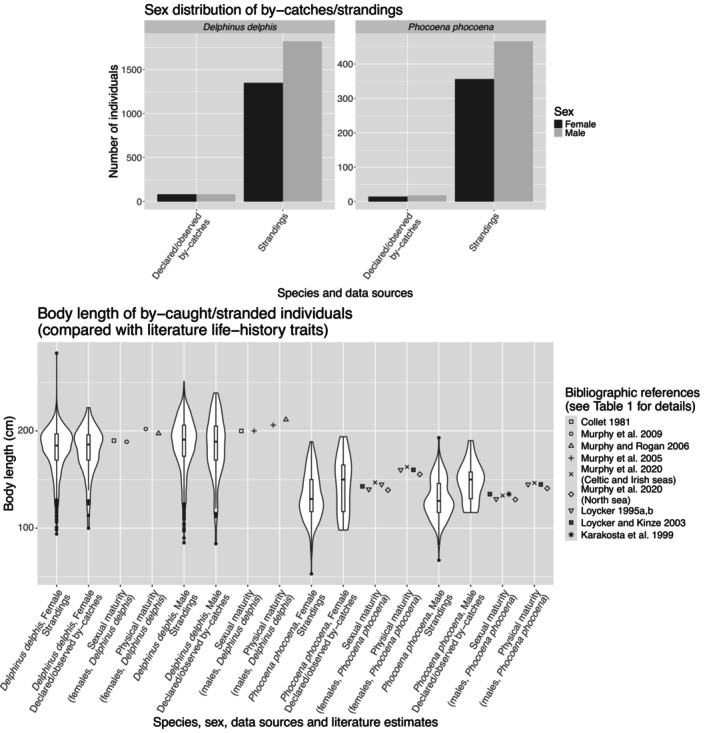
Distribution of by‐caught and stranded individuals' phenotypic traits. These figures show the distribution of body length (BL) and sex for by‐caught common dolphins and harbor porpoises (considering only stranded individuals with marks of incidental capture, and both observation and declaration by‐catch data). We displayed the distribution of BL using a combination of boxplots and violin plots for each sex. Data on the sex of by‐caught individuals are displayed using bar plots. For comparison, we plotted data from the literature on sizes at sexual and physical maturity. We jittered the latter points horizontally to improve readability. Please refer to Table 1 for the original publications and methods from which these estimates were obtained.

To explore phenotypic sensitivity to by‐catch (i.e., the likelihood of by‐catch as a function of individual traits), we first compared the trait values of by‐caught and stranded individuals with population values of biological parameters reported in the literature (BL at sexual maturity and asymptotic adult BL, i.e., BL at physical maturity), for each species and both sexes. We used one‐sample Wilcoxon tests to compare the median BL values with the reference values from the literature (i.e., to test the symmetry of the BL distribution relative to the reference point) and exact binomial tests to test for balanced sex ratios. We compiled the reference values of BLs at maturity in Table 1, along with the methods and data used to retrieve them. We only considered references that matched our management unit areas (the Northeast Atlantic for the common dolphin and the Celtic and North Seas for the harbor porpoise). References provided different estimates, depending on the methods, data sources, time periods or areas considered. We performed comparisons using the maximum and minimum estimates obtained from literature, for both stranding and by‐catch data (Figure 1). Note that we then used these estimates to compute the proportion of sexually mature individuals in the different studied categories.

**TABLE 1:** Estimates of biological parameters (body length, age at maturity) from the literature.

Species	Sex	Reference	Methods	Period	Area	Sample size	LSM	ASM	LPM	APM
Common dolphin	Male	[1] (in [2])	SOFI	…	Northeast Atlantic	…	200	5–7	…	…
		[3]	SOFI/Gompertz	1991–2003	Northeast Atlantic	184/170	200	11.9 (0.62)	206 (1.6)	12
		[4]	Gompertz	1990–2003	Ireland	103	…	…	211.6 (1.68)	11
	Female	[1] (in [2])	SOFI	…	Northeast Atlantic	…	190	6–7	…	…
		[5]	SOFI (BL) and GLM (age)/Richard	1990–2006	Northeast Atlantic	597/379/510	188.8 (0.02)	8.22 (0.26)	202 (1.17)	…
		[4]	Gompertz	1990–2003	Ireland	72	…	…	197.4 (1.69)	9
Harbor porpoise	Male	[6], *[7]* (in [8])	Minimum ASM and LSM/Mean adult BL	1985–1994 *1989–1997*	British Isles	114 (*51*)	130–*135*	3	145	…
		[9] (in [8])	Minimum ASM and LSM/Mean adult BL	1940–1998	North sea	135/338	135	3–4	145	…
		[10]	GLM/Gompertz	2000–2012	Celtic and Irish seas	164/66/83	133.46 (1.24)	3.62 (0.26)	146.5 (1.6)	7.62
					North sea	97/45/49	129.47 (1.29)	3.62 (0.26)	140.94 (1.64)	7.62
	Female	[6] (in [8])	Minimum ASM and LSM/Mean adult BL	1985–1994	British Isles	114	140–145	3–4	160	…
		[9] (in [8])	Minimum ASM and LSM/Mean adult BL	1940–1998	North sea	25/322	143	3.3	160	…
		[10]	GLM/Gompertz	2000–2012	Celtic and Irish seas	199/86/87	146.94 (1.32)	4.8 (0.31)	162.94 (1.95)	11.66
					North sea	90/49/51	139.18 (1.14)	4.8 (0.31)	155.37 (1.97)	11.66

*Note*: This table summarizes estimates of body length (BL) and age (A) at sexual maturity (SM) and physical maturity (PM) for the common dolphin in the northeastern Atlantic and the harbor porpoise in the Celtic and North Seas, as found in the literature. When available, we provided the standard error of the estimate in brackets after the estimated value. Along with the estimates, we provide the associated references, methods, periods, areas, and sample sizes. The table footnotes below provide references, along with data sources used for analyses when provided. For the methods, we first indicated the method used for estimations of BL at sexual maturity, followed by the method used for estimations at physical maturity (separated by a slash). If different methods were used with different sample sizes, we indicated the sample size in the same order in which we presented the methods (separated by slashes). When an estimate varied from the others in the data used, we indicated the associated data and estimate in italics. In reference $\left[5\right]$ (Murphy et al., 2009), BL at physical maturity (asymptotic BL) is provided using different statistical methods: We only presented there the most parsimonious one. In reference $\left[10\right]$ (Murphy et al., 2020) estimates are provided for two study periods (1990–1999 and 2000–2013). We only retained results from the most recent period because we only analyzed individuals that were incidentally captured after 2000. For harbor porpoises, we presented estimates from the Celtic Sea and the North Sea, as individuals from both areas were included in our study. We did not consider other areas because French porpoises are mainly related to individuals from these areas (Alfonsi et al., 2012), and because we observed no size differences between stranded individuals from the Celtic Sea and the Bay of Biscay (Figure 3). $\left[1\right]$ Collet (1981) (Ph.D. dissertation); $\left[2\right]$ Perrin and Reilly (1984); $\left[3\right]$ Murphy et al. (2005): French and Irish strandings and by‐catches; $\left[4\right]$ Murphy and Rogan (2006): Irish strandings and by‐catches; $\left[5\right]$ Murphy et al. (2009): UK, Irish, French, Galician and Portuguese strandings and by‐catches; $\left[6\right]$ Lockyer (1995a, 1995b): UK strandings and by‐catches; $\left[7\right]$ Karakosta et al. (1999): UK strandings and by‐catches; $\left[8\right]$ Lockyer (2003), $\left[9\right]$ Lockyer and Kinze (2003): Danish strandings, directed catches and by‐catches; $\left[10\right]$ Murphy et al. (2020): UK strandings and by‐catches. “…”, missing data in the original reference.

Abbreviations: APM, age at physical maturity; ASM, age at sexual maturity; GLM, generalized linear model (on mean length/age when 50% are mature); Gompertz, gompertz growth curve; LPM, length at physical maturity; LSM, length at sexual maturity; Richard, Richard growth model; SOFI, sum‐of‐fraction of immature method.

We then examined whether different phenotypic sensitivity profiles emerged in space or time by implementing a generalized linear model for each of the studied traits (linear model for BL, logistic regression for sex) as a function of year, trimester, and ICES division of by‐catch. We considered separate BL models for each sex to account for potential sex‐specific sensitivity patterns. These models were only conducted for stranding data, because the low number of by‐catch observations and declarations often resulted in over‐parametrised models when using the other datasets. Only models on BL were run because this trait was systematically measured for stranded individuals, unlike BM for which sample sizes were too small (about 92% and 84% of missing values for common dolphins and harbor porpoises, respectively). For observed/declared by‐catch, we then explored the presence of these spatiotemporal phenotypic profiles by using nonparametric tests (Kruskal–Wallis test for studying the relationship between spatiotemporal covariates and BM or BL, Fisher test for studying the relationship between spatiotemporal covariates and sex). To avoid excessive parameters‐to‐data ratio, we coded categories to ensure a relative balance with respect to sample size. Thus, for stranding data, we considered periods using four‐year group intervals (except for the most recent group, which consisted of the three most recent years). For observed/declared by‐catch, data were more fragmented and we therefore grouped close years and ICES divisions into sets of different lengths (see Appendix S1: Section S5).

#### Phenotypic vulnerability to different fishing techniques

We then assessed the effect of fishing techniques on by‐caught phenotypes (referred to as the phenotypic vulnerability to by‐catch) for either species. We primarily considered the observation dataset, which provides detailed descriptions of fishing techniques, sexes, and precise BL measurements. We tested the effect of various factors, including fishing gear, mesh size, fished taxa, presence of deterrent devices, soaking time, and catch mass, on both BL and the sex of by‐caught individuals. Of note, we also studied the effect of fishing gear, mesh size, and targeted taxa on the loweraccuracy “guesstimated” BM, using both declaration and observation data sources, as the response variable. This was done to check for alignments with former BL models. We implemented linear regressions for BM and BL models, and logistic regressions for sex models. The first step of these analyses involved formatting the explanatory variables used in our models (Appendix S1: Section S6 and Figures S8–S10). Briefly, we pooled sparse categories where necessary and possible in whole datasets, excluded the rarest categories (or groups of categories) from each data subset used for analyses, removed variables with too much multicollinearity, and tested for the inclusion of potential random effects of spatiotemporal variables (range of years, trimester, ICES division) in the models used. We discarded from the analyses any observations for which at least one of the retained variables was missing.

#### Model selection and checking

For each model, we conducted a preliminarily testing of collinearity using the generalized variance inflation factor (GVIF) from the vif function in the car R package (Fox & Weisberg, 2019). More specifically, when models included factor variables with more than two categories, ${\text{GVIF}}^{1/\left(2\mathrm{df}\right)}$ was used: a value greater than 2.24 (i.e., a GVIF >5 for one‐df variables) was the cut‐off. In such cases, we removed the variable with the highest ${\text{GVIF}}^{1/\left(2\mathrm{df}\right)}$ (or GVIF) value. All these values were found to be low enough (≤1.05) to run our models on phenotype sensitivity without any correction. We provided GVIF values associated with phenotype vulnerability models in Appendix S1, Table S1. For nonparametric tests used in phenotype sensitivity analyses, we checked for collinearity between pairs of variables using Fisher tests.

**FIGURE 2:**
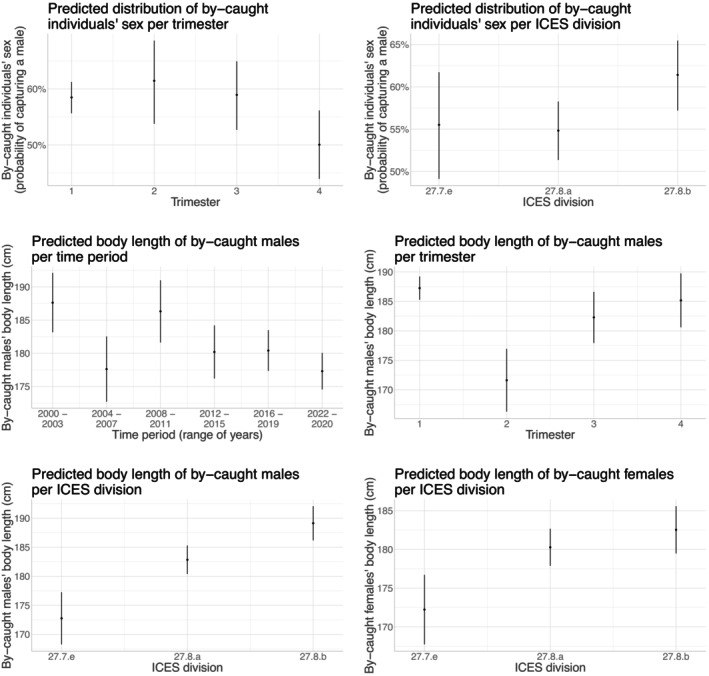
Spatiotemporal variations in stranded common dolphins' phenotype. The figures show the marginal effects of the spatiotemporal variables (ICES division, trimester, range of years) on the sex (logistic regression: first two graphs) or body length (BL; linear regression) of stranded common dolphins with external evidence of by‐catch. Results were obtained using the ggemmeans function of the ggeffects R package (Lüdecke, 2018). Dark dots indicate the predicted value of BL or sex probability as a function of spatiotemporal variables. Error bars indicate the CIs based on standard errors, assuming a normal distribution. Note that we generated these graphs from models with untransformed variables, but when we used transformed variables, we obtained nearly identical graphs.

**FIGURE 3:**
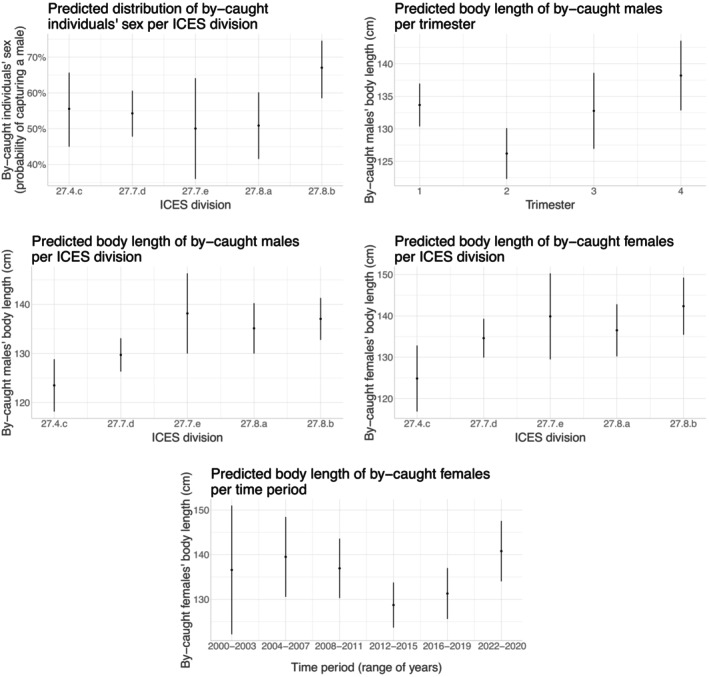
Spatiotemporal variations in stranded harbor porpoises' phenotype. The figures show the marginal effects of the spatiotemporal variables (ICES division, trimester, range of years) on the sex (logistic regression: first graph) or body length (BL; linear regression) of stranded harbor porpoises with external evidence of by‐catch. Results were obtained using the ggemmeans function of the ggeffects R (Lüdecke, 2018). Dark dots indicate the predicted value of BL or sex probability as a function of spatiotemporal variables. Error bars indicate the CIs based on standard errors, assuming a normal distribution. Note that we generated these graphs from models with untransformed variables, but when we used transformed variables, we obtained nearly identical graphs.

**FIGURE 4:**
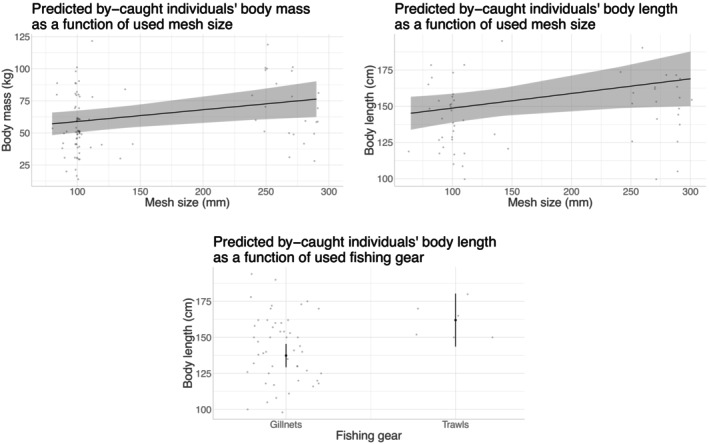
Effects of fishing gear and mesh size on body mass (BM) and body length (BL) of by‐caught harbor porpoises. The figures show the marginal effects of fishing gear and mesh size on by‐caught harbor porpoises' or BL. Results were obtained from linear regressions on observation data, using the ggemmeans function of the ggeffects R (Lüdecke, 2018). The original data points are shown in gray and slightly jittered (small amount of random variation in the location of the data points, to avoid overplotting) to improve readability. The predicted value of BM or BL is shown as dark lines or dots, as a function of mesh size or fishing gear, respectively. Gray areas and error bars indicate CIs based on standard errors, assuming a normal distribution.

**FIGURE 5:**
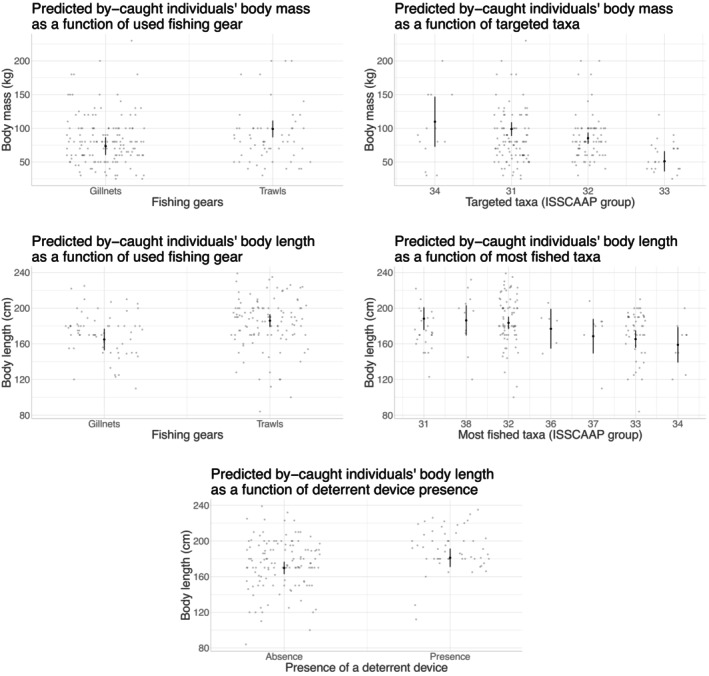
Effects of fishing gear and fished/targeted taxa on body mass (BM) and body length (BL) of by‐caught common dolphins. The figures show the marginal effects of fishing gear and fished/targeted taxa on the BM or BL of by‐caught common dolphins. Results were obtained from linear regressions on observation data, using the ggemmeans function of the ggeffects R (Lüdecke, 2018). The original data points are shown in gray and slightly jittered (small amount of random variation in the location of the data points, to avoid overplotting) to improve readability. Dark dots indicate the predicted value of BM or BL as a function of fishing gear or fished/targeted taxa. Error bars indicate the CIs based on standard errors, assuming a normal distribution. ISSCAAP, International Standard Statistical Classification of Aquatic Animals and Plants (from FAO), 31—Pleuronectiformes (mainly soles in our case)/32—Gadiformes (mainly hake in our case)/33—Miscellaneous coastal fishes (mainly sea bass in our case)/34—Miscellaneous demersal fishes (mainly monkfishes in our case)/36—Tunas, bonitos/37—Miscellaneous pelagic fishes (mainly mackerels in our case)/38—Sharks, rays.

Significance testing (analysis of variance) used the car R package (Fox & Weisberg, 2019) and was reported in Tables 2 and 3. Only statistically significant effects at the 5% level were interpreted (Figures 2, 3, 4, 5) and subsequently discussed. For categorical variables with significant effects, we performed post hoc pairwise comparison tests using estimated marginal means (the other variables being held at their average values) with the emmeans R package (Lenth, 2024), using Tukey's correction for multiple testing (Appendix S1: Tables S2–S4). In the specific case of logistic regression, the comparison was performed on the log odds ratios (z‐tests). We plotted the predicted traits distributions to illustrate the marginal effect of significant variables (all other variables being held at their average values), using the ggeffects R package (Lüdecke, 2018, ggemmeans function).

**TABLE 2:** Analyses of variance of spatiotemporal models on the phenotype of stranded individuals.

	df	Common dolphin			Harbor porpoise		
		Sex (logistic regression)	Males BL (linear regression)	Females BL (linear regression)	Sex (logistic regression)	Males BL (linear regression)	Females BL (linear regression)
		LR χ^2^ (*p*‐value)	*F* value (*p*‐value)	*F* value (*p*‐value)	LR χ^2^ (*p*‐value)	*F* value (*p*‐value)	*F* value (*p*‐value)
Time period (range of years)	5	1.51 (0.912)	**5.99 (<0.001** *** **)/*5.9 (<0.001)***	1.21 (0.301)/*1.11 (0.351)*	9.74 (0.083)	0.582 (0.714)/*0.578 (0.717)*	**2.58 (0.0264** * **)/*2.88 (0.0146)***
ICES division	2	**10.1 (<0.001** *** **)**	**23.6 (<0.001** *** **)/*24.3 (<0.001)***	**8.78 (<0.001** *** **)/*7.52 (<0.001)***	**10.3 (0.0356** * **)**	**6.1 (<0.001** *** **)/*6.15 (<0.001)***	**3.49 (0.00824** ** **)/*3.39 (0.00982)***
Trimester	3	**8.05 (0.0451** * **)**	**12 (<0.001** *** **)/*9.96 (<0.001)***	1.23 (0.297)/*1.05 (0.371)*	3.56 (0.313)	**6.28 (<0.001** *** **)/*6.16 (<0.001)***	1.76 (0.154)/*1.93 (0.125)*
*N*		3162	1754	1300	820	450	335
*R* ^2^		0.0136	0.0787/*0.0758*	0.0261/*0.0228*	0.0434	0.107/*0.108*	0.11/*0.115*

*Note*: The table shows the results of analyses of variance for all models of phenotypic sensitivity spatiotemporal patterns (sex: logistic regression, BL: linear model). We conducted these models using stranding data, for each species studied, as well as for each sex for BL sensitivity patterns. For each spatiotemporal variable used in the model (ICES division, range of years, trimester; see left part of the table), we report the df, the *F* value (for linear models) or the likelihood ratio chi‐squared statistic (for logistic regressions) and, in brackets, the corresponding *p*‐values together with their level of significance (see scale below). We have highlighted in bold the statistics associated with significant *p*‐values (<0.05). We also provided these metrics for modified models (shown in italics after the slash symbol) that accounted for deviations from model assumptions, with cubic transformations for the common dolphin BL models and square root transformations for the harbor porpoise BL models. We found no changes in the significance level or direction of the effects after variable transformation. For each model, we also reported in the lower part of the table the number of observations used and the model fit, that is, the *R* ^2^ (Nagelkerke pseudo *R* ^2^ for logistic regression). Again, we have provided these latter metrics for modified models, with the same labels as above.

Abbreviations: BL, body length; LR χ^2^, likelihood ratio chi‐squared statistic; *N*, number of observations.

*** *p* < 0.001;

** *p* < 0.01;

* *p* < 0.05.

**TABLE 3:** Analyses of variance of vulnerability models (fishing techniques effects on the phenotype of by‐caught individuals).

	df	Common dolphin			Harbor porpoise		
		Sex (logistic regression)	Body mass (linear regression)	Body length (linear regression)	Sex (logistic regression)	Body mass (linear regression)	Body length (linear regression)
		LR χ^2^ (*p*‐value)	*F* value (*p*‐value)	*F* value (*p*‐value)	LR χ^2^ (*p*‐value)	*F* value (*p*‐value)	*F* value (*p*‐value)
Used fishing gear	1	0.00763 (0.93)	**11.6 (<0.001** *** **)/*12.7 (<0.001*** *** ***)***	**10.5 (0.00146** ** **)/*12.7 (<0.001*** *** ***)***		1.21 (0.275)	**5.73 (0.0205** * **)**
Mesh size	1	**5.57 (0.0183** * **)**	0.0726 (0.788)/*0.0549 (0.815)*	0.0422 (0.837)/*0.233 (0.63)*		**7.69 (0.00676** ** **)**	**4.55 (0.0379** * **)**
Targeted taxa (ISSCAAP code)	3		**8.59 (<0.001** *** **)/*9.26 (<0.001*** *** ***)***				
Fished taxa (ISSCAAP code)	3‐6‐1	2.57 (0.463)		**3.32 (0.00409** ** **)/*3.82 (0.00136*** ** ***)***	0.176 (0.675)		
Soaking time	1	2.58 (0.108)		0.593 (0.442)/*0.478 (0.49)*	1.25 (0.263)		0.448 (0.506)
Total catch mass	1	**5.84 (0.0157** * **)**		0.887 (0.348)/*0.505 (0.478)*	1.51 (0.219)		0.0653 (0.799)
Presence of an acoustic deterrent	1	0.494 (0.482)		**5.82 (0.0169** * **)/*5.41 (0.0212*** * ***)***			
*N*		124	207	178	17	93	54
*R* ^2^		0.175	0.123/*0.131*	0.208/*0.226*	0.256	0.0816	0.161

*Note*: The table shows the results of variance analyses for all phenotypic vulnerability models (sex: logistic regression, BM/BL: linear model), performed on both studied species. For each fishing activity variable used in the model (left part of the table), we provided the df, the *F* value (for linear models) or the likelihood ratio chi‐squared statistics (for logistic regressions) and, in brackets, the corresponding *p*‐values together with their level of significance (see the scale below). Of note, depending on models, different df were associated with the most fished taxa variable (due to variations in rare categories discarded from the analyses). We provided the different successive values (separated by dashes). We highlighted in bold statistics associated with significant *p*‐values (<0.05). We also provided these metrics for modified models (shown after the slash symbol, in italics) that accounted for deviations from model assumptions, with squared transformations for BL models and square root transformations for BM models, in common dolphins only. We found no changes in the significance level or direction of effects after variable transformations. For each model, we also reported in the lower part of the table the number of observations used and the model fit, that is, the *R* ^2^ (Nagelkerke pseudo *R* ^2^ for logistic regression). Again, we provided these latter metrics for modified models, with the same labels as above.

*** *p* < 0.001;

** *p* < 0.001;

* *p* < 0.05.

Abbreviations: BL, body length; BM, body mass; ISSCAAP, International Standard Statistical Classification of Aquatic Animals and Plants (from FAO); LR χ^2^, likelihood ratio chi‐squared statistic; *N*, number of observations.

Model fit was assessed using *R* ^2^ metrics (Nagelkerke pseudo *R* ^2^ for logistic regressions, Nagelkerke, 1991), reported in Tables 2 and 3. Compliance with model assumptions was checked graphically using the DHARMa R package (Hartig, 2022), based on analysis of the distribution of simulated residuals for perfect model fit. When we found a significant deviation from a uniform distribution of simulated residuals (similar to testing for normality of residuals) or between observed and predicted residuals distribution (similar to testing for heteroscedasticity), we applied data transformation to meet expectations (square root and square or cubic transformation for positively and negatively skewed data, respectively). After such transformations, we observed no further deviations from the assumptions. The statistics obtained after variable transformation are shown in Tables 2 and 3, and Appendix S1: Tables S2–S4. Results were nearly identical before and after transformations. We also assessed the presence of influential values graphically and caveated interpretations accordingly. Plots illustrating effects were produced using untransformed data (see Appendix S1: Figure S11 for plots with transformed data).

## RESULTS

A synthesis of the main results from the phenotype sensitivity and vulnerability analyses is provided in Table 4. In addition to the figures provided to illustrate our main results, we produced in Appendix S1: Tables S5–S8 descriptive tables for each analysis on BL with statistically significant effects of categorical variables. These tables summarize information on BL within each category, including range, median, observed and predicted mean values with standard errors, age predicted from median BL or predicted marginal mean, and the proportion of sexually mature individuals in the category.

**TABLE 4:** Summary of results from analyses of phenotypic sensitivity and vulnerability to by‐catch.

By‐catch risk analysis	Covariates	Common dolphin			Harbor porpoise		
		Sex	BM	BL	Sex	BM	BL
Sensitivity analyses (By‐catch ~ Traits)	None	Males > Females***	…	BL < BL at maturity***	Males > Females***	…	BL < BL at maturity**
	Spatial patterns	Males increase in south BoB***	…	BL decreases in Channel sea***	Males increase in south BoB*	…	BL decreases in North sea**
	Year period patterns		…	Male BL decreases with years***		…	Females BL decreases in 2012–19*
	Trimestrial patterns	Males decrease in winter*	…	Male BL decreases in spring***		…	Male BL decreases in spring***
Vulnerability analyses (by‐catch traits ~ Fishing techniques)	Fishing gear		BM increases in trawls**	BL increases in trawls**	…		BL increases in trawls*
	Mesh size				…	BM increases with mesh size**	BL increases with mesh size*
	Targeted/Fished taxa		BM increases in sole/ hake fisheries***	BL increases in sole/ hake fisheries**		…	…
	Acoustic deterrent?		…	BL increases in presence of deterrent*		…	

*Note*: This table presents a synthesis of the results of all statistical analyses conducted to test the by‐catch phenotype sensitivity (i.e., the likelihood of by‐catch as a function of individuals' traits) or vulnerability (i.e., trait‐specific by‐catch vulnerability to fishing techniques). To simplify the presentation of the results, we have only presented here sensitivity analyses using stranding data (much more robust than analyses using declared/observed data: see *Materials and Methods*) and covariates from vulnerability models with at least one significant effect on the traits of by‐caught individuals. Vulnerability analyses on BM were conducted using both observation/declaration data, while analyses on sex and BL were conducted using observation data only. First sensitivity analyses, without covariates, used nonparametric comparison tests (Fisher test for sex, Wilcoxon test for BL): All methods and detailed statistics can be found in the Methods and Results sections. For all other analyses, (generalized) linear models were used: All methods and detailed statistics can be found in Methods and Tables 2 and 3. Ellipses indicates that the test was not performed for methodological reasons (too high collinearity, not enough data to analyze, too imprecise data; see Methods and Appendix S1: Section S6). We indicated the direction of the effects (Figures 1, 2, 3, 4, 5, Appendix S1: Tables S2–S4) when the value (BL, BM, proportion of males) increased and when it decreased. We indicated the level of significance of the effects (see scale below). For sensitivity analyses, when significant effects on male and female BL were similar, we only indicated the lowest significance level between the two.

*** *p* < 0.001;

** *p* < 0.01;

* *p* < 0.05.

Abbreviations: BL, body length; BM, body mass; BoB, Bay of Biscay.

### Phenotypic sensitivity to by‐catch

In both species, we found more stranded males than females with external evidence of by‐catch (exact binomial tests, *p* <0.001 for both common dolphins and harbor porpoises; Figure 1). Reported by‐catches involved about as many males as females, as illustrated by an even sex ratio (exact binomial tests, *p* =1 for common dolphins, *p* =0.60 for harbor porpoises). Median BL of by‐caught common dolphins of both sexes was significantly lower than (i) the BL at physical maturity (Wilcoxon tests, *p*‐values <0.001 for males and females, for both strandings and observed/declared by‐catch data; Figure 1), and (ii) the BL at sexual maturity (Wilcoxon tests, *p*‐values <0.001 for males, ≤0.009 for females, for both strandings and observed/declared by‐catch data; Figure 1). Thus, by‐caught common dolphins data always comprised fewer than 46% of sexually mature individuals (up to only 28–31% for males; Appendix S1: Table S5). For harbor porpoises, median BL of both sexes was smaller than BLs at sexual and physical maturity, but only in stranding data (Wilcoxon tests, *p*‐values <0.004, Figure 1; except when comparing males median BL to the lowest estimate of BL at sexual maturity, *p* =0.48). Thus, by‐caught harbor porpoises stranding data always comprised fewer than 46% of sexually mature individuals (up to only 24%–29% for females; Appendix S1: Table S5). In contrast, median BL did not significantly differ from size estimates at maturity in observed or declared by‐catch data (Wilcoxon tests, *p*‐values >0.05; Figure 1), except males median BL that were significantly larger than BL at sexual maturity (*p*‐value <0.032, >70% of sexually mature individuals; Figure 1 and Appendix S1: Table S5). There were no statistically significant sex differences in the mass and size class distributions of by‐caught individuals (see Appendix S1: Figures S12 and S13, for both data sources).

We provided model results on spatiotemporal sensitivity patterns among stranded individuals in Table 2 and illustrated statistically significant results in Figures 2 and 3 for common dolphin and harbor porpoise, respectively. In common dolphin, more by‐caught females were reported in autumn (fourth trimester; Appendix S1: Table S2) and in the northern part of the Bay of Biscay (division 27.8.a, especially when compared to the southern division 27.8.b; Appendix S1: Table S2). BL of stranded males decreased over the years (except for the period 2004–2007 with particularly small‐sized individuals, comparable to the most recent years; Appendix S1: Table S3). Average male BL was smaller in spring (second trimester) and then increased with a peak in winter, especially when compared to summer (i.e., first trimester compared to third; Appendix S1: Table S3). There was a latitudinal gradient in male BL, increasing towards the south of the distribution (Appendix S1: Table S3). Female BL was higher in the Bay of Biscay than in the English Channel (Appendix S1: Table S3). In harbor porpoises, as already found in common dolphins, more males were by‐caught in the south of the Bay of Biscay (Appendix S1: Table S2), with stranded males again being smaller in spring and both males and females being smaller in the northern part of their distribution (particularly in the North Sea, but also when comparing the eastern English Channel with the southern Bay of Biscay; Appendix S1: Table S3). Finally, BL of stranded females was smaller between 2012 and 2019, especially when compared to recent years (Appendix S1: Table S3). A decrease in BL between categories was always associated with a decrease in the proportion of sexually mature individuals and in the estimated age at the median BL (see Appendix S1: Tables S6 and S7).

Looking at spatiotemporal patterns from observed/declared by‐catch data (nonparametric tests: Appendix S1: Table S9), there were significant time period effects on BM of by‐caught common dolphins. In contrast to stranding data, BM of by‐caught individuals increased in the most recent years (Appendix S1: Figure S14). The reported size of by‐caught individuals was lower during the second trimester compared to others (Appendix S1: Figure S14), a pattern similar to that seen in stranding data. Although not significant, the proportion of by‐caught female common dolphins increased in the northern part of the study area (Appendix S1: Figure S15), in line with the stranding data.

### Phenotypic vulnerability to fishing techniques

Harbor porpoises were primarily by‐caught in trammel nets (Appendix S1: Figure S8), with a low proportion of reported events also occurring in set gillnets and few reported events in trawls. Reported by‐catch events of common dolphins were more evenly distributed between trawls (mainly pelagic pair‐trawls) and gillnets (approximately three‐quarters in trammel nets and one‐quarter in set gillnets). On the other hand, reported by‐catch events of harbor porpoises occurred mainly when targeting/catching sole or monkfish (Appendix S1: Figures S9 and S10), while those of common dolphins were mainly associated with vessels targeting sole, hake, or sea bass.

Analysis of variance results on how fishing methods related with the phenotypic traits of by‐caught individuals are provided in Table 3, and statistically significant effects are shown on Figures 4 and 5 for harbor porpoise and common dolphin, respectively. Analyses from the pooled observation/declaration datasets and the observation data alone showed that BM and BL of by‐caught harbor porpoises increased with mesh size. For both common dolphins and harbor porpoises, larger individuals (with higher BL, and also BM for common dolphins only) were by‐caught in trawls compared to gillnets. In the case of common dolphins, BM and BL of by‐caught individuals increased when fishing/targeting sole and hake (or other Gadiformes) compared to coastal fishes such as Sparidae or sea bass (Appendix S1: Table S4; Figures S9 and S10, for correspondences between targeted/fished ISSCAAP groups and targeted/fished taxa). Targeting Sparidae and sea bass was associated with significantly lower BM compared to all other targeted species (Appendix S1: Table S4). In addition, fishing predominantly for monkfish was associated with smaller BL of by‐caught dolphins compared to predominantly fishing for Gadiformes, soles, or sharks and rays (Appendix S1: Table S4), the latter being also associated with larger BL than fishing for coastal fishes. Larger common dolphins were also by‐caught in the presence of an ADD when hauling or trawling. Finally, we found the probability of incidentally capturing female common dolphins to increase with the fishing operation's total catch mass and to decrease with the mesh size (Appendix S1: Figure S16). However, these effects were fragile as they hinged strongly on high leverage points: a single by‐catch event of nine females, during a fishing operation associated with the highest total catch mass of the observations (5374 kg, far above other values: 992 ± 1462 SD), and a by‐catch event of a female with the lowest mesh size of the observations (50 mm, far below other values: 99 ± 11 SD). Excluding these events, the effects disappeared (respective *p*‐values of 0.80 and 0.056): We therefore did not interpret them further. No other significant effect of fishing activity metrics on sex was found for either species. Model fits were rather low for all models (Table 3; $R^{2}$ < 0.26), but more especially for models on BM, using both observation and declaration data ($R^{2}$ < 0.13).

## DISCUSSION

This study leveraged three complementary datasets to provide the most up‐to‐date and comprehensive investigation on the phenotypic correlates of by‐catch in two species of small cetaceans that benefit from a regime of strict protection under the Habitats Directive (92/43/EEC) in the European Union. Despite this protection level, by‐catch monitoring remains from largely convenience samples and strandings. Taking stock of these caveats, we nevertheless uncovered patterns that both confirm and expand previous knowledge.

### Sensitivity and vulnerability profiles to by‐catch

#### Phenotypic sensitivity to by‐catch

This study revealed that common dolphins and harbor porpoises by‐caught in fisheries flying the French flag and operating in the Northeast Atlantic (for observed/declared data) or active in an area relatively close to the French coast (for stranding data) represent a specific subset of their populations. By‐caught common dolphins were significantly smaller than the mean BL of sexually mature individuals or the asymptotic BL of adult individuals. Similarly, stranded harbor porpoises diagnosed as by‐caught were significantly smaller than the asymptotic BL of adult individuals and, in most cases, than the mean BL of sexually mature individuals. This suggests that a large part of incidental catches involves juveniles, that is, sexually and physically immature individuals (less than 12 or 8 years old for male or female common dolphins, and less than 4 or 5 years old for male or female harbor porpoises; as detailed in Table 1). This result is consistent with other studies such as those of Murphy and Rogan (2006), Brown et al. (2014), and Mannocci et al. (2012) in common dolphins or Brennecke et al. (2021) and Torres‐Pereira et al. (2023) in harbor porpoises. For stranded individuals only, we also found more males being by‐caught than females, for both species. Male‐biased sensitivity to by‐catch has been frequently described in common dolphins (Brown et al., 2014; Fernández‐Contreras et al., 2010; López et al., 2002; Westgate & Read, 2007; McGovern et al., 2018) and also mentioned in harbor porpoises (Torres‐Pereira et al., 2023). Differential sex‐ and size‐specific sensitivity to by‐catch can result from several nonexclusive factors (Brown et al., 2014): It could reflect the sex/age structure of the global population, spatial or temporal sex/age segregation (distribution of juveniles or males potentially overlapping areas and periods of high‐risk fishing fleet activity), or specific sex‐ or age‐related behaviors leading to differential sensitivity to by‐catch. Juveniles, potentially inexperienced, could engage in riskier interactions with fishing gear or lack physical/acoustic skills to avoid entanglement; males could generally take more risks as observed in other mammalian species.

#### Trait‐specific vulnerabilities to fishing techniques

The reported BL and mass of by‐caught individuals also related to fishing techniques. First, for harbor porpoises only, the body size and mass of by‐caught individuals were positively correlated with mesh size, suggesting a differential selectivity with larger mesh sizes mechanically increasing the likelihood of by‐catching larger porpoises. This result is consistent with the hypothesis raised (but not tested) in Brown et al. (2014) that mesh size likely contributes to porpoise selectivity. As in this later study, we could draw a parallel with a case study on sharks (McAuley et al., 2007), with a similar relationship between the mesh size used and the size of sharks caught. Of note, mesh size also depended on targeted species, and an alternative explanation could be related to monkfish fisheries strategies overlapping with adult harbor porpoises' behavior (see Appendix S1: Section S7, for details on alternative, weakly supported explanations of observed effects).

For both harbor porpoises and common dolphins, we also found an effect of fishing gear type on the reported body size, with larger individuals by‐caught in trawls than in gillnets (Appendix S1: Table S8: less than 21% of sexually mature individuals captured in gillnets against between 20% and 54% in trawls). Such a difference between fishing gear types has already been discussed in De Boer et al. (2012) for common dolphins, who argued for a higher vulnerability of calves and juveniles to gillnet by‐catch and of adult individuals to trawl by‐catch from the overlaps in the distribution of fisheries and age classes. Such a difference is indeed likely to be related to the age of the individuals, with younger (i.e., smaller; see Appendix S1: Table S8) individuals probably interacting with fishing gears differently than adults. The detection ability of the net could be lower for young individuals who acquire their echolocation skills during the first year of life, as was evidenced in bottlenose dolphin (Harder et al., 2016). As hypothesized by Murphy and Rogan (2006) and Murphy et al. (2013), juveniles could also lack experience in interacting with fishing gears. For example, social learning, including foraging strategies, is developed during the first years of life (Kuczaj II et al., 2012). Differential age‐vulnerability to by‐catch in trawls could then result from older individuals taking more risks by learning foraging techniques near or even within the trawl. This was hypothesized by Murphy et al. (2013) with the specific example of the pelagic trawl fishery for sea bass and observed in bottlenose dolphins (Santana‐Garcon et al., 2018).

Only in common dolphins was the size of by‐caught individuals larger in the presence of an ADD when hauling or trawling (Appendix S1: Table S8: less than 37% of sexually mature individuals captured in absence of a deterrent against between 29% and 50% in presence). This might suggest that adult individuals may be more habituated to the presence of acoustic deterrents than juveniles during these particular time periods. Acoustic deterrent habituation has been demonstrated in harbor porpoises (Carlström et al., 2009; Dawson et al., 1998; Cox et al., 2001), but only for short periods. There is currently no similar evidence in common dolphins (Carretta & Barlow, 2011). Adult individuals might even be lured by such an acoustic device acting as a “dinner bell” when hauling or trawling, as observed in pinnipeds for static nets (Dawson et al., 2013). Such behavior would be consistent with potential depredation occurring for the species studied, as suggested by multiple reports of feeding associated with trawling in the species studied (Fertl & Leatherwood, 1997; Gonzalvo & Carpentieri, 2023). It is worth noting that this effect was statistically robust. It appeared to be independent of the type of fishing gear or ADD used and insensitive to spatiotemporal potential confounding effects (see Appendix S1: Section S4 for details).

Finally, we found that larger common dolphins were by‐caught when targeting/fishing Pleuronectiformes (mainly soles) and Gadiformes (mainly hake) compared to coastal fishes (mainly sea bass and sea bream). This could be due to existing age‐related differences in diet (Murphy et al., 2013): Adult males prey on larger and less diverse prey, with a particularly low proportion of cephalopods; relative to females and juveniles whose diet is more diverse with, in particular, a high proportion of blue whiting preyed upon by juvenile males. However, these known intraspecific differences in diet are not particularly consistent with our results, suggesting that other mechanisms may be at play. Indeed, neither sea bream, nor sea bass, nor sole are preyed upon by common dolphins. A likely hypothesis is that the differences in by‐caught individuals' size as a function of targeted/fished species are due to the overlap between the habitat or diet of these species and the age‐specific habitat or diet of dolphins. For coastal fishes (sea bream, sea bass), there is likely to be an overlap in diet (Spitz et al., 2013), but also a spatial overlap with the dolphin age‐specific habitats, as groups of juveniles are more likely to be distributed inshore than adult groups (Cañadas & Hammond, 2008; De Boer et al., 2012). In this particular case, there is also likely to be a greater overlap with the diet of younger individuals, as the size of prey consumed by these coastal fishes is likely closer to the size of prey consumed by juveniles than adults. It is worth noting that similar observations of younger individuals being by‐caught when targeting seabass have already been highlighted in Murphy et al. (2013). Similarly, the by‐catch of older dolphins when targeting soles may be due to their co‐occurrence in the same habitats at specific periods. For example, dolphins may follow their preferred prey close to the seabed by following their diel vertical distribution (e.g., vertical movements of anchovy with a descending phase occurring during the day: Tsagarakis et al., 2012, or sardines with a descending phase during the night: Giannoulaki et al., 1999; Zwolinski et al., 2007). These particular foraging behaviors might be more common in adults than in juveniles, explaining such differences in the size of individuals caught. Hake is the only species of interest that is preyed upon by common dolphins; it is possible that this species is preferentially preyed upon by adult individuals or that the strong overlap with the diet of adult individuals (Cabral & Murta, 2002), combined with its more offshore distribution and larger size than coastal fishes, explains that adult individuals are more frequently associated with it. More puzzling are the differences in the size and mass of by‐caught individuals observed when fishing for various demersal fish (see Appendix S1: Section S7 for some plausible explanations).

### Conservation and management implications

The present results have strong implications for conservation. First, the fact that age and sex classes are not equally sensitive to by‐catch may have implications for population dynamics (as observed for albatrosses in Tuck et al., 2015 or hypothesized for common dolphins in Brown et al., 2014). In our particular case, young individuals appeared to be generally more sensitive to by‐catch, except for sexually mature harbor porpoise males, who were also potentially at risk. In both cases, such dynamics could strongly influence the recruitment of the population (by removing individuals before they have reproduced or by reducing the number of potential reproducers) and lead to a declining population (Caley et al., 1996; Horning & Mellish, 2012; Wade et al., 2012). Such a demographic decline was predicted by simulations in harbor porpoise (Booth et al., 2020) and observed in a recent study on common dolphins in the Bay of Biscay (Rouby et al., 2025).

Second, our study highlighted that some practices are likely to pose a significant threat to specific age classes. For example, juveniles of both species appear to be particularly vulnerable to gillnet fishing, while juvenile harbor porpoises are also vulnerable to small mesh sizes and juvenile common dolphins to the targeting of coastal fishes. Such results should encourage consideration of these parameters when fishing in high‐risk environments and periods where juveniles are likely to be present and may argue for the protection of such habitats (Gilman et al., 2023).

#### Finding trade‐offs in data collection and management

This study highlights the need to better consider phenotypic data when collecting by‐catch information. Echoing Gianuca et al. (2017) and ICES (2024b), we recommend that information on sex and age (or at least their proxies, such as size or mass) should be systematically reported when monitoring by‐catch of small cetaceans, ideally with pictures for data validation. In our specific case, these data were systematically collected during the observation programme and stranding monitoring, but information on BM was often missing or not individualized in the declaration dataset, which also lacked information on the size and sex of by‐caught individuals (completely missing). While measuring size may be time‐consuming and may not be adopted by the fishermen, we believe that rapid onboard sex determination should be feasible and should be encouraged (e.g., by training). Furthermore, this study shows that rapid onboard guess estimates may be a good compromise when precise measurements are not feasible or well accepted by the profession. Indeed, models using “guesstimated” BM gave results very close to those obtained using precisely measured body size, for variables present in both types of model (see effects of mesh size in Figure 4, or fishing gear type and fished/targeted taxa in Figure 5). It is possible that the gains in statistical robustness due to the larger sample size obtained from the “guesstimated” declarations compensate for the inaccuracy of the BM estimations. Even if the declarations are incomplete, there are still far more of them than observations (between 2020 and 2023, there were at least twice as many declarations as observations of by‐catch, and in 2022, there were more than five times as many declarations as observations). This does not mean that precise measurements are unnecessary, as they are still paramount to analyze precise processes, such as demographic evolution or estimation of the proportion of sexually or physically mature individuals. Rather, it means that fishermen may have limited time for scientific measurements and may therefore be more inclined to perform rapid, basic estimations sufficient to obtain an initial screening about the at‐risk population segments.

On the other hand, some differences emerged when comparing results on by‐catch sensitivity between datasets. Contrary to stranding data, we did not find any significant imbalance in the sex ratio reported from observation/declaration pooled data. Moreover, unlike stranding data, the median BL of by‐caught harbor porpoises from pooled data did not significantly differ from the asymptotic length of adult individuals. For males, this BL was even larger than that of sexually mature individuals (Figure 1). These differences with stranding data may emerge from the low sample size available from observation/declaration data relative to stranding data, or sex‐ and age‐specific patterns in strandings or in the collection of observation/declaration data (e.g., differences in buoyancy, changes in fishermen's behavior during surveys, under‐reporting of specific events). Therefore, studies disentangling the dynamics of by‐catch risk should also benefit from increased and improved sampling effort. Most current sampling methods, such as those used in this research, have major limitations in data collection. Observer programmes often lack coverage due to the high cost of implementation (Babcock et al., 2003). Some vessels are unable to take observers on board due to a lack of personnel authorization, safety concerns, or insufficient space. This issue particularly affects smaller vessels and can skew sampling. Additionally, fishermen's declarations are often incomplete due to a lack of incentive to report and concerns about negative repercussions for the industry regarding by‐catch issues, as noted in Basran and Sigurðsson (2021). Lastly, stranding data do not include all individuals that sink or drift offshore (Peltier et al., 2012). Additional efforts should be made to improve data completeness: Increased participation of fishermen in catch reporting (Brevé et al., 2024) and the use of onboard cameras (remote electronic monitoring, Pierre et al., 2024; now mandatory in France for boats over 8 m operating in the Bay of Biscay) are probably the most effective and promising solutions.

Another recommendation would be to be as precise as possible about the context in which by‐catches occurred, by providing information on the fishing techniques used and, where possible, the environmental context associated with them. In our particular context, the declaration data are lacking in details, with missing data on the targeted taxa, as well as a general lack of information on the fishing effort and the specificity of the fishing gear (depth, speed) associated with the fishing event. The reporting of such information when by‐catches occur should be encouraged to improve the description of the processes involved. Attention should also be paid to the correlation structure of the data collected in the surveys: The areas, periods, and fleets sampled should be sufficiently diverse to represent the existing diversity of practices (i.e., good stratification of monitoring programmes; see ICES, 2024a, 2024b for best practices in the collection of by‐catch data). Recent ICES reports on observer programme best practices recommend monitoring at least 5% of each fishing fleet's effort (ICES, 2022a). The scope for selection bias is large in both the collected declaration and observation data on small cetacean by‐catch. Implementing a statistically‐driven design (that is, random allocation of observers to fishing trips) instead of leaving it to the discretion of skippers would allow the collection of data that are representative. Currently, representativeness is not guaranteed, and consequently a robust assessment of the causal effects of several variables is not possible.

#### Towards spatiotemporally integrated management

In addition to the importance of fishing techniques in by‐catch risk, we also found that spatiotemporal variables often correlated with different patterns in by‐caught phenotypes (as also observed for example in López et al., 2002). First, we found that, in both species, smaller males were by‐caught in spring (second trimester), with the size of by‐caught males gradually increasing afterwards. The particularly low proportion of sexually mature males by‐caught in spring (Appendix S1: Tables S6 and S7: 17.3% in common dolphins, between 20.6% and 30.5% in harbor porpoises) may reflect the reproductive cycle of the species, with reproduction occurring between April and September for common dolphins (Murphy et al., 2013) and between May and August for harbor porpoises (Lockyer, 2003). Indeed, we may hypothesize that the increase in the capture of sexually immature individuals is related to the segregation of weaned juveniles from breeding females just before or at the beginning of the reproductive season. This may be combined with the high risk‐taking behavior of these inexperienced males. For common dolphins, more females were also by‐caught during autumn (fourth trimester); again this could be related to the reproductive cycle. On the one hand, risk‐taking behavior in adult females may increase during lactation, when rearing young or rebuilding reserves, as observed in bottlenose dolphins, with changes in vigilance and foraging behaviors after birth (Hill et al., 2008; Miketa et al., 2018). On the other hand, juvenile females could also present a higher foraging rate after late infancy, as observed in bottlenose dolphins (Krzyszczyk et al., 2017). Second, we found that larger individuals (and therefore probably older: Appendix S1: Tables S6 and S7) were by‐caught in the southern part of the Bay of Biscay than in the northern one; with also significantly more males in the southernmost part. This could, again, be related to the reproductive habits of both species with a preference for shallower waters (as can be found in the Channel) as juvenile‐specific habitats (Koschinski, 2001; Spyrakos et al., 2011); and plausible spatial age segregation with adult males potentially occupying the southern part of the distribution in a similar vein as the potential segregation between offshore and inshore areas (Murphy et al., 2013). However, this could also be due to differences in the spatial distribution of body size, which is likely in harbor porpoises but not in common dolphins. Indeed, French harbor porpoises are a mixture of the two genetically distinct populations previously identified along the Iberian coasts and in the North Atlantic (Alfonsi et al., 2012). These populations differ in body size, with larger individuals found in the Iberian population (Fontaine et al., 2017). The gradient of admixture levels from north to south may explain the spatial differences in body size observed here, similarly to Murphy et al. (2020). Conversely, in common dolphins, only a slight, reversed latitudinal cline has been identified, with larger males in the northeastern Atlantic compared to those off the northwest coast of Spain (Murphy et al., 2013). On a larger temporal scale, we also found that the size of by‐caught male common dolphins or female harbor porpoises tended to decrease over the years (except for the 2004–2007 period for the first case, and the 2020–2022 period for the second). That suggests an increase in by‐catch of young individuals of these sexes (Appendix S1: Tables S6 and S7), which could be due to a change in the behavior of these age and sex classes or a change in fishing practices with specific age or sex‐related risk patterns. An alternative hypothesis, that remains to be explicitly tested, would be that the proportion of juveniles in the population increased over time due to a depletion of adults and/or an influx of younger ones. This could potentially result from by‐catch dynamics, with fewer individuals reaching adult age over time due to juvenile by‐catch, and with potential demographic compensation through immigration of juveniles from other areas or an increased birth rate. This hypothesis is currently supported by the decline in female longevity observed in the Bay of Biscay, which is probably due to by‐catch, while abundance remains stable (Rouby et al., 2025).

These trends again support the implementation of specific measures during periods and in areas of particular risk, for example by enforcing protected areas or temporary closures during key breeding periods and areas (as also suggested by Tuck et al., 2015; Vishnyakova & Gol'din, 2015), or by promoting a shift in the fishing effort towards the use of fishing gears with a low risk of by‐catch (e.g., Jenkins & Garrison, 2013). These preliminary results also argue for better consideration of the risk landscape that emerges from the global environmental context (Gilman et al., 2023). Here, we did not consider exogenous variables other than spatiotemporal ones, even though some are known to be important for marine mammal by‐catch (Northridge et al., 2017). This choice was partly out of necessity due to missing data and multicollinearity, but we propose some potential study suggestion and promising perspectives in Appendix S1: Section S8 on these topics.

## AUTHOR CONTRIBUTIONS

Mathieu Brevet conceived the ideas and designed methodology; Laurent Dubroca, Hélène Peltier, and Matthieu Authier collected the data; Mathieu Brevet analyzed the data; Mathieu Brevet led the writing of the manuscript. All authors contributed critically to the drafts and gave final approval for publication.

## CONFLICT OF INTEREST STATEMENT

The authors declare no conflicts of interest.

## Supporting information


Appendix S1:

## Data Availability

Code (Brevet, 2026) used to process, analyze the data, and produce the figures and tables of this study is available in Zenodo at https://doi.org/10.5281/zenodo.18337382. The fisheries observer data (OBSMER data) and French officially reported by‐catch data (SACROIS data) used in this study are owned by the French General Directorate of Maritime Affairs, Fisheries and Aquaculture (DGAMPA: https://www.mer.gouv.fr/peche-et-aquaculture; email: dpma@agriculture.gouv.fr) and are subject to access restrictions. These data are considered sensitive under the General Data Protection Regulation (GDPR) of the European Union (Regulation EU 2016/679) and are not publicly available. However, they can be accessed by qualified researchers upon request through the Fishery Information System at IFREMER, via the following online form: https://sih.ifremer.fr/Donnees. The specific data query carried out for this study covers the years 2005–2023 for OBSMER and 2019 to 2023 for SACROIS. The stranding data used in this study are owned by the Observatoire PELAGIS – Réseau National Échouage (National Stranding Network), UAR 3462, CNRS‐La Rochelle University, and are available upon request (https://www.observatoire-pelagis.cnrs.fr/echouages/reseau-national-echouage/; email: pelagis@univ-lr.fr). The specific data query carried out for this study covers the years 2000–2022.

## References

[eap70216-bib-0001] Alfonsi, E. , S. Hassani , F.‐G. Carpentier , J.‐Y. Le Clech , W. Dabin , O. Van Canneyt , M. C. Fontaine , and J.‐L. Jung . 2012. “A European Melting Pot of Harbour Porpoise in the French Atlantic Coasts Inferred from Mitochondrial and Nuclear Data.” PLoS One 7(9): e44425. 10.1371/journal.pone.0044425. 22984507 PMC3440431

[eap70216-bib-0002] Authier, M. , H. Peltier , G. Dorémus , W. Dabin , O. Van Canneyt , and V. Ridoux . 2014. “How Much Are Stranding Records Affected by Variation in Reporting Rates? A Case Study of Small Delphinids in the Bay of Biscay.” Biodiversity and Conservation 23(10): 2591–2612. 10.1007/s10531-014-0741-3.

[eap70216-bib-0003] Babcock, E. , E. Pikitch , and C. Hudson . 2003. How Much Observer Coverage Is Enough to Adequately Estimate Bycatch. Technical report: Rosenstiel School of Marine and Atmospheric Science, University of Miami, October.

[eap70216-bib-0004] Ball, L. , K. Shreves , M. Pilot , and A. E. Moura . 2017. “Temporal and Geographic Patterns of Kinship Structure in Common Dolphins (*Delphinus delphis*) Suggest Site Fidelity and Female‐Biased Long‐Distance Dispersal.” Behavioral Ecology and Sociobiology 71(8): 123. 10.1007/s00265-017-2351-z. 28794579 PMC5522516

[eap70216-bib-0005] Basran, C. J. , and G. M. Sigurðsson . 2021. “Using Case Studies to Investigate Cetacean Bycatch/Interaction Under‐Reporting in Countries with Reporting Legislation.” Frontiers in Marine Science 8: 779066. 10.3389/fmars.2021.779066.

[eap70216-bib-0006] Bjørge, A. , and K. A. Tolley . 2009. “Harbor Porpoise: *Phocoena phocoena* .” In Encyclopedia of Marine Mammals, 2nd ed., edited by W. F. Perrin , B. Würsig , and J. G. M. Thewissen , 530–533. London: Academic Press. 10.1016/B978-0-12-373553-9.00125-5.

[eap70216-bib-0007] Booth, C. G. , R. R. Sinclair , and J. Harwood . 2020. “Methods for Monitoring for the Population Consequences of Disturbance in Marine Mammals: A Review.” Frontiers in Marine Science 7: 115. 10.3389/fmars.2020.00115.

[eap70216-bib-0008] Brennecke, D. , M. Wahlberg , A. Gilles , and U. Siebert . 2021. “Age and Lunar Cycle Predict Harbor Porpoise Bycatch in the South‐Western Baltic Sea.” PeerJ 9: e12284. 10.7717/peerj.12284. 34760359 PMC8556710

[eap70216-bib-0009] Brevé, N. W. P. , K. Urbanovych , A. T. J. Murk , P. A. M. van Zwieten , L. A. J. Nagelkerke , and M. Kraan . 2024. “Fishers' Willingness to Report Incidental Bycatches of Endangered, Threatened and Protected Fish Species: The Case of European Sturgeon in the Northeast Atlantic Ocean.” Marine Policy 162: 106056. 10.1016/j.marpol.2024.106056.

[eap70216-bib-0010] Brevet, M. 2026. “Mathieu‐Brevet/Linking‐by‐Caught‐Cetacean‐Traits‐to‐Fishing‐Techniques‐Insights‐from‐Two‐Species‐of‐Small‐Cetaceans: Data Curation and Analysis Code Associated with the Manuscript.” Linking by‐Caught Cetacean Traits to Fishing Techniques: Insights from Two Species of Small Cetaceans after Acceptance in Ecological Application. (V.1.1.1). Zenodo. 10.5281/zenodo.18337382 41887714

[eap70216-bib-0011] Brophy, J. , S. Murphy , and E. Rogan . 2009. “The Diet and Feeding Ecology of the Common Dolphin (*Delphinus delphis*) in the Northeast Atlantic.” Technical report SC/61/SM14. International Whaling Commission, January.

[eap70216-bib-0012] Brown, S. , D. Reid , and E. Rogan . 2013. “A Risk‐Based Approach to Rapidly Screen Vulnerability of Cetaceans to Impacts from Fisheries Bycatch.” Biological Conservation 168: 78–87. 10.1016/j.biocon.2013.09.019.

[eap70216-bib-0013] Brown, S. , D. Reid , and E. Rogan . 2014. “Characteristics of Fishing Operations, Environment and Life History Contributing to Small Cetacean Bycatch in the Northeast Atlantic.” PLoS One 9(8): e104468. 10.1371/journal.pone.0104468. 25121802 PMC4133181

[eap70216-bib-0014] Byrd, B. L. , and A. A. Hohn . 2017. “Differential Risk of Bottlenose Dolphin (*Tursiops truncatus*) Bycatch in North Carolina, USA.” Aquatic Mammals 43(5): 558–569. issn: 01675427. 10.1578/AM.43.5.2017.558.

[eap70216-bib-0015] Cabral, H. N. , and A. G. Murta . 2002. “The Diet of Blue Whiting, Hake, Horse Mackerel and Mackerel off Portugal.” Journal of Applied Ichthyology 18(1): 14–23. issn: 1439‐0426. 10.1046/j.1439-0426.2002.00297.x.

[eap70216-bib-0016] Caley, M. J. , M. H. Carr , M. A. Hixon , T. P. Hughes , G. P. Jones , and B. A. Menge . 1996. “Recruitment and the Local Dynamics of Open Marine Populations.” Annual Review of Ecology, Evolution, and Systematics 27(1): 477–500. 10.1146/annurev.ecolsys.27.1.477.

[eap70216-bib-0017] Cañadas, A. , and P. S. Hammond . 2008. “Abundance and Habitat Preferences of the Short‐Beaked Common Dolphin *Delphinus delphis* in the Southwestern Mediterranean: Implications for Conservation.” Endangered Species Research 4: 309–331. 10.3354/esr00073.

[eap70216-bib-0018] Canneyt, V. , W. D. Olivier , D. Cécile , G. Dorémus , G. Laurence , V. Ridoux , and J. Spitz . 2015. “Guide Des Échouages de Mammifères Marins.” 10.13140/RG.2.1.1495.6002.

[eap70216-bib-0019] Carlström, J. , P. Berggren , and N. J. C. Tregenza . 2009. “Spatial and Temporal Impact of Pingers on Porpoises.” Canadian Journal of Fisheries and Aquatic Sciences 66(1): 72–82. 10.1139/F08-186.

[eap70216-bib-0020] Carretta, J. 2021. “Estimates of Marine Mammal, Sea Turtle, and Seabird Bycatch in the California Large‐Mesh Drift Gillnet Fishery: 1990‐2019.” Technical Report NOAA‐TM‐NMFS‐SWFSC 654. NOAA. 10.25923/7EMJ-ZA90

[eap70216-bib-0021] Carretta, J. , and J. Barlow . 2011. “Long‐Term Effectiveness, Failure Rates, and ‘Dinner Bell’ Properties of Acoustic Pingers in a Gillnet Fishery.” Marine Technology Society Journal 45(5): 7–19. 10.4031/MTSJ.45.5.3.

[eap70216-bib-0022] Castro, J. , A. Couto , F. O. Borges , A. Cid , M. I. Laborde , H. C. Pearson , and R. Rosa . 2020. “Oceanographic Determinants of the Abundance of Common Dolphins (*Delphinus delphis*) in the South of Portugal.” Oceans 1(3): 165–173. 10.3390/oceans1030012.

[eap70216-bib-0023] Castro, J. , C. Faustino , A. Cid , A. Quirin , F. L. Matos , R. Rosa , and H. C. Pearson . 2022. “Common Dolphin (*Delphinus delphis*) Fission–Fusion Dynamics in the South Coast of Portugal.” Behavioral Ecology and Sociobiology 76(9): 128. 10.1007/s00265-022-03235-0.

[eap70216-bib-0024] Caswell, H. , S. Brault , A. J. Read , and T. D. Smith . 1998. “Harbor Porpoise and Fisheries: An Uncertainty Analysis of Incidental Mortality.” Ecological Applications 8(4): 1226–1238. 10.1890/1051-0761(1998)008[1226:HPAFAU]2.0.CO;2.

[eap70216-bib-0025] Certain, G. , J. Masse , O. Canneyt , P. Petitgas , G. Dorémus , and V. Ridoux . 2011. “Investigating the Coupling between Small Pelagic Fish and Marine Top Predators Using Data Collected from Ecosystem‐Based Surveys.” Marine Ecology Progress Series 422: 23–39. 10.3354/meps08932.

[eap70216-bib-0026] Certain, G. , V. Ridoux , O. van Canneyt , and V. Bretagnolle . 2008. “Delphinid Spatial Distribution and Abundance Estimates over the Shelf of the Bay of Biscay.” ICES Journal of Marine Science 65(4): 656–666. 10.1093/icesjms/fsn046.

[eap70216-bib-0027] Clay, T. A. , C. Small , G. N. Tuck , D. Pardo , A. P. B. Carneiro , A. G. Wood , J. P. Croxall , G. T. Crossin , and R. A. Phillips . 2019. “A Comprehensive Large‐Scale Assessment of Fisheries Bycatch Risk to Threatened Seabird Populations.” Journal of Applied Ecology 56(8): 1882–1893. 10.1111/1365-2664.13407.

[eap70216-bib-0028] Cloâtre, T. , M. Authier , M. Brevet , L. Dubroca , and S. Demaneche . 2023. “Analyse des données de captures accidentelles de petits cétacés dans le Golfe de Gascogne en période hivernale et préparation de la campagne 2023‐2024.” Technical Report. DGAMPA – Direction Générale des Affaires Maritimes, de la Pêche et de lAquaculture, DEB – Direction de lEau et de la Biodiversité.

[eap70216-bib-0029] Cloâtre, T. , M. Scavinner , J. Sagan , L. Dubroca , N. Billet , J. Sagan , L. Dubroca , and N. Billet . 2022. “Captures et Rejets Des Métiers de Pêche Français.” Résultats Des Observations à Bord Des Navires de Pêche Professionnelle En 2020. ObsMer. Technical report. IFREMER. 10.13155/88406.

[eap70216-bib-0132] Collet, A. 1981. Biologie du Dauphin Commun Delphinus Delphis L. en Atlantique Nord‐Est 156. Doctoral thesis presented to the University of Poitiers.

[eap70216-bib-0030] Cox, T. M. , A. J. Read , A. Solow , and N. Tregenza . 2001. “Will Harbour Porpoises (*Phocoena phocoena*) Habituate to Pingers?” Journal of Cetacean Research and Management 3(1): 81–86. 10.47536/jcrm.v3i1.904.

[eap70216-bib-0031] Dawson, S. M. , S. M. Dawson , S. Northridge , D. Waples , and A. J. Read . 2013. “To Ping or Not to Ping: The Use of Active Acoustic Devices in Mitigating Interactions between Small Cetaceans and Gillnet Fisheries.” Endangered Species Research 19(3): 201–221. 10.3354/esr00464.

[eap70216-bib-0032] Dawson, S. M. , A. Read , and E. Slooten . 1998. “Pingers, Porpoises and Power: Uncertainties with Using Pingers to Reduce Bycatch of Small Cetaceans.” Biological Conservation 84(2): 141–146. 10.1016/S0006-3207(97)00127-4.

[eap70216-bib-0033] De Boer, M. , J. Saulino , M. Leopold , P. Reijnders , and M. Simmonds . 2012. “Interactions between Short‐Beaked Common Dolphin (*Delphinus delphis*) and the Winter Pelagic Pair‐Trawl Fishery off Southwest England (UK).” International Journal of Biodiversity and Conservation 4: 481–499. 10.5897/IJBC12.016.

[eap70216-bib-0034] Fernández‐Contreras, M. M. , L. Cardona , C. H. Lockyer , and A. Aguilar . 2010. “Incidental Bycatch of Short‐Beaked Common Dolphins (*Delphinus delphis*) by Pairtrawlers off Northwestern Spain.” ICES Journal of Marine Science 67(8): 1732–1738. 10.1093/icesjms/fsq077.

[eap70216-bib-0035] Fertl, D. , and S. Leatherwood . 1997. “Cetacean Interactions with Trawls: A Preliminary Review.” Journal of Northwest Atlantic Fishery Science 22: 219–248. 10.2960/J.v22.a17.

[eap70216-bib-0036] Fontaine, M. C. , O. Thatcher , N. Ray , S. Piry , A. Brownlow , N. J. Davison , P. Jepson , R. Deaville , and S. J. Goodman . 2017. “Mixing of Porpoise Ecotypes in Southwestern UK Waters Revealed by Genetic Profiling.” Royal Society Open Science 4(3): 160992. 10.1098/rsos.160992. 28405389 PMC5383846

[eap70216-bib-0037] Fox, J. , and S. Weisberg . 2019. An R Companion to Applied Regression. Thousand Oaks, CA: SAGE Publications.

[eap70216-bib-0038] Fruet, P. F. , P. G. Kinas , K. G. da Silva , J. C. Di Tullio , D. S. Monteiro , L. Dalla Rosa , S. C. Estima , and E. R. Secchi . 2012. “Temporal Trends in Mortality and Effects of by‐Catch on Common Bottlenose Dolphins, *Tursiops truncatus*, in Southern Brazil.” Journal of the Marine Biological Association of the United Kingdom 92(8): 1865–1876. 10.1017/S0025315410001888.

[eap70216-bib-0039] Giannoulaki, M. , A. Machias , and N. Tsimenides . 1999. “Ambient Luminance and Vertical Migration of the Sardine *Sardina pilchardus* .” Marine Ecology Progress Series 178: 29–38. 10.3354/meps178029.

[eap70216-bib-0040] Gianuca, D. , R. A. Phillips , S. Townley , and S. C. Votier . 2017. “Global Patterns of Sex‐ and Age‐Specific Variation in Seabird Bycatch.” Biological Conservation 205: 60–76. 10.1016/j.biocon.2016.11.028.

[eap70216-bib-0041] Gilles, A. , M. Authier , N. R. Martinez , H. Araújo , A. Blanchard , J. Carlström , C. Eira , et al. 2023. “Estimates of Cetacean Abundance in European Atlantic Waters in Summer 2022 from the SCANS‐IV Aerial and Shipboard Surveys.” Technical repOrt. SCANS‐IV Surveys, September. 10.13140/RG.2.2.34873.95845

[eap70216-bib-0042] Gilman, E. , M. Chaloupka , H. Booth , M. Hall , H. Murua , and J. Wilson . 2023. “Bycatch‐Neutral Fisheries through a Sequential Mitigation Hierarchy.” Marine Policy 150: 105522. 10.1016/j.marpol.2023.105522.

[eap70216-bib-0043] Gilman, E. , M. Chaloupka , J. Peschon , and S. Ellgen . 2016. “Risk Factors for Seabird Bycatch in a Pelagic Longline Tuna Fishery.” PLoS One 11(5): e0155477. 10.1371/journal.pone.0155477. 27192492 PMC4871550

[eap70216-bib-0044] Giménez, J. , M. Authier , J. Valeiras , E. Abad , A. Marçalo , M. Coll , P. Gauffier , M. B. Santos , and R. de Stephanis . 2021. “Consumption Rates and Interaction with Fisheries of Mediterranean Common Dolphins in the Alboran Sea.” Regional Studies in Marine Science 45: 101826. 10.1016/j.rsma.2021.101826.

[eap70216-bib-0045] Giralt Paradell, O. , E. Rogan , and M. Jessopp . 2024. “The Seasonal Distribution and Abundance of Seabirds, Cetaceans and Other Megafauna off the South and Southwest Irish Coast.” Technical report. Department of the Environment, Climate & Communications and Department of Housing, Local Government & Heritage, Ireland. https://assets.gov.ie/static/documents/the-seasonal-distribution-and-abundance-ofseabirds-cetaceans-and-other-megafauna-off-.pdf

[eap70216-bib-0046] Gonzalvo, J. , and P. Carpentieri . 2023. Depredation by Marine Mammals in Fishing Gear. Rome: FAO.

[eap70216-bib-0047] Hall, M. A. 1996. “On Bycatches.” Reviews in Fish Biology and Fisheries 6(3): 319–352. 10.1007/BF00122585.

[eap70216-bib-0048] Harder, J. H. , H. M. Hill , K. M. Dudzinski , K. T. Sanabria , S. Guarino , and S. A. Kuczaj . 2016. “The Development of Echolocation in Bottlenose Dolphins.” International Journal of Comparative Psychology 29(1): 1–19. 10.46867/ijcp.2016.29.00.17.

[eap70216-bib-0049] Hartig, F. 2022. “DHARMa: Residual Diagnostics for Hierarchical (Multi‐Level/Mixed) Regression Models.” R Package Version 0.4.6. https://CRAN.R-project.org/package=DHARMa

[eap70216-bib-0050] Heswall, A. M. , M. R. Friesen , A. L. Brunton Martin , and A. C. Gaskett . 2021. “Seabird Bycatch Risk Correlates with Body Size, and Relatively Larger Skulls, Bills, Wings and Sensory Structures.” Marine Biology 168(5): 70. 10.1007/s00227-021-03873-4.

[eap70216-bib-0051] Hill, H. M. , D. A. Carder , and S. H. Ridgway . 2008. “Vigilance in Female Bottlenose Dolphins (*Tursiops* sp.) before and after Calving.” International Journal of Comparative Psychology 21(1): 37–57. 10.46867/ijcp.2008.21.01.01.

[eap70216-bib-0052] Hines, E. , L. S. Ponnampalam , C. Junchompoo , C. Peter , L. Vu , T. Huynh , M. Caillat , et al. 2020. “Getting to the Bottom of Bycatch: A GIS‐Based Toolbox to Assess the Risk of Marine Mammal Bycatch.” Endangered Species Research 42: 37–57. 10.3354/esr01037.

[eap70216-bib-0053] Horning, M. , and J.‐A. E. Mellish . 2012. “Predation on an Upper Trophic Marine Predator, the Steller Sea Lion: Evaluating High Juvenile Mortality in a Density Dependent Conceptual Framework.” PLoS One 7(1): e30173. 10.1371/journal.pone.0030173. 22272296 PMC3260237

[eap70216-bib-0054] IAMMWG , C. J. Camphuysen , and M. L. Siemensma . 2015. “A Conservation Literature Review for the Harbour Porpoise (*Phocoena ohocoena*).” Technical Report 566. Joint Nature Conservation Committee.

[eap70216-bib-0055] ICES . 2019. “Working Group on Bycatch of Protected Species (WGBYC).” Technical Report. ICES Scientific Reports, January. 10.17895/ices.pub.5563.

[eap70216-bib-0056] ICES . 2020. “Workshop on Fisheries Emergency Measures to Minimize BYCatch of Short‐Beaked Common Dolphins in the Bay of Biscay and Harbour Porpoise in the Baltic Sea (WKEMBYC).” Technical report. ICES Scientific Reports, January. 10.17895/ices.pub.7472.

[eap70216-bib-0057] ICES . 2022a. “External Report on the Review of Monitoring PETS Bycatch of Mammals, Birds, Turtles and Fish for ICES under the Service of EC DG Environment.” Technical Report. ICES Scientific Reports, February 25, 2022. 10.17895/ices.pub.10075.

[eap70216-bib-0058] ICES . 2022b. “Working Group on Bycatch of Protected Species (WGBYC).” Technical Report. ICES Scientific Reports, December 9, 2022. 10.17895/ices.pub.21602322.v1

[eap70216-bib-0059] ICES . 2023. “Working Group on Bycatch of Protected Species (WGBYC).” Technical Report. ICES Scientific Reports, December 1, 2023. 10.17895/ices.pub.24659484.v3

[eap70216-bib-0060] ICES . 2024a. “Second Workshop on Appropriate Sampling Schemes for Protected Endangered and Threatened Species Bycatch (WKPETSAMP2).” Technical Report. ICES Scientific Reports, April 8, 2024. 10.17895/ices.pub.25061459.v2

[eap70216-bib-0061] ICES . 2024b. “Third Workshop on Appropriate Sampling Schemes for Protected Endangered and Threatened Species Bycatch (WKPETSAMP3).” Technical Report. ICES Scientific Reports, April 8, 2024. 10.17895/ices.pub.25061522.v2.

[eap70216-bib-0062] IMR/NAMMCO . 2019. “Report of Joint IMR/NAMMCO International Workshop on the Status of Harbour Porpoises in the North Atlantic.” Technical Report. North Atlantic Marine Mammal Commission and the Norwegian Institute of Marine Research.

[eap70216-bib-0063] Jefferson, T. A. , and B. E. Curry . 1994. “A Global Review of Porpoise (*Cetacea*: *Phocoenidae*) Mortality in Gillnets.” Biological Conservation 67(2): 167–183. 10.1016/0006-3207(94)90363-8.

[eap70216-bib-0064] Jenkins, L. D. , and K. Garrison . 2013. “Fishing Gear Substitution to Reduce Bycatch and Habitat Impacts: An Example of Social–Ecological Research to Inform Policy.” Marine Policy 38: 293–303. 10.1016/j.marpol.2012.06.005.

[eap70216-bib-0065] Jones, A. A. , N. G. Hall , and I. C. Potter . 2010. “Species Compositions of Elasmobranchs Caught by Three Different Commercial Fishing Methods off Southwestern Australia, and Biological Data for Four Abundant Bycatch Species.” Fishery Bulletin 108(4): 365–382.

[eap70216-bib-0135] Karakosta, C. V. , P. D. Jepson , H. Ohira , A. Moore , P. M. Bennett , and W. V. Holt . 1999. “Testicular and Ovarian Development in the Harbor Porpoise (*Phocoena Phocoena*).” Journal of Zoology 249: 111–121. 10.1111/j.1469-7998.1999.tb01064.x.

[eap70216-bib-0066] Kindt‐Larsen, L. , G. Glemarec , C. W. Berg , S. Königson , A.‐M. Kroner , M. Søgaard , and D. Lusseau . 2023. “Knowing the Fishery to Know the Bycatch: Bias‐Corrected Estimates of Harbour Porpoise Bycatch in Gillnet Fisheries.” Proceedings of the Royal Society B: Biological Sciences 290(2002): 20222570. 10.1098/rspb.2022.2570. PMC10336385 37434528

[eap70216-bib-0067] Komoroske, L. M. , and R. L. Lewison . 2015. “Addressing Fisheries Bycatch in a Changing World.” Frontiers in Marine Science 2: 83. 10.3389/fmars.2015.00083.

[eap70216-bib-0068] Koschinski, S. 2001. “Current Knowledge on Harbour Porpoises (*Phocoena phocoena*) in the Baltic Sea.” Ophelia 55(3): 167–197. 10.1080/00785326.2001.10409483.

[eap70216-bib-0069] Krzyszczyk, E. , E. M. Patterson , M. A. Stanton , and J. Mann . 2017. “The Transition to Independence: Sex Differences in Social and Behavioural Development of Wild Bottlenose Dolphins.” Animal Behaviour 129: 43–59. 10.1016/j.anbehav.2017.04.011.

[eap70216-bib-0070] Kuczaj, S. A., II , D. Yeater , and L. Highfill . 2012. “How Selective Is Social Learning in Dolphins?” International Journal of Comparative Psychology 25(3): 221–236. 10.46867/ijcp.2012.25.03.02.

[eap70216-bib-0071] Lambert, C. , M. Authier , M. Doray , G. Dorémus , J. Spitz , and V. Ridoux . 2018. “Decadal Stability in Top Predator Habitat Preferences in the Bay of Biscay.” Progress in Oceanography 166: 109–120. 10.1016/j.pocean.2018.03.007.

[eap70216-bib-0072] Lambert, C. , E. Pettex , G. Dorémus , S. Laran , E. Stéphan , O. Van Canneyt , and V. Ridoux . 2017. “How Does Ocean Seasonality Drive Habitat Preferences of Highly Mobile Top Predators? Part II: The Eastern North‐Atlantic.” Deep‐Sea Research. Part II, Topical Studies in Oceanography 141: 133–154. 10.1016/j.dsr2.2016.06.011.

[eap70216-bib-0073] Laran, S. , M. Authier , A. Blanck , G. Doremus , H. Falchetto , P. Monestiez , E. Pettex , E. Stephan , O. Van Canneyt , and V. Ridoux . 2017. “Seasonal Distribution and Abundance of Cetaceans within French Waters‐ Part II: The Bay of Biscay and the English Channel.” Deep‐Sea Research. Part II, Topical Studies in Oceanography 141: 31–40. 10.1016/j.dsr2.2016.12.012.

[eap70216-bib-0074] Lauriano, G. , L. Caramanna , M. Scarnò , and F. Andaloro . 2009. “An Overview of Dolphin Depredation in Italian Artisanal Fisheries.” Journal of the Marine Biological Association of the United Kingdom 89(5): 921–929. 10.1017/S0025315409000393.

[eap70216-bib-0075] Leblond, E. , F. Daures , P. Berthou , and C. Dintheer . 2008. “The Fisheries Information System of Ifremer: A Multidisciplinary Monitoring Network and an Integrated Approach for the Assessment of French Fisheries, Including Small‐Scale Fisheries.” In ICES 2008 Annual Science Conference

[eap70216-bib-0076] Lenth, R. V. 2024. “emmeans: Estimated Marginal Means, aka Least‐Squares Means.” R Package Version 1.10.3–090003. https://rvlenth.github.io/emmeans/

[eap70216-bib-0077] Lewison, R. L. , L. B. Crowder , A. J. Read , and S. A. Freeman . 2004. “Understanding Impacts of Fisheries Bycatch on Marine Megafauna.” Trends in Ecology & Evolution 19(11): 598–604. 10.1016/j.tree.2004.09.004.

[eap70216-bib-0133] Lockyer, C. 1995a. “Investigation of Aspects of the Life History of the Harbor Porpoise, Phocoena Phocoena, in British Waters.” Report of the International Whaling Commission. (Special Issue 16): 189–197.

[eap70216-bib-0134] Lockyer, C. 1995b. “Aspects of the Biology of the Harbor Porpoise, Phocoena Phocoena, from British Waters.” In Whales, Seals, Fish and Man, edited by Blix, A. S. , Walløe, L. and Ulltang, Ø. , 443–457. Amsterdam: Elsevier Science.

[eap70216-bib-0078] Lockyer, C. 2003. “Harbour Porpoises (*Phocoena phocoena*) in the North Atlantic: Biological Parameters.” NAMMCO Scientific Publications 5: 71–89. 10.7557/3.2740.

[eap70216-bib-0136] Lockyer, C. , and C. Kinze . 2003. “Status and Life History of Harbor Porpoise (*Phocoena Phocoena*) in Danish Waters.” NAMMCO Scientific Publications 5: 143–176.

[eap70216-bib-0079] López, A. , M. B. Santos , G. J. Pierce , A. F. González , X. Valeiras , and A. Guerra . 2002. “Trends in Strandings and by‐Catch of Marine Mammals in North‐West Spain during the 1990s.” Journal of the Marine Biological Association of the United Kingdom 82(3): 513–521. 10.1017/S0025315402005805.

[eap70216-bib-0080] Lüdecke, D. 2018. “Ggeffects: Tidy Data Frames of Marginal Effects from Regression Models.” Journal of Open Source Software 3(26): 772. 10.21105/joss.00772.

[eap70216-bib-0081] Maeda, S. , K. Sakurai , T. Akamatsu , A. Matsuda , O. Yamamura , M. Kobayashi , and T. F. Matsuishi . 2021. “Foraging Activity of Harbour Porpoises around a Bottom‐Gillnet in a Coastal Fishing Ground, under the Risk of Bycatch.” PLoS One 16(2): e0246838. 10.1371/journal.pone.0246838. 33571306 PMC7877735

[eap70216-bib-0082] Mannocci, L. , Y. Baidai , F. Forget , M. T. Tolotti , L. Dagorn , and M. Capello . 2021. “Machine Learning to Detect Bycatch Risk: Novel Application to Echosounder Buoys Data in Tuna Purse Seine Fisheries.” Biological Conservation 255: 109004. 10.1016/j.biocon.2021.109004.

[eap70216-bib-0083] Mannocci, L. , W. Dabin , E. Augeraud‐Véron , J.‐F. Dupuy , C. Barbraud , and V. Ridoux . 2012. “Assessing the Impact of Bycatch on Dolphin Populations: The Case of the Common Dolphin in the Eastern North Atlantic.” PLoS One 7(2): e32615. 10.1371/journal.pone.0032615. 22393423 PMC3290591

[eap70216-bib-0084] Marçalo, A. , J. Giménez , L. Nicolau , J. Frois , M. Ferreira , M. Sequeira , C. Eira , G. J. Pierce , and J. Vingada . 2021. “Stranding Patterns and Feeding Ecology of Striped Dolphins, *Stenella coeruleoalba*, in Western Iberia (1981–2014).” Journal of Sea Research 169: 101996. 10.1016/j.seares.2021.101996.

[eap70216-bib-0085] McAuley, R. B. , C. A. Simpfendorfer , and I. W. Wright . 2007. “Gillnet Mesh Selectivity of the Sandbar Shark (*Carcharhinus plumbeus*): Implications for Fisheries Management.” ICES Journal of Marine Science 64(9): 1702–1709. 10.1093/icesjms/fsm136.

[eap70216-bib-0086] McGovern, B. , R. M. Culloch , M. O'Connell , and S. Berrow . 2018. “Temporal and Spatial Trends in Stranding Records of Cetaceans on the Irish Coast, 2002–2014.” Journal of the Marine Biological Association of the United Kingdom 98(5): 977–989. 10.1017/S0025315416001594.

[eap70216-bib-0087] Meager, J. J. , and W. D. Sumpton . 2016. “Bycatch and Strandings Programs as Ecological Indicators for Data‐Limited Cetaceans.” Ecological Indicators 60: 987–995. 10.1016/j.ecolind.2015.08.052.

[eap70216-bib-0088] Meynier, L. , C. Pusineri , J. Spitz , M. B. Santos , G. J. Pierce , and V. Ridoux . 2008. “Intraspecific Dietary Variation in the Short‐Beaked Common Dolphin *Delphinus delphis* in the Bay of Biscay: Importance of Fat Fish.” Marine Ecology Progress Series 354: 277–287. 10.3354/meps07246.

[eap70216-bib-0089] Miketa, M. L. , E. M. Patterson , E. Krzyszczyk , V. Foroughirad , and J. Mann . 2018. “Calf Age and Sex Affect Maternal Diving Behaviour in Shark Bay Bottlenose Dolphins.” Animal Behaviour 137: 107–117. 10.1016/j.anbehav.2017.12.023.

[eap70216-bib-0090] Milani, C. B. , A. Vella , P. Vidoris , A. Christidis , N. Kamidis , and E. Leykaditou . 2019. “Interactions between Fisheries and Cetaceans in the Thracian Sea (Greece) and Management Proposals.” Fisheries Management and Ecology 26(4): 374–388. 10.1111/fme.12370.

[eap70216-bib-0091] Moore, J. E. , and A. J. Read . 2008. “A Bayesian Uncertainty Analysis of Cetacean Demography and Bycatch Mortality Using Age‐at‐Death Data.” Ecological Applications 18(8): 1914–1931. issn: 1939‐5582. 10.1890/07-0862.1. 19263888

[eap70216-bib-0092] Moyes, F. , S. Smout , L. Thomas , A. Kingston , and S. Northridge . 2025. “Factors Associated with Bycatch of Marine Mammals in United Kingdom Static Net Fisheries.” ICES Journal of Marine Science 82(8): 1–16. 10.1093/icesjms/fsaf146.

[eap70216-bib-0093] Murphy, S. , A. Collet , and E. Rogan . 2005. “Mating Strategy in the Male Common Dolphin (*Delphinus delphis*): What Gonadal Analysis Tells Us.” Journal of Mammalogy 86(6): 1247–1258. 10.1644/1545-1542(2005)86[1247:MSITMC]2.0.CO;2.

[eap70216-bib-0094] Murphy, S. , M. A. C. Petitguyot , P. D. Jepson , R. Deaville , C. Lockyer , J. Barnett , M. Perkins , R. Penrose , N. J. Davison , and C. Minto . 2020. “Spatio‐Temporal Variability of Harbor Porpoise Life History Parameters in the North‐East Atlantic.” Frontiers in Marine Science 7: 502352. 10.3389/fmars.2020.502352.

[eap70216-bib-0095] Murphy, S. , E. Pinn , and P. D. Jepson . 2013. “The Short‐Beaked Common Dolphin (*Delphinus Delphis*) in the North‐East Atlantic: Distribution, Ecology, Management and Conservation Status.” In Oceanography and Marine Biology, 1st ed., edited by R. N. Hughes , D. J. Hughes , and I. P. Smith , 201–288. Boca Raton, FL: CRC Press. 10.1201/b15406-5.

[eap70216-bib-0096] Murphy, S. , and E. Rogan . 2006. “External Morphology of the Short‐Beaked Common Dolphin, *Delphinus delphis*: Growth, Allometric Relationships and Sexual Dimorphism.” Acta Zoologica 87(4): 315–329. 10.1111/j.1463-6395.2006.00245.x.

[eap70216-bib-0097] Murphy, S. , A. Winship , W. Dabin , P. D. Jepson , R. Deaville , R. J. Reid , C. Spurrier , et al. 2009. “Importance of Biological Parameters in Assessing the Status of *Delphinus delphis* .” Marine Ecology Progress Series 388: 273–291. 10.3354/meps08129.

[eap70216-bib-0098] Nagelkerke, N. J. D. 1991. “A Note on a General Definition of the Coefficient of Determination.” Biometrika 78(3): 691–692. 10.1093/biomet/78.3.691.

[eap70216-bib-0099] Northridge, S. , A. Coram , A. Kingston , and R. Crawford . 2017. “Disentangling the Causes of Protected‐Species Bycatch in Gillnet Fisheries.” Conservation Biology 31(3): 686–695. 10.1111/cobi.12741. 27109749

[eap70216-bib-0100] Peltier, H. , M. Authier , F. Caurant , W. Dabin , P. Daniel , C. Dars , F. Demaret , et al. 2021. “In the Wrong Place at the Wrong Time: Identifying Spatiotemporal Co‐Occurrence of Bycaught Common Dolphins and Fisheries in the Bay of Biscay (NE Atlantic) from 2010 to 2019.” Frontiers in Marine Science 8: 617342. 10.3389/fmars.2021.617342.

[eap70216-bib-0101] Peltier, H. , M. Authier , W. Dabin , C. Dars , F. Demaret , G. Doremus , O. Van Canneyt , et al. 2020. “Can Modelling the Drift of Bycaught Dolphin Stranded Carcasses Help Identify Involved Fisheries? An Exploratory Study.” Global Ecology and Conservation 21: e00843. 10.1016/j.gecco.2019.e00843.

[eap70216-bib-0102] Peltier, H. , M. Authier , R. Deaville , W. Dabin , P. D. Jepson , O. van Canneyt , P. Daniel , and V. Ridoux . 2016. “Small Cetacean Bycatch as Estimated from Stranding Schemes: The Common Dolphin Case in the Northeast Atlantic.” Environmental Science & Policy 63: 7–18. 10.1016/j.envsci.2016.05.004.

[eap70216-bib-0103] Peltier, H. , W. Dabin , P. Daniel , O. Van Canneyt , G. Dorémus , M. Huon , and V. Ridoux . 2012. “The Significance of Stranding Data as Indicators of Cetacean Populations at Sea: Modelling the Drift of Cetacean Carcasses.” Ecological Indicators 18: 278–290. 10.1016/j.ecolind.2011.11.014.

[eap70216-bib-0104] Perrin, W. F. , and S. B. Reilly . 1984. “Reproductive Parameters of Dolphins and Small Whales of the Family Delphinidae.” In Reproduction in Whales, Dolphins and Porpoises, Vol. 6, edited by W. F. Perrin , L. Brownell, Jr. , and D. P. DeMartin , 97–133. Cambridge, UK: Report of the International Whaling Commission.

[eap70216-bib-0105] Pierre, J. P. , A. Dunn , A. Snedeker , M. Wealti , A. Cozza , and K. Carovano . 2024. “Optimising the Review of Electronic Monitoring Information for Management of Commercial Fisheries.” Reviews in Fish Biology and Fisheries 34(4): 1707–1732. 10.1007/s11160-024-09895-7. 39469569 PMC11512928

[eap70216-bib-0106] Puente, E. , L. Citores , E. Cuende , I. Krug , and M. Basterretxea . 2023. “Bycatch of Short‐Beaked Common Dolphin (*Delphinus delphis*) in the Pair Bottom Trawl Fishery of the Bay of Biscay and Its Mitigation with an Active Acoustic Deterrent Device (Pinger).” Fisheries Research 267: 106819. 10.1016/j.fishres.2023.106819.

[eap70216-bib-0131] R Development Core Team . 2008. R: A Language and Environment for Statistical Computing. Vienna: R Foundation for Statistical Computing. http://www.R-project.org.

[eap70216-bib-0107] Read, A. J. 2008. “The Looming Crisis: Interactions between Marine Mammals and Fisheries.” Journal of Mammalogy 89(3): 541–548. 10.1644/07-MAMM-S-315R1.1.

[eap70216-bib-0108] Read, A. J. , D. M. Waples , K. W. Urian , and D. Swanner . 2003. “Fine‐Scale Behaviour of Bottlenose Dolphins around Gillnets.” Proceedings of the Royal Society of London. Series B, Biological Sciences 270: S90–S92. 10.1098/rsbl.2003.0021. PMC1698016 12952646

[eap70216-bib-0109] Rouby, E. , L. Dubroca , T. Cloâtre , S. Demanèche , M. Genu , K. Macleod , H. Peltier , V. Ridoux , and M. Authier . 2022. “Estimating Bycatch from Non‐representative Samples (II): A Case Study of Pair Trawlers and Common Dolphins in the Bay of Biscay.” Frontiers in Marine Science 8: 795942. 10.3389/fmars.2021.795942.

[eap70216-bib-0110] Rouby, E. , F. Plard , V. Ridoux , A. Mauchamp , W. Dabin , J. Spitz , and M. Authier . 2025. “Longevity Collapse in Dolphins: A Growing Conservation Concern in the Bay of Biscay.” Conservation Letters 18(5): e13142. 10.1111/conl.13142.

[eap70216-bib-0111] Santana‐Garcon, J. , C. B. Wakefield , S. R. Dorman , A. Denham , S. Blight , B. W. Molony , and S. J. Newman . 2018. “Risk Versus Reward: Interactions, Depredation Rates, and Bycatch Mitigation of Dolphins in Demersal Fish Trawls.” Canadian Journal of Fisheries and Aquatic Sciences 75(12): 2233–2240. 10.1139/cjfas-2017-0203.

[eap70216-bib-0112] Santos, M. B. , I. German , D. Correia , F. L. Read , J. M. Cedeira , M. Caldas , A. López , F. Velasco , and G. J. Pierce . 2013. “Long‐Term Variation in Common Dolphin Diet in Relation to Prey Abundance.” Marine Ecology Progress Series 481: 249–268. 10.3354/meps10233.

[eap70216-bib-0113] Santos, M. B. , and G. Pierce . 2003. “The Diet of Harbour Porpoise (*Phocoena phocoena*) in the Northeast Atlantic.” Oceanography and Marine Biology: An Annual Review 41(January): 355–390.

[eap70216-bib-0114] Soykan, C. U. , J. E. Moore , R. Zydelis , L. B. Crowder , C. Safina , and R. L. Lewison . 2008. “Why Study Bycatch? An Introduction to the Theme Section on Fisheries Bycatch.” Endangered Species Research 5(2‐3): 91–102. 10.3354/esr00175.

[eap70216-bib-0115] Spitz, J. , T. Chouvelon , M. Cardinaud , C. Kostecki , and P. Lorance . 2013. “Prey Preferences of Adult Sea Bass *Dicentrarchus labrax* in the Northeastern Atlantic: Implications for Bycatch of Common Dolphin *Delphinus delphis* .” ICES Journal of Marine Science 70(2): 452–461. 10.1093/icesjms/fss200.

[eap70216-bib-0116] Spitz, J. , E. Mourocq , J.‐P. Leauté , J.‐C. Quéro , and V. Ridoux . 2010. “Prey Selection by the Common Dolphin: Fulfilling High Energy Requirements with High Quality Food.” Journal of Experimental Marine Biology and Ecology 390(2): 73–77. 10.1016/j.jembe.2010.05.010.

[eap70216-bib-0117] Sprogis, K. R. , F. Christiansen , H. C. Raudino , H. T. Kobryn , R. S. Wells , and L. Bejder . 2018. “Sex‐Specific Differences in the Seasonal Habitat Use of a Coastal Dolphin Population.” Biodiversity and Conservation 27(14): 3637–3656. 10.1007/s10531-018-1618-7.

[eap70216-bib-0118] Spyrakos, E. , T. C. Santos‐Diniz , G. Martinez‐Iglesias , J. M. Torres‐Palenzuela , and G. J. Pierce . 2011. “Spatiotemporal Patterns of Marine Mammal Distribution in Coastal Waters of Galicia, NW Spain.” Hydrobiologia 670(1): 87–109. 10.1007/s10750-011-0722-4.

[eap70216-bib-0119] Taylor, N. , M. Authier , R. Banga , M. Genu , K. Macleod , and A. Gilles . 2022. “Marine Mammal by‐Catch.” In OSPAR, 2023: The 2023 Quality Status Report for the Northeast Atlantic. London: OSPAR Commission. https://oap.ospar.org/en/ospar‐assessments/quality‐status‐reports/qsr‐2023/indicatorassessments/marine‐mammal‐bycatch.

[eap70216-bib-0120] Torres Ortiz, S. , J. Stedt , H. S. Midtiby , H. D. Egemose , and M. Wahlberg . 2021. “Group Hunting in Harbour Porpoises (*Phocoena phocoena*).” Canadian Journal of Zoology 99(6): 511–520. 10.1139/cjz-2020-0289.

[eap70216-bib-0121] Torres‐Pereira, A. , H. Araújo , S. S. Monteiro , M. Ferreira , J. Bastos‐Santos , S. Sá , L. Nicolau , et al. 2023. “Assessment of Harbour Porpoise Bycatch along the Portuguese and Galician Coast: Insights from Strandings over Two Decades.” Animals 13(16): 2632. 10.3390/ani13162632. 37627422 PMC10451651

[eap70216-bib-0122] Tsagarakis, K. , M. Giannoulaki , S. Somarakis , and A. Machias . 2012. “Variability in Positional, Energetic and Morphometric Descriptors of European Anchovy *Engraulis encrasicolus* Schools Related to Patterns of Diurnal Vertical Migration.” Marine Ecology Progress Series 446: 243–258. 10.3354/meps09456.

[eap70216-bib-0123] Tuck, G. N. , R. B. Thomson , C. Barbraud , K. Delord , M. Louzao , M. Herrera , and H. Weimerskirch . 2015. “An Integrated Assessment Model of Seabird Population Dynamics: Can Individual Heterogeneity in Susceptibility to Fishing Explain Abundance Trends in Crozet Wandering Albatross?” Journal of Applied Ecology 52(4): 950–959. 10.1111/1365-2664.12462.

[eap70216-bib-0124] Vishnyakova, K. , and P. Gol'din . 2015. “Seasonality of Strandings and Bycatch of Harbour Porpoises in the Sea of Azov: The Effects of Fisheries, Weather Conditions, and Life History.” ICES Journal of Marine Science 72(3): 981–991. 10.1093/icesjms/fsu192.

[eap70216-bib-0125] Wade, P. R. , R. R. Reeves , and S. L. Mesnick . 2012. “Social and Behavioural Factors in Cetacean Responses to Overexploitation: Are Odontocetes Less ‘Resilient’ Than Mysticetes?” Journal of Marine Biology 2012: e567276. 10.1155/2012/567276.

[eap70216-bib-0126] Wallace, B. P. , S. S. Heppell , R. L. Lewison , S. Kelez , and L. B. Crowder . 2008. “Impacts of Fisheries Bycatch on Loggerhead Turtles Worldwide Inferred from Reproductive Value Analyses.” Journal of Applied Ecology 45(4): 1076–1085. 10.1111/j.1365-2664.2008.01507.x.

[eap70216-bib-0127] Westgate, A. J. , and A. J. Read . 2007. “Reproduction in Short‐Beaked Common Dolphins (*Delphinus delphis*) from the Western North Atlantic.” Marine Biology 150(5): 1011–1024. 10.1007/s00227-006-0394-1.

[eap70216-bib-0128] Wickham, H. 2016. Ggplot2. Use R! Cham: Springer International Publishing. 10.1007/978-3-319-24277-4.

[eap70216-bib-0129] Zhou, S. , R. M. Daley , M. Fuller , C. M. Bulman , and A. J. Hobday . 2019. “A Data‐Limited Method for Assessing Cumulative Fishing Risk on Bycatch.” ICES Journal of Marine Science 76(4): 837–847. 10.1093/icesjms/fsy206.

[eap70216-bib-0130] Zwolinski, J. , A. Morais , V. Marques , Y. Stratoudakis , and P. G. Fernandes . 2007. “Diel Variation in the Vertical Distribution and Schooling Behaviour of Sardine (*Sardina pilchardus*) Off Portugal.” ICES Journal of Marine Science 64(5): 963–972. 10.1093/icesjms/fsm075.

