# Supplementary material for: Linking by‐caught cetacean traits to fishing techniques: Insights from two species of small cetaceans
Source: Ecol Appl. 2026 Mar 26;36(2):e70216. doi: 10.1002/eap.70216 (PMC13021325; doi:10.1002/eap.70216)
Supplement: Supplementary file 1 — Appendix S1: [file EAP-36-e70216-s001.pdf]

Appendix S1

Linking by-caught cetacean traits to fishing techniques: Insights from two species of small cetaceans

Mathieu Brevet, Matthieu Authier, Hélène Peltier, Laurent Dubroca

Ecological Applications

S1. Datasets pre-processing

Several delphinids taxa other than *Delphinus delphis* and *Phocoena phocoena* were reported as by-catch, including *Stenella coeruleoalba* (N=7), *Tursiops truncatus* (N=4), *Lagenorhynchus acutus* (N=2), *Delphinus capensis* (N=3), *Stenella frontalis* (N=1), *Grampus griseus* (N=1), *Globicephala melas* (N=1) and unidentified delphinids (N=71). However, we did not merge and use all these data as this would have made biological interpretation difficult due to the different ecological characteristics of each species. Before using the datasets for our analyses, we checked the plausibility and consistency of the trait measurements. We paid particular attention to weight, which is estimated on the basis of expert judgement and is therefore more prone to error. For common dolphins, we discarded all weights lower than 3 kg or greater than 300 kg, as well as all values inconsistent with body length measurements (10 kg for 120 cm length and 15 kg for 144 cm length). For harbour porpoises, we discarded all weights greater than 150 kg.

S2. Distribution of by-catch by area and time period

Here, we provide detailed information on the distribution of by-catch by area and time period. When using observation data alone, we corrected these distributions by the observation effort (*i.e.*, soaking time with minute precision; recorded by direct measures of observers for towed gears or by interviewing skippers for passive gears). We only used observations involving fishing gears with an identified risk of capture to estimate observation effort in cases susceptible to capture. This includes fishing gears involved in at least one capture of the studied species within our study area and time period (see Figure S8; which excludes dredges, most line gears and fishing traps). Overall, the duration of at-risk fishing gear soaking time is highly variable in the observer program, with an average of 3 hours and 43 minutes (± 1 hour and 37 minutes SD) for towed gear and an average of 27 hours and 50 minutes (± 48 hours

and 23 minutes SD) for passive gear.
Taking into account the sampling effort in the observation data, the by-catch rates for each species were fairly constant across the ICES divisions (Figure S3), with small variations for common dolphins: by-catch rate was higher along the coasts of the Bay of Biscay (except in division 27.7.k and 27.7.f, which had particularly low observation coverage compared to the other regions). The study periods ranged from 2005 to 2023 for observations, 2019 to 2023 for declarations, and 2000 to 2022 for strandings. We observed an increase in the number of reported common dolphin by-catches over the study period, with a peak in recent years, regardless of the dataset considered (Figure S4). Data were collected throughout the year, but most common dolphin by-catches were reported during the first trimester of the year (Figure S5). Data were also collected throughout the day, but common dolphin by-catches were mostly reported during nighttime or morning fishing (Figure S6). This information is only available for observation data and towed gears because the soaking times of passive gears are too long to determine exact capture timing. There was no strong temporal variation in reported harbour porpoise by-catches, except that by-catches slightly more frequently occurred at the beginning of the year (Figure S5). Data were collected in all sea conditions where fishermen were active, but reports of common dolphin by-catch are particularly low during hauling in calm seas (Figure S7). This information is only available for observational data and towed gears because the soaking times of the passive gears are too long for the entire fishing event to be well represented by the conditions during hauling.

S3. Information retrieval and identification of potential duplicates

We performed a partial data retrieval (information on targeted taxa) for the declarative dataset: missing information from the by-catch declaration files was sometimes available in the fishing activity declaration files (SACROIS). Indeed, declarations on targeted taxa during the fishing trip were often missing from the original dataset, but provided at the fishing operation level in the SACROIS database. When such information was missing, we checked whether the targeted taxon remained consistent throughout the fishing trip of interest in SACROIS. If so, we attributed this value to the corresponding by-catch event in the declaration dataset. We did not estimate missing data from other variables. This is because missing data is likely not randomly distributed (*e.g.*, under-reporting of specific fishing activity profiles, Basran and Sigurðsson, 2021). Therefore, it may be poorly predicted by other variables.
The declaration (contained in the SACROIS database) and observation (OBSMER) datasets do not share the same trip IDs, making it difficult to determine whether there are potential duplicates in these two datasets. To control for duplicates, we used the available information in the OBSMER dataset to infer the potential SACROIS trip IDs associated with the by-catch event. We did this for all OBSMER by-catch events associated with a vessel that was also present in the observed by-catch dataset. We identified the trip IDs in SACROIS that potentially matched the characteristics of the trip of interest (in OBSMER) by using the landing port and date associated with that trip. In most cases, we found a list of potential SACROIS trip IDs corresponding to the trip of interest. We then checked whether any of these trip IDs were included in the declared by-catch trip IDs. If so, we considered the event a potential duplicate and discarded the by-catch events associated with the problematic trips from the declaration datasets (N = 24).
In the observation dataset, we calculated the total catch weight for each fishing operation and for each taxon caught during that operation. The first measure was obtained by summing the measured weight of each species caught during the operation, without considering the weight of fished trash, other marine megafauna species, and non-commercial echinoderms and medusas. The total weight of each taxon caught during a fishing operation was then used to determine the most important taxon in terms of

weight during that fishing operation.

S4. Details on acoustic deterrent devices

Informed presence of an acoustic deterrent device (ADD) was associated with good device functionality, as attested by onboard observers when the vessel was boarded or when the net was hauled aboard. Different types of ADDs were used. For trammel nets, Cetasever PIFIL pingers (https://www.octech.fr) were used in all cases except one, which was discarded from the analyses. These pingers were located on the back of the boat and operated during hauling. Two modules are placed under the rear hull, one on each side, to insonify the rear area of the ship on each side of the wake and, to a lesser extent, the bow (*i.e.*, areas at risk of cetacean by-catch during hauling). For pair trawlers, the devices were mainly STM Dolphin Dissuasive Devices 3 (DDD003H; N = 37; https://www.marintec.co.nz/dolphin-deterrent-devices), and less frequently, PIFIL pingers. The DDD003 devices were attached to the trawl and are sufficiently powerful to be perceptible while trawling, according to the manufacturer's specifications. The device (two modules) is installed on each arm of the trawl to prevent cetaceans from entering inside. The constructors' specifications for the ADDs (*e.g.*, source level) and the users' guidance (which was followed during fishing operations) are available in the previously provided links. ADDs were mainly deployed after 2019 (N = 58), during the first quarter of the year (N = 51), and in the Bay of Biscay (N = 58). These pingers are only active during specific periods of fishing gear movement (hauling or trawling) and therefore only target these specific periods.
The ADD effect on the size of by-caught common dolphins (Figure 5, Table 3) was statistically robust. There was no significant interaction effect with fishing gear when included in models. The effect remained significant when considering each fishing gear class separately or when considering the type of acoustic deterrent device separately (specific to hauling or trawling). Additionally, we observed no confounding effects from spatiotemporal variables, that might have occurred as the effects of the ICES division and trimester on the body length of by-caught individuals (see Figure 2) were similar to the effect of acoustic deterrent devices presence (see Figure 5). Indeed, the device effect persisted when considering only data from the first quarter of the year or from the Bay of Biscay.

S5. Treatment of external variables in declared/observed by-catch datasets

The available by-catch data from direct observations or declarations are quite heterogeneous in time and space. Therefore, we grouped years and ICES divisions before testing for their potential effects on phenotype sensitivity to by-catch. We grouped the ICES divisions (Figure S2) into four sets: the English Channel and southern North Sea (27.7.e, 27.7.d and 27.4.c), the Celtic Sea and southwest Ireland (27.7.f,g,h,j,k), the northern Bay of Biscay (27.8.a,d) and the southern Bay of Biscay (27.8.b,c). The distribution of observed and declared by-catch events across years is quite heterogeneous for common dolphins, with a low number of annual records between 2005 and 2012 and a high number of records in 2021, 2022, and 2023 (due to the availability of new data sources). For these reasons, we considered four groups of years for subsequent analyses on common dolphins: 2005-2012, 2013-2016, 2017-2020, and 2021-2023. For harbour porpoise, the data are more evenly distributed, although there are still a small number of records before 2010, so we considered four groups of years for subsequent analyses on this species: 2005-2010, 2011-2014, 2015-2018, and 2019-2023. Similarly, we grouped sea conditions because there is few data for calm conditions (grouping glassy or rippled sea in the Douglas sea scale) or rough conditions (grouping rough, very rough, or high sea in the Douglas sea scale).

S6. Phenotype vulnerabilities to different fishing techniques: data pre-processing

The first stage of our analysis of the fishing techniques effects on by-caught individuals' phenotypes involved formatting the explanatory variables used in our model. The first variable we considered was the type of fishing gear used when by-catch occurred, which included different types of trawl (bottom or pelagic pair trawl, bottom or pelagic otter trawl, twin bottom otter trawl), seines (Danish seine and purse seine), set net (gillnet and trammel net), and even set longline (Figure S8). To account for the rarest categories and to facilitate the interpretation of potential effects, we have combined these different categories into two main groups: trawls and Danish seines (hereafter referred to as trawls), and set gillnets and trammel nets (hereafter referred to as gillnets). Similarly, by-catch events were distributed heterogeneously among fished taxa. We distinguished two different estimates to determine the main fished taxon during a fishing operation. For observation data, we used the taxon that was primarily caught during the fishing operation, in terms of weight, as measured by the observer. Since this information was unavailable in the declaration dataset, we used the targeted taxon (as declared by fishermen) as a proxy when merging observation and declaration data. It is important to note that this proxy is quite accurate, as there is a 75% overlap between the most fished taxa and the declared targeted taxa at the species level in the observation dataset. As before, we grouped taxa to better account for rare categories. To do this, we used ISSCAAP groups ("International Standard Statistical Classification of Aquatic Animals and Plants", as defined by the FAO in the ASFIS List of Species for Fishery Statistics Purposes, Garibaldi, 2012). Figures S9 and S10 describe the ISSCAAP categories associated with by-catch (respectively for targeted taxa and most fished taxa) and indicate which main taxa are most associated with them. For all models, we excluded rare categories (N<6, after removing all by-catch events with a missing value for the by-caught individual's trait of interest) from the analysis due to insufficient sample size to be considered reliable.

For each model, we tested the collinearity of retained variables using the generalised variance inflation factor (GVIF), and more specifically $GVIF^{1/(2df)}$ when our models included categorical variables with more than two categories (we provided all VIF-related values in Table S1). We considered a metric greater than 2.24 (*i.e.*, a GVIF > 5 for one-degree-of-freedom variables) too high for analysis. In such cases, we removed the variable with the highest $GVIF^{1/(2df)}$ (or GVIF) value.

For harbour porpoise models, the number of by-catch events was more limited than for common dolphins: it results in rare categories for both targeted/fished species and used fishing gear when analysing harbour porpoise by-catch events. Specifically, for the model investigating sex differences, reported events with fishing gear other than gillnets were too rare to be included, so only the targeted taxa variable was retained as an explanatory variable. For BM and BL models, reported by-catch events with trawls often occurred when targeting rare taxa, making it impossible to include both variables (used fishing gear and targeted taxa) in the same model. In these cases, we prioritised the presence of used fishing gear over targeted taxa. It is important to note that models for harbour porpoise (using observation data) did not include the presence of an acoustic deterrent, as there were only two events of harbour porpoise by-catch with an active deterrent device. For the harbour porpoise sex model, we excluded the mesh size from the analyses due to high variance inflation factor values (Table S1).

Before implementing models, we always tested for the presence of potential random effects from spatio-temporal variables, and also from the data sources when using both observation and declaration data in the model. If non-parametric a priori tests were significant (see previous section and Table S9 for details), we then tested for the inclusion of the external variable as a random factor using an analysis of deviance comparison test of the null models, with or without the random factor (Zuur et al., 2009). These tests were not significant (p>0.05) in the two cases where there were spatio-temporal effects on by-caught individual's phenotype (see Table S9). Therefore, we did not include any random effects in

our models.

S7. Alternative, weakly supported explanations of the observed effects

In the case of the harbour porpoise body mass model (and to a lesser extent for the body length model), mesh size is closely linked to targeted taxa: large mesh sizes (greater than 200 mm) are mainly used to target monkfish and only occasionally to target skates and flat fishes. The effect of mesh size we evidenced (larger porpoises being caught in larger mesh size) could then also be linked to potential overlap in the diet of adult harbour porpoises (Santos and Pierce, 2003) and monkfish (Fariña et al., 2008) and/or to specific strategies associated with monkfish fisheries (*e.g.*, several days soaking time and deeper set: Bjørge et al., 2013), which may pose a greater threat to adults as they are known to forage in more offshore areas (Santos and Pierce, 2003).
The differences in the size and weight of by-caught common dolphins observed when fishing for various demersal fish (John Dory, monkfish) are supported by few data points (Figure 5) and may be misleading. Interestingly, we again observed a strong positive correlation between mesh size and targeted taxa in the declaration dataset (high mesh sizes being associated with monkfish fishing, similarly to what we observed in harbour porpoise). This could explain the prediction of capturing individuals with larger BM when fishing for demersal fish (with an underlying mechanical effect of mesh size, as discussed for harbour porpoise).

S8. Better integrate the environmental risk landscape to improve understanding of cetacean by-catch

Our results argue for better consideration of the risk landscape that emerges from the global environmental context (Gilman et al., 2023). Here, we did not consider exogenous variables other than spatio-temporal ones known to be important for marine mammal by-catch (Northridge et al., 2017). This choice was partly out of necessity due to missing data and multicollinearity. The two latter points to the need for better study design and sampling in order to fill the gaps and break collinearity patterns arising in part from lack of control over sampling (*e.g.*, declared data, voluntary schemes for onboard observers). However, variables such as sea state may be important to the dynamics of by-catch (Northridge et al., 2017; see also Figure S7). For instance in observer data, sex distribution ($p = 0.005$, Fisher test) and size ($p = 0.001$, Kruskal-Wallis test) of by-caught common dolphins differed significantly between sea states. More males and larger individuals were by-caught during calm sea conditions than during rough sea conditions (Figure S17). Note that similar effects were only observed in trawls ($p < 0.05$) when trawls and gillnets were considered separately. This may be due to differences in the duration of the fishing operation (7 hours 6 minutes ± 2 hours 49 minutes SD for trawls versus 22 hours 35 minutes ± 17 hours 24 minutes SD for gillnets) and therefore the adequacy of the recorded sea state during hauling to the conditions of the entire fishing operation. These results suggest that individuals may behave differently as a function of sea state, age, and sex, with potentially different risk-taking or detection patterns in the vicinity of fishing gear. Such assumptions need to be confirmed by analyses in that test for an effect of these conditions is independent of the other factors influencing by-catch. In our study, trimester and spatial areas were strongly associated with different sea conditions: calm sea conditions were mostly reported in the Bay of Biscay and rough sea conditions mainly in the Channel Sea; the latter two conditions were also mostly reported in winter (*i.e.*, first trimester). Several other ecological factors have been found to correlate with by-catch risk (see Northridge et al., 2017 for a review):

tidal state (Brennecke et al., 2021) or depth (Bjørge et al., 2013) for harbour porpoise; light conditions (Du Fresne, 2007; see also Figure S6) or depth (Du Fresne, 2007; Brown et al., 2014; Puente et al., 2023) for common dolphins. Other environmental parameters have also been found to be of primary importance in other species, such as turbidity for seals (Luck et al., 2020); or the level of competition in the environment for seabirds (Zhou and Brothers, 2021, 2022); or even the facilitation of by-catch by other species such as diving birds bringing bait to the surface, which then affects other seabird species (Jiménez et al., 2012). These variables may also influence the phenotypic profile of by-caught individuals: for example, in our studied species, sex and age segregation profiles as a function of depth have been observed (Sinéad Murphy et al., 2013; Hall, 2011; Smith and Gaskin, 1983), and may lead to differential phenotypic sensitivity and/or vulnerability to by-catch.

Lastly, we also recommend that the different species at risk of by-catch be investigated separately, as by-catch phenotypic sensitivity and/or vulnerability could strongly differ between them. We showed how the by-catch risk of the two species differed with respect to fishing techniques, phenotypes, and their interactions. Taking into account these interactions can improve conservation measures (Grantham et al., 2008; Gilman et al., 2019). However, we should be cautious to consider all species at risk of by-catch when implementing measures to avoid imposing changes beneficial for one species but detrimental for others (as discussed in Komoroske and Lewison, 2015; Gilman et al., 2019). For example, we evidenced that common dolphins and harbour porpoises were caught in different fishing contexts. Reported common dolphin by-catch was mainly associated with pelagic pair trawls, trammel nets, and set gillnets and with vessels targeting either soles, hake or sea bass; whereas reported harbour porpoise by-catch was mainly associated with trammel nets and with vessels targeting mainly either soles or monkfish. Thus, a shift in fishing efforts towards trammel nets targeting monkfish to mitigate common dolphin by-catch could be highly detrimental to harbour porpoises, which are particularly vulnerable to by-catch in these fisheries. Finally, interactions between species of conservation concern when implementing protection measures require also attention as these interspecific interactions can have major effects on population dynamics (Hollowed et al., 2000; Kanaji et al., 2021). Consequently, it is of significant interest to examine the profiles of phenotypic sensitivity and vulnerability to by-catch in multiple species concurrently, in order to understand the implications this could have on their interactions, particularly if these interactions are phenotype-dependent.

References

Basran, Charla Jean, and Guðjón Már Sigurðsson. 2021. "Using Case Studies to Investigate Cetacean Bycatch/Interaction Under-Reporting in Countries With Reporting Legislation." *Frontiers in Marine Science* 8 (December): 779066. ISSN: 2296-7745. https://doi.org/10.3389/fmars.2021.779066.

Bjørge, Arne, Mette Skern-Mauritzen, and Marjorie C. Rossman. 2013. "Estimated Bycatch of Harbour Porpoise (*Phocoena phocoena*) in Two Coastal Gillnet Fisheries in Norway, 2006–2008. Mitigation and Implications for Conservation." *Biological Conservation* 161 (May): 164–173. ISSN: 0006-3207. https://doi.org/10.1016/j.biocon.2013.03.009.

Brennecke, Dennis, Magnus Wahlberg, Anita Gilles, and Ursula Siebert. 2021. "Age and Lunar Cycle Predict Harbor Porpoise Bycatch in the South-Western Baltic Sea." *PeerJ* 9 (October): e12284. ISSN: 2167-8359. https://doi.org/10.7717/peerj.12284.

Brown, Susie, David Reid, and Emer Rogan. 2014. "Characteristics of Fishing Operations, Environment and Life History Contributing to Small Cetacean Bycatch in the Northeast Atlantic." *PloS One* 9 (8): e104468. ISSN: 1932-6203. https://doi.org/10.1371/journal.pone.0104468.

Du Fresne, Johanna P. 2007. *Factors Affecting Cetacean Bycatch in a New Zealand Trawl Fishery.* Technical report. New Zealand Department of Conservation.

Fariña, A. C., M. Azevedo, J. Landa, R. Duarte, P. Sampedro, G. Costas, M. A. Torres, and L. Cañás. 2008. "Lophius in the World: A Synthesis on the Common Features and Life Strategies." *ICES Journal of Marine Science* 65, no. 7 (October): 1272–1280. ISSN: 1054-3139. https://doi.org/10.1093/icesjms/fsn140.

Fox, John, and Sanford Weisberg. 2019. *An R Companion to Applied Regression.* SAGE Publications. ISBN: 978-1-5443-3648-0.

Garibaldi, Luca. 2012. "The FAO Global Capture Production Database: A Six-Decade Effort to Catch the Trend." *Marine Policy* 36, no. 3 (May): 760–768. ISSN: 0308-597X. https://doi.org/10.1016/j.marpol.2011.10.024.

Gilman, Eric, Milani Chaloupka, Hollie Booth, Martin Hall, Hilario Murua, and Jono Wilson. 2023. "Bycatch-Neutral Fisheries through a Sequential Mitigation Hierarchy." *Marine Policy* 150 (April): 105522. ISSN: 0308-597X. https://doi.org/10.1016/j.marpol.2023.105522.

Gilman, Eric, Milani Chaloupka, Laurent Dagorn, Martin Hall, Alistair Hobday, Michael Musyl, Tony Pitcher, Francois Poisson, Victor Restrepo, and Petri Suuronen. 2019. "Robbing Peter to Pay Paul: Replacing Unintended Cross-Taxa Conflicts with Intentional Tradeoffs by Moving from Piecemeal to Integrated Fisheries Bycatch Management." *Reviews in Fish Biology and Fisheries* 29, no. 1 (March): 93–123. ISSN: 1573-5184. https://doi.org/10.1007/s11160-019-09547-1.

Grantham, Hedley S., Samantha L. Petersen, and Hugh P. Possingham. 2008. "Reducing Bycatch in the South African Pelagic Longline Fishery: The Utility of Different Approaches to Fisheries Closures." *Endangered Species Research* 5, nos. 2-3 (December): 291–299. ISSN: 1863-5407, 1613-4796. https://doi.org/10.3354/esr00159.

Hall, Anna Marie. 2011. "Foraging Behaviour and Reproductive Season Habitat Selection of Northeast Pacific Porpoises." PhD diss., University of British Columbia. https://doi.org/10.14288/1.0072206.

Hollowed, Anne B., Nicholas Bax, Richard Beamish, Jeremy Collie, Michael Fogarty, Patricia Livingston, John Pope, and Jake C. Rice. 2000. "Are Multispecies Models an Improvement on Single-Species Models for Measuring Fishing Impacts on Marine Ecosystems?" *ICES Journal of Marine Science* 57, no. 3 (June): 707–719. ISSN: 1054-3139. https://doi.org/10.1006/jmsc.2000.0734.

Jiménez, Sebastián, Andrés Domingo, Martin Abreu, and Alejandro Brazeiro. 2012. "Bycatch Susceptibility in Pelagic Longline Fisheries: Are Albatrosses Affected by the Diving Behaviour of Medium-Sized Petrels?" *Aquatic Conservation: Marine and Freshwater Ecosystems* 22 (4): 436–445. ISSN: 1099-0755. https://doi.org/10.1002/aqc.2242.

Kanaji, Yu, Hikari Maeda, Hiroshi Okamura, André E. Punt, and Trevor Branch. 2021. "Multiple-Model Stock Assessment Frameworks for Precautionary Management and Conservation on Fishery-Targeted Coastal Dolphin Populations off Japan." *Journal of Applied Ecology* 58 (11): 2479–2492. ISSN: 1365-2664. https://doi.org/10.1111/1365-2664.13982.

Komoroske, Lisa M., and Rebecca L. Lewison. 2015. "Addressing Fisheries Bycatch in a Changing World." *Frontiers in Marine Science* 2 (October): 83. ISSN: 2296-7745. https://doi.org/10.3389/fmars.2015.00083.

Lenth, Russell V. 2024. *emmeans: Estimated Marginal Means, aka Least-Squares Means.* R package version 1.10.3-090003. https://rvlenth.github.io/emmeans/.

Luck, Cian, Michelle Cronin, Martha Gosch, Kieran Healy, Ronan Cosgrove, Oliver Tully, Emer Rogan, and Mark Jessopp. 2020. "Drivers of Spatiotemporal Variability in Bycatch of a Top Marine Predator: First Evidence for the Role of Water Turbidity in Protected Species Bycatch." *Journal of Applied Ecology* 57 (2): 219–228. ISSN: 1365-2664. https://doi.org/10.1111/1365-2664.13544.

Lüdecke, Daniel. 2018. "Ggeffects: Tidy Data Frames of Marginal Effects from Regression Models." *Journal of Open Source Software* 3 (26): 772. ISSN: 2475-9066. https://doi.org/10.21105/joss.00772.

Murphy, S., A. Collet, and E. Rogan. 2005. "Mating Strategy in the Male Common Dolphin (*Delphinus Delphis*): What Gonadal Analysis Tells Us." *Journal of Mammalogy* 86, no. 6 (December 14, 2005): 1247–1258. ISSN: 0022-2372. https://doi.org/10.1644/1545-1542(2005)86[1247:MSITMC]2.0.CO;2.

Murphy, Sinéad, Marie A. C. Petitguyot, Paul D. Jepson, Rob Deaville, Christina Lockyer, James Barnett, Matthew Perkins, Rod Penrose, Nicholas J. Davison, and Cóilín Minto. 2020. "Spatio-Temporal Variability of Harbor Porpoise Life History Parameters in the North-East Atlantic." *Frontiers in Marine Science* 7 (November): 502352. ISSN: 2296-7745. https://doi.org/10.3389/fmars.2020.502352.

Murphy, Sinéad, Eunice Pinn, and Paul D. Jepson. 2013. "The Short-Beaked Common Dolphin (*Delphinus Delphis*) in the North-East Atlantic: Distribution, Ecology, Management and Conservation Status." In *Oceanography and Marine Biology,* 1st ed., edited by R. N. Hughes, D. J. Hughes, and I. P. Smith, 201–288. CRC Press, August. ISBN: 978-0-429-09951-9. https://doi.org/10.1201/b15406-5.

Murphy, Sinéad, and Emer Rogan. 2006. "External Morphology of the Short-Beaked Common Dolphin, *Delphinus Delphis*: Growth, Allometric Relationships and Sexual Dimorphism." *Acta Zoologica* 87 (4): 315–329. ISSN: 1463-6395. https://doi.org/10.1111/j.1463-6395.2006.00245.x.

Murphy, Sinéad, Arliss Winship, Willy Dabin, Paul D. Jepson, Rob Deaville, Robert J. Reid, Chris Spurrier, Emer Rogan, Alfredo López, Angel F. González, Fiona L. Read, Marjan Addink, Monica Silva, Vincent Ridoux, Jennifer A. Learmonth, Graham J. Pierce, and Simon P. Northridge. 2009. "Importance of Biological Parameters in Assessing the Status of *Delphinus Delphis*." *Marine Ecology Progress Series* 388 (August): 273–291. ISSN: 0171-8630, 1616-1599. https://doi.org/10.3354/meps08129.

Northridge, Simon, Alex Coram, Al Kingston, and Rory Crawford. 2017. "Disentangling the causes of protected-species bycatch in gillnet fisheries." *Conservation Biology* 31 (3): 686–695. ISSN: 1523-1739. https://doi.org/10.1111/cobi.12741.

Puente, Esteban, Leire Citores, Elsa Cuende, Iñigo Krug, and Mikel Basterretxea. 2023. "Bycatch of Short-Beaked Common Dolphin (*Delphinus Delphis*) in the Pair Bottom Trawl Fishery of the Bay of Biscay and Its Mitigation with an Active Acoustic Deterrent Device (Pinger)." *Fisheries Research* 267 (November): 106819. ISSN: 0165-7836. https://doi.org/10.1016/j.fishres.2023.106819.

Santos, M. Begoña, and Graham Pierce. 2003. "The Diet of Harbour Porpoise (*Phocoena Phocoena*) in the Northeast Atlantic." *Oceanography and Marine Biology: An Annual Review* 41 (January): 355–390. ISSN: 2154-9125.

Smith, Gary J. D., and David E. Gaskin. 1983. "An Environmental Index for Habitat Utilization by Female Harbour Porpoises with Calves near Deer Island, Bay of Fundy." *Ophelia* (June). ISSN: 0078-5326. https://doi.org/10.1080/00785326.1983.10427221.

Zhou, Can, and Nigel Brothers. 2021. "Interaction Frequency of Seabirds with Longline Fisheries: Risk Factors and Implications for Management." *ICES Journal of Marine Science* 78, no. 4 (August): 1278–1287. ISSN: 1054-3139. https://doi.org/10.1093/icesjms/fsab014.

———. 2022. "Seabird Bycatch Vulnerability in Pelagic Longline Fisheries Based on Modelling of a Long-Term Dataset." *Bird Conservation International* 32, no. 2 (June): 259–274. ISSN: 0959-2709, 1474-0001. https://doi.org/10.1017/S0959270921000046.

Zuur, Alain, Elena N. Ieno, Neil Walker, Anatoly A. Saveliev, and Graham M. Smith. 2009. *Mixed Effects Models and Extensions in Ecology with R.* Springer Science & Business Media, March. ISBN: 978-0-387-87458-6.

Tables and Figures

		Common dolphin			Harbour porpoise		
		Sex (logistic regression)	Body mass (linear regression)	Body length (linear regression)	Sex (logistic regression)	Body mass (linear regression)	Body length (linear regression)
	Df	GVIF (GVIF$^{(1/(2*Df))}$)	GVIF (GVIF$^{(1/(2*Df))}$)	GVIF (GVIF$^{(1/(2*Df))}$)	GVIF	GVIF	GVIF
Used fishing gear	1	2.979 (1.726)	1.901 (1.379)	2.771 (1.665)		1.042	1.172
Mesh size	1	1.483 (1.218)	3.992 (1.998)	1.946 (1.395)	**6.788** / *NA*	1.042	1.93
Targeted taxa (ISSCAAP code)	3		6.585 (1.369)				
Fished taxa (ISSCAAP code)	3-6-1	2.3 (1.149)		3.197 (1.102)	**4.775** / *1.024*		
Soaking time	1	2.037 (1.427)		2.699 (1.643)	2.03 / *1.024*		1.927
Total catch weight	1	1.197 (1.094)		1.338 (1.157)	1.407 / *1*		1.038
Presence of an acoustic deterrent	1	1.251 (1.119)		1.319 (1.149)			

Table S1: Variance inflation factor values from models on by-caught individuals' traits. This table displays generalised variance inflation factor values (*i.e.*, GVIF, as well as GVIF$^{(1/(2*Df))}$ when at least one variable with more than one degree of freedom is present in the model) obtained from models describing the phenotype of by-caught individuals as a function of fishing activity metrics. We used the `car` `R` package (Fox and Weisberg, 2019) to obtain these values. We highlighted in bold values above the acceptable collinearity threshold (see Methods for details). In such cases, we run revised models (removing the variable with the highest GVIF value) to avoid collinearity bias. We reported GVIF values from these revised models after the division sign, in italics.
GVIF: Generalised variance inflation factor; Df: Degrees of freedom; ISSCAAP: International Standard Statistical Classification of Aquatic Animals and Plants (from FAO)

Species	Variable	contrast	estimate	SE	z-ratio	p.value	adjusted p.value
Common dolphin	Trimester	Trimester 1 - Trimester 4	0.340	0.130	2.616	0.009	0.044
		Trimester 2 - Trimester 4	0.465	0.199	2.339	0.019	0.089
		Trimester 3 - Trimester 4	0.360	0.169	2.132	0.033	0.143
	ICES division	27.8.a - 27.8.b	-0.271	0.086	-3.145	0.002	0.005
Harbour porpoise	ICES division	27.7.d - 27.8.b	-0.539	0.202	-2.666	0.008	0.059
		27.7.e - 27.8.b	-0.709	0.335	-2.113	0.035	0.214
		27.8.a - 27.8.b	-0.676	0.251	-2.689	0.007	0.056

Table S2: Post-hoc pairwise comparisons of categories from spatio-temporal variables with a significant effect on stranded individual's sex distribution. This table presents post-hoc pairwise comparison statistics from logistic regression on stranded individuals' sex as a function of spatio-temporal variables, for both studied species. We only performed these tests on statistically significant variables from these models (Table 2). These tests are based on a comparison of the estimated marginal means (the other variables are held at their means) using the `emmeans` `R` package (Lenth, 2024), with Tukey's correction for multiple testing provided ("adjusted p.value" column). We only displayed here significant pairwise comparisons (p.value<0.05).
SE: Standard error

Species	Sex	Variable	contrast	estimate	SE	t-ratio	p.value	adjusted p.value	adj.p.value_transf
Common dolphin	Male	Range of years	(2000-2003) - (2004-2007)	10.022	3.074	3.260	0.001	0.014	0.015
			(2000-2003) - (2012-2015)	7.437	2.700	2.754	0.006	0.066	0.042
			(2000-2003) - (2016-2019)	7.221	2.330	3.099	0.002	0.024	0.022
			(2000-2003) - (2020-2022)	10.338	2.292	4.511	<0.001	<0.001	<0.001
			(2004-2007) - (2008-2011)	-8.699	3.154	-2.758	0.006	0.065	0.153
			(2008-2011) - (2012-2015)	6.114	2.812	2.174	0.030	0.250	0.370
			(2008-2011) - (2016-2019)	5.898	2.439	2.418	0.016	0.150	0.330
			(2008-2011) - (2020-2022)	9.016	2.395	3.765	<0.001	0.002	0.007
			(2016-2019) - (2020-2022)	3.117	1.584	1.968	0.049	0.361	0.256
		Trimester	Trimester1 - Trimester2	15.620	2.718	5.747	<0.001	<0.001	<0.001
			Trimester1 - Trimester3	4.969	2.197	2.262	0.024	0.107	0.153
			Trimester2 - Trimester3	-10.650	3.334	-3.195	0.001	0.008	0.023
			Trimester2 - Trimester4	-13.549	3.481	-3.893	<0.001	0.001	<0.001
		ICES division	27.7.e - 27.8.a	-10.071	2.312	-4.356	<0.001	<0.001	0.001
			27.7.e - 27.8.b	-16.357	2.475	-6.608	<0.001	<0.001	<0.001
			27.8.a - 27.8.b	-6.286	1.433	-4.386	<0.001	<0.001	<0.001
	Female	ICES division	27.7.e - 27.8.a	-8.044	2.242	-3.588	<0.001	0.001	0.003
			27.7.e - 27.8.b	-10.305	2.474	-4.166	<0.001	<0.001	<0.001
Harbour porpoise	Male	Trimester	Trimester1 - Trimester2	7.464	2.213	3.373	0.001	0.004	0.004
			Trimester2 - Trimester3	-6.561	3.269	-2.007	0.045	0.187	0.236
			Trimester2 - Trimester4	-11.986	3.038	-3.945	<0.001	0.001	0.001
		ICES division	27.4.c - 27.7.d	-6.207	2.896	-2.143	0.033	0.204	0.198
			27.4.c - 27.7.e	-14.655	4.788	-3.061	0.002	0.020	0.020
			27.4.c - 27.8.a	-11.619	3.585	-3.241	0.001	0.011	0.009
			27.4.c - 27.8.b	-13.544	3.159	-4.287	<0.001	<0.001	<0.001
			27.7.d - 27.8.b	-7.337	2.397	-3.060	0.002	0.020	0.020
	Female	Range of years	(2004-2007) - (2012-2015)	10.782	5.066	2.128	0.034	0.275	0.194
			(2008-2011) - (2012-2015)	8.208	4.087	2.008	0.045	0.340	0.238
			(2012-2015) - (2020-2022)	-12.067	3.932	-3.069	0.002	0.028	0.019
			(2016-2019) - (2020-2022)	-9.478	3.990	-2.375	0.018	0.168	0.146
		ICES division	27.4.c - 27.7.d	-9.758	4.119	-2.369	0.018	0.127	0.146
			27.4.c - 27.7.e	-15.028	6.341	-2.370	0.018	0.126	0.181
			27.4.c - 27.8.a	-11.660	4.918	-2.371	0.018	0.126	0.153
			27.4.c - 27.8.b	-17.496	4.847	-3.610	<0.001	0.003	0.003
			27.7.d - 27.8.b	-7.739	3.795	-2.039	0.042	0.250	0.228

Table S3: Post-hoc pairwise comparisons of categories from spatio-temporal variables with a significant effect on stranded individual's body length distribution. This table presents post-hoc pairwise comparison statistics from linear models on stranded individuals' body length as a function of spatio-temporal variables, for both studied species and both sexes. We only performed these tests on statistically significant variables from these models (Table 2). These tests are based on a comparison of estimated marginal means (the other variables are held at their means) using the `emmeans` `R` package (Lenth, 2024), with Tukey's correction for multiple testing provided ("adjusted p.value" column). We also provided the adjusted p-values for models with variable transformation ("adj.p.value_transf" column). We only displayed here significant pairwise comparisons (p.value<0.05).
SE: Standard error

Response / Explanatory variables	Response variable transformation	contrast	estimate	SE	t-ratio	p.value	adjusted p.value
BM / Targeted taxa	None	Pleuronectiformes (mainly soles) - Gadiformes (mainly hake)	13.499	6.734	2.005	0.046	0.190
		Pleuronectiformes (mainly soles) - Miscellaneous coastal fishes (mainly sea bass)	47.776	9.906	4.823	<0.001	<0.001
		Gadiformes (mainly hake) - Miscellaneous coastal fishes (mainly sea bass)	34.277	8.568	4.000	<0.001	0.001
		Miscellaneous demersal fishes (mainly monkfishes) - Miscellaneous coastal fishes (mainly sea bass)	58.684	20.216	2.903	0.004	0.021
	Squared root	Pleuronectiformes (mainly soles) - Miscellaneous coastal fishes (mainly sea bass)	2.629	0.514	5.119	<0.001	<0.001
		Gadiformes (mainly hake) - Miscellaneous coastal fishes (mainly sea bass)	1.954	0.444	4.397	<0.001	<0.001
		Miscellaneous demersal fishes (mainly monkfishes) - Miscellaneous coastal fishes (mainly sea bass)	2.630	1.048	2.509	0.013	0.062
BL / Fished taxa	None	Pleuronectiformes (mainly soles) - Miscellaneous coastal fishes (mainly sea bass)	23.030	8.028	2.869	0.005	0.068
		Pleuronectiformes (mainly soles) - Miscellaneous demersal fishes (mainly monkfishes)	29.362	11.516	2.550	0.012	0.149
		Sharks, rays - Miscellaneous coastal fishes (mainly sea bass)	21.065	8.775	2.401	0.017	0.205
		Sharks, rays - Miscellaneous demersal fishes (mainly monkfishes)	27.397	11.731	2.335	0.021	0.234
		Gadiformes (mainly hake) - Miscellaneous coastal fishes (mainly sea bass)	18.325	5.335	3.435	0.001	0.013
		Gadiformes (mainly hake) - Miscellaneous demersal fishes (mainly monkfishes)	24.658	10.126	2.435	0.016	0.191
	Squared	Pleuronectiformes (mainly soles) - Miscellaneous coastal fishes (mainly sea bass)	8192.072	2736.807	2.993	0.003	0.049
		Pleuronectiformes (mainly soles) - Miscellaneous demersal fishes (mainly monkfishes)	10610.911	3925.947	2.703	0.008	0.104
		Sharks, rays - Miscellaneous coastal fishes (mainly sea bass)	7793.150	2991.498	2.605	0.010	0.131
		Sharks, rays - Miscellaneous demersal fishes (mainly monkfishes)	10211.989	3999.292	2.553	0.012	0.147
		Gadiformes (mainly hake) - Miscellaneous coastal fishes (mainly sea bass)	6778.334	1818.809	3.727	<0.001	0.005
		Gadiformes (mainly hake) - Miscellaneous demersal fishes (mainly monkfishes)	9197.173	3452.072	2.664	0.008	0.114

Table S4: Post-hoc pairwise comparisons of targeted/fished ISSCAAP groups with a significant effect on by-caught common dolphin's BM/BL distribution. This table presents post-hoc pairwise comparison statistics from linear models on by-caught common dolphin's BM/BL as a function of targeted/fished ISSCAAP groups, both with and without transformation of the response variable. These tests are based on a comparison of estimated marginal means (with the other variables held at their means) using the `emmeans` `R` package (Lenth, 2024), with Tukey's correction for multiple testing provided ("adjusted p.value" column). We also provided the adjusted p-values for models with variable transformation ("adj.p.value_transf" column). We only displayed here significant pairwise comparisons (p.value<0.05).
SE: Standard error

Species	Sex	Data	Sample size	Mean BL (standard deviation)	BL range	Median BL	Age estimated from median BL	Proportion of BL above BL at sexual maturity
Common dolphin	Female	Strandings	1302	180.7 (22.8)	94 - 280	185	3.72 - 5.34	0.375 - 0.439
		Observations and declarations	79	179.7 (24.2)	100 - 224	186	3.86 - 5.55	0.354 - 0.456
	Male	Strandings	1758	187.1 (26.8)	85 - 251	191	3.21 - 5.01	0.313
		Observations and declarations	79	183.8 (31.4)	84 - 239	189	3.03 - 4.68	0.278
Harbour porpoise	Female	Strandings	338	133.5 (24.2)	53 - 189	130	2.51 - 3.2	0.317 - 0.385
		Observations and declarations	13	144.8 (30)	98 - 194	150	5.46 - 7.98	0.538 - 0.538
	Male	Strandings	452	130.5 (20.3)	67 - 193	128	2.53 - 3.19	0.367 - 0.462
		Observations and declarations	18	146.9 (20.4)	116 - 190	150	NA - NA	0.722 - 0.722

Table S5: Descriptive statistics on body length of by-caught individuals, by species, sex and data source. This table presents descriptive statistics on body length (number of observations, mean, standard deviation, range, median, estimated age from median, and proportion above the body length at sexual maturity) by species, sex, and data source. Figure 1 provides a visual representation of most of these statistics. We estimated ages at median body length using Gompertz curve equations from the literature (Sinéad Murphy et al., 2009 - Murphy and Rogan, 2006 for female common dolphins; S. Murphy et al., 2005 - Murphy and Rogan, 2006 for male common dolphins; Sinéad Murphy et al., 2020 for harbour porpoises). The values indicated refer to maximum and minimum estimated ages, obtained either from distinct references for common dolphins or from different areas (the Irish and Celtic Seas - the North Sea) for harbour porpoises. We could not estimate age when the median body length exceeds the body length attained at physical maturity (*i.e.*, the asymptotic body length), as was the case for male harbour porpoises in observation and declaration data. The proportion of individuals with a body length below the body length at sexual maturity was computed using the minimum and maximum estimates of body length at sexual maturity found in the literature (same values for male common dolphins), as summarised in Table 1.
BL: Body length

Variable	Sex	Class	Sample size	Mean BL (standard deviation) - observed	BL marginal mean (standard error) - predicted	BL range	Median BL	Age from median BL (or marginal mean)	Proportion of BL above BL at sexual maturity
ICES division	Female	27.7.e	124	172 (27.1)	172.2 (2.3)	109 - 225	177.5	2.92 - 4.14 (2.53 - 3.53)	0.29 - 0.323
		27.8.a	816	180.9 (22.1)	180.3 (1.2)	94 - 280	185.0	3.72 - 5.34 (3.17 - 4.53)	0.357 - 0.426
		27.8.b	361	183.4 (22)	182.5 (1.6)	115 - 236	189.0	4.42 - 6.32 (3.4 - 4.88)	0.446 - 0.507
	Male	27.7.e	146	174.6 (32)	172.8 (2.3)	85 - 240	177.5	2.21 - 3.31 (1.95 - 2.9)	0.219
		27.8.a	1001	184.9 (25.6)	182.8 (1.2)	104 - 251	188.0	2.94 - 4.53 (2.55 - 3.86)	0.257
		27.8.b	607	193.5 (25.7)	189.1 (1.5)	90 - 243	198.0	4.01 - 6.62 (3.04 - 4.7)	0.428
Trimester	Male	1	1364	189.4 (25.2)	187.2 (1)	99 - 243	193.0	3.41 - 5.38 (2.88 - 4.42)	0.34
		2	98	172.8 (30.5)	171.6 (2.7)	110 - 251	174.5	2.04 - 3.04 (1.89 - 2.8)	0.173
		3	164	180 (27.5)	182.3 (2.2)	90 - 250	181.0	2.43 - 3.66 (2.52 - 3.81)	0.183
		4	132	182.6 (32.8)	185.2 (2.3)	85 - 246	187.0	2.86 - 4.39 (2.72 - 4.15)	0.303
Range of years	Male	2000-2003	167	196.4 (23.1)	187.6 (2.3)	123 - 235	199.0	4.16 - 6.96 (2.91 - 4.47)	0.437
		2004-2007	123	183.6 (29)	177.6 (2.5)	85 - 251	188.0	2.94 - 4.53 (2.22 - 3.32)	0.244
		2008-2011	147	194.1 (20.3)	186.3 (2.4)	130 - 240	197.0	3.88 - 6.32 (2.81 - 4.29)	0.395
		2012-2015	210	189.2 (26.9)	180.2 (2)	114 - 243	194.0	3.52 - 5.58 (2.38 - 3.58)	0.39
		2016-2019	477	187.5 (27.3)	180.4 (1.6)	90 - 240	191.0	3.21 - 5.01 (2.39 - 3.6)	0.338
		2020-2022	634	182.6 (27.1)	177.3 (1.4)	98 - 250	186.0	2.78 - 4.25 (2.2 - 3.29)	0.232

Table S6: Descriptive statistics on the body length of by-caught common dolphins, by significant categorical variable class from the spatio-temporal by-catch sensitivity model. This table presents descriptive statistics on body length (number of observations; observed mean and standard deviation; predicted marginal mean and standard error from the spatio-temporal by-catch sensitivity model; observed range and median; estimated age from observed median and predicted marginal mean; and observed proportion above the body length at sexual maturity) by sex and significant categorical variable class from the spatio-temporal by-catch sensitivity model (see Table 2 and Figure 2). Figure 2 provides a visual representation of predicted statistics presented here. We estimated ages at observed median body length or predicted marginal means using Gompertz curve equations from the literature (Sinéad Murphy et al., 2009 - Murphy and Rogan, 2006 for female common dolphins; S. Murphy et al., 2005 - Murphy and Rogan, 2006 for male common dolphins). The values indicated refer to maximum and minimum estimated ages, obtained from distinct references. The proportion of individuals with a body length below the body length at sexual maturity was computed using the minimum and maximum estimates of body length at sexual maturity found in the literature (same values for male common dolphins), as summarised in Table 1.
BL: Body length

Variable	Sex	Class	Sample size	Mean BL (standard deviation) - observed	BL marginal mean (standard error) - predicted	BL range	Median BL	Age from median BL (or marginal mean)	Proportion of BL above BL at sexual maturity
ICES division	Female	27.4.c	43	121.3 (19)	124.9 (4.1)	96 - 172	118.00	1.46 - 1.93 (2.03 - 2.61)	0.116 - 0.163
		27.7.d	160	132.5 (24.7)	134.6 (2.4)	75 - 186	128.00	2.31 - 2.96 (3 - 3.84)	0.325 - 0.344
		27.7.e	21	138.4 (29.8)	139.9 (5.3)	53 - 188	136.00	3.16 - 4.06 (3.66 - 4.76)	0.429 - 0.476
		27.8.a	58	136.2 (24.6)	136.5 (3.2)	84 - 187	140.00	3.68 - 4.78 (3.22 - 4.15)	0.362 - 0.517
		27.8.b	55	141.8 (19.6)	142.4 (3.5)	109 - 189	140.00	3.68 - 4.78 (4.03 - 5.31)	0.364 - 0.509
	Male	27.4.c	60	121.7 (18.8)	123.5 (2.7)	84 - 193	120.00	1.76 - 2.18 (2.07 - 2.57)	0.217 - 0.267
		27.7.d	198	128.1 (20.6)	129.7 (1.7)	67 - 174	127.50	2.47 - 3.11 (2.73 - 3.48)	0.343 - 0.455
		27.7.e	23	137 (22.3)	138.2 (4.1)	100 - 191	130.00	2.77 - 3.53 (4.17 - 6.31)	0.478 - 0.522
		27.8.a	61	134.2 (17.1)	135.1 (2.6)	95 - 168	129.00	2.64 - 3.36 (3.53 - 4.8)	0.443 - 0.492
		27.8.b	110	136.2 (19.7)	137 (2.2)	98 - 180.5	131.00	2.9 - 3.73 (NA - NA)	0.427 - 0.555
Trimester	Male	1	208	132.8 (18.5)	133.7 (1.7)	67 - 180.5	130.00	2.77 - 3.53 (3.29 - 4.37)	0.413 - 0.51
		2	131	123.9 (18.5)	126.2 (2)	84 - 174	121.00	1.84 - 2.29 (2.33 - 2.92)	0.206 - 0.305
		3	51	130 (24.9)	132.8 (3)	83 - 193	128.00	2.53 - 3.19 (3.15 - 4.13)	0.392 - 0.471
		4	62	136.7 (22.5)	138.2 (2.7)	87 - 174	138.50	4.25 - 6.54 (4.17 - 6.31)	0.532 - 0.629
Range of years	Male	2000-2003	18	134 (18.7)	136.1 (4.8)	106 - 174	128.50	2.59 - 3.27 (3.72 - 5.18)	0.444 - 0.444
		2004-2007	50	131 (17.3)	131.2 (2.9)	87 - 174	128.50	2.59 - 3.27 (2.92 - 3.77)	0.32 - 0.48
		2008-2011	55	128.9 (19.6)	130.7 (2.7)	84 - 191	126.00	2.31 - 2.89 (2.86 - 3.67)	0.273 - 0.364
		2012-2015	176	128.2 (21.1)	130.8 (1.8)	85 - 193	126.75	2.39 - 3 (2.87 - 3.69)	0.347 - 0.443
		2016-2019	103	133.2 (20.1)	133.6 (2.1)	84 - 168	130.00	2.77 - 3.53 (3.28 - 4.34)	0.447 - 0.505
		2020-2022	50	132.7 (21.8)	133.9 (3)	67 - 168	131.50	2.96 - 3.83 (3.32 - 4.43)	0.4 - 0.54

Table S7: Descriptive statistics on the body length of by-caught harbour porpoises, by significant categorical variable class from the spatio-temporal by-catch sensitivity model. This table presents descriptive statistics on body length (number of observations; observed mean and standard deviation; predicted marginal mean and standard error from the spatio-temporal by-catch sensitivity model; observed range and median; estimated age from observed median and predicted marginal mean; and observed proportion above the body length at sexual maturity) by sex and significant categorical variable class from the spatio-temporal by-catch sensitivity model (see Table 2 and Figure 3). Figure 3 provides a visual representation of predicted statistics presented here. We estimated ages at observed median body length or predicted marginal means using Gompertz curve equations from the literature (Sinéad Murphy et al., 2020). The values indicated refer to maximum and minimum estimated ages, obtained from distinct areas (the Irish and Celtic Seas - the North Sea). We could not estimate age when the median body length exceeds the body length attained at physical maturity (*i.e.*, the asymptotic body length), as was the case for male harbour porpoises in the ICES division 27.8.b. The proportion of individuals with a body length below the body length at sexual maturity was computed using the minimum and maximum estimates of body length at sexual maturity found in the literature, as summarised in Table 1.
BL: Body length

Species	Variable	Class	Sample size	Mean BL (SD) - observed	BL marginal mean (SE) - predicted	BL range	Median BL	Age (female / male) from median BL (or marginal mean)	Proportion of BL (female / male) above BL at SM
Harbour porpoise	Gear type	Gillnets	50	142.7 (23.1)	141.9 (3.2)	98 - 194	142.5	4.04 - 5.33 / 5.63 - NA (3.95 - 5.19 / 5.36 - NA)	0.48 - 0.56 / 0.6 - 0.7
		Trawls	6	161.2 (12.5)	166.5 (9.6)	150 - 180	158.5	8.69 - NA / NA - NA (NA - NA / NA - NA)	1 - 1 / 1 - 1
Common dolphin	Gear type	Gillnets	76	169.8 (24.8)	165.8 (5.4)	110 - 225	172.0	2.51 - 3.51 / 1.91 - 2.83 (2.15 - 2.93 / 1.6 - 2.37)	0.171 - 0.211 / 0.0789
		Trawls	142	183.2 (28.2)	187 (3.6)	84 - 239	190.0	4.66 - 6.63 / 3.12 - 4.84 (4.03 - 5.78 / 2.86 - 4.39)	0.458 - 0.535 / 0.197
	ADD presence	FALSE	139	174.2 (28.1)	170.7 (3)	84 - 239	175.0	2.72 - 3.83 / 2.07 - 3.08 (2.43 - 3.38 / 1.84 - 2.73)	0.281 - 0.367 / 0.122
		TRUE	56	190.5 (23.5)	182.1 (4.8)	112 - 235	189.5	4.53 - 6.47 / 3.07 - 4.76 (3.36 - 4.81 / 2.5 - 3.78)	0.5 - 0.5 / 0.286
	Most caught ISSCAAP group	31 - Pleuronectiformes	26	173.6 (21.4)	189.3 (6.1)	123 - 222	171.0	2.45 - 3.41 / 1.85 - 2.75 (4.49 - 6.41 / 3.06 - 4.73)	0.192 - 0.269 / 0.0769
		32 - Gadiformes	76	186.9 (27.4)	184.6 (3.1)	100 - 239	181.0	3.24 - 4.63 / 2.43 - 3.66 (3.66 - 5.26 / 2.68 - 4.08)	0.461 - 0.474 / 0.289
		33 - Miscellaneous coastal fishes	61	174.6 (26.9)	166.2 (4.7)	84 - 210	185.0	3.72 - 5.34 / 2.71 - 4.13 (2.17 - 2.96 / 1.62 - 2.4)	0.344 - 0.475 / 0.0328
		34 - Miscellaneous demersal fishes	10	163.5 (27.5)	159.9 (9.9)	120 - 200	170.0	2.39 - 3.31 / 1.8 - 2.68 (1.87 - 2.47 / 1.35 - 1.98)	0.2 - 0.2 / 0
		36 - Tunas, bonitos	10	163.5 (27.2)	177.9 (11)	123 - 206	157.0	1.74 - 2.27 / 1.23 - 1.81 (2.95 - 4.19 / 2.24 - 3.35)	0.1 - 0.2 / 0.1
		37 - Miscellaneous pelagic fishes	8	175 (28.4)	169.5 (9.4)	110 - 208	181.0	3.24 - 4.63 / 2.43 - 3.66 (2.36 - 3.26 / 1.78 - 2.64)	0.125 - 0.125 / 0.125
		38 - Sharks, rays	12	187.4 (32)	187.3 (8.2)	120 - 232	196.0	NA - 10.7 / 3.75 - 6.05 (4.08 - 5.86 / 2.89 - 4.43)	0.583 - 0.583 / 0.333

Table S8: Descriptive statistics on the body length of by-caught individuals, by species and significant categorical variable class from the by-catch vulnerability models. This table presents descriptive statistics on body length (number of observations; observed mean and standard deviation; predicted marginal mean and standard error from the by-catch vulnerability models; observed range and median; estimated age from observed median and predicted marginal mean; and observed proportion above the body length at sexual maturity) by species and significant categorical variable class from the by-catch vulnerability models (see Table 3 and Figures 4-5). The provided statistics are for individuals of all sexes. We did not segregate the statistics by sex because the data contained too many missing values. Figures 4 and 5 provides a visual representation of predicted statistics presented here. Of note, the observed and predicted means differ greatly for most fished taxon categories. This difference likely stems from the use of predicted marginal means, which control for the values of other variables, thereby preventing Simpson's paradox from occurring due to confounding factors. We estimated ages at observed median body length or predicted marginal means using Gompertz curve equations from the literature (Sinéad Murphy et al., 2009 - Murphy and Rogan, 2006 for female common dolphins; S. Murphy et al., 2005 - Murphy and Rogan, 2006 for male common dolphins; Sinéad Murphy et al., 2020 for harbour porpoises). The values separated by a dash refer to maximum and minimum estimated ages, obtained either from distinct references for common dolphins or from different areas (the Irish and Celtic Seas - the North Sea) for harbour porpoises. We could not estimate age when the median body length exceeds the body length attained at physical maturity (*i.e.*, the asymptotic body length), as indicated by NA symbols. The proportion of individuals with a body length below the body length at sexual maturity was computed using the minimum and maximum estimates of body length at sexual maturity found in the literature (same values for male common dolphins), as summarised in Table 1. As data on the sex of by-caught individuals are often missing in the observation dataset, we provided age estimates and the proportion of sexually mature individuals by considering two hypothetical sets of individuals: one consisting of only females and the other consisting of only males (respectively before and after the slash symbol). The actual estimate should range between these theoretical values.

BL: Body length; SM: Sexual maturity; SD: Standard deviation; SE: Standard error; ADD: Acoustic deterrent device; ISSCAAP: International Standard Statistical Classification of Aquatic Animals and Plants (from FAO)

		Common dolphin			Harbour porpoise		
		Sex (Fisher test)	BM (Kruskal-Wallis test)	BL (Kruskal-Wallis test)	Sex (Fisher test)	BM (Kruskal-Wallis test)	BL (Kruskal-Wallis test)
Variable	Df	P-value	Chi-squared (p-value)	Chi-squared (p-value)	P-value	Chi-squared (p-value)	Chi-squared (p-value)
ICES division	3	0.225	4.358 (0.225)	4.757 (0.19)	0.613	0.413 (0.938)	1.918 (0.59)
Trimester	3	0.275	0.334 (0.954)	**10.419 (0.015)**	0.621	5.426 (0.143)	0.686 (0.876)
Time period (range of years)	3	0.091	**16.591 (0.001)**	4.89 (0.18)	0.196	0.836 (0.841)	2.86 (0.414)
Data source	1		0.903 (0.342)			0.078 (0.781)	
Number of observations		163	264	221	32	94	56

Table S9: Non-parametric tests of spatio-temporal (and source) effects on the phenotype of by-caught individuals. This table presents non-parametric test statistics for potential spatio-temporal effects on the phenotype of declared/observed by-caught individuals, for both species studied. We also included the test for potential data source effects when both declaration and observation data were included in the analysis (BM only). We used Fisher tests to test for effects on sex and Kruskal-Wallis tests for size and weight. We highlighted in bold significant effects (p-value<0.05).
BM: Body mass; BM: Body mass; Df: Degrees of freedom

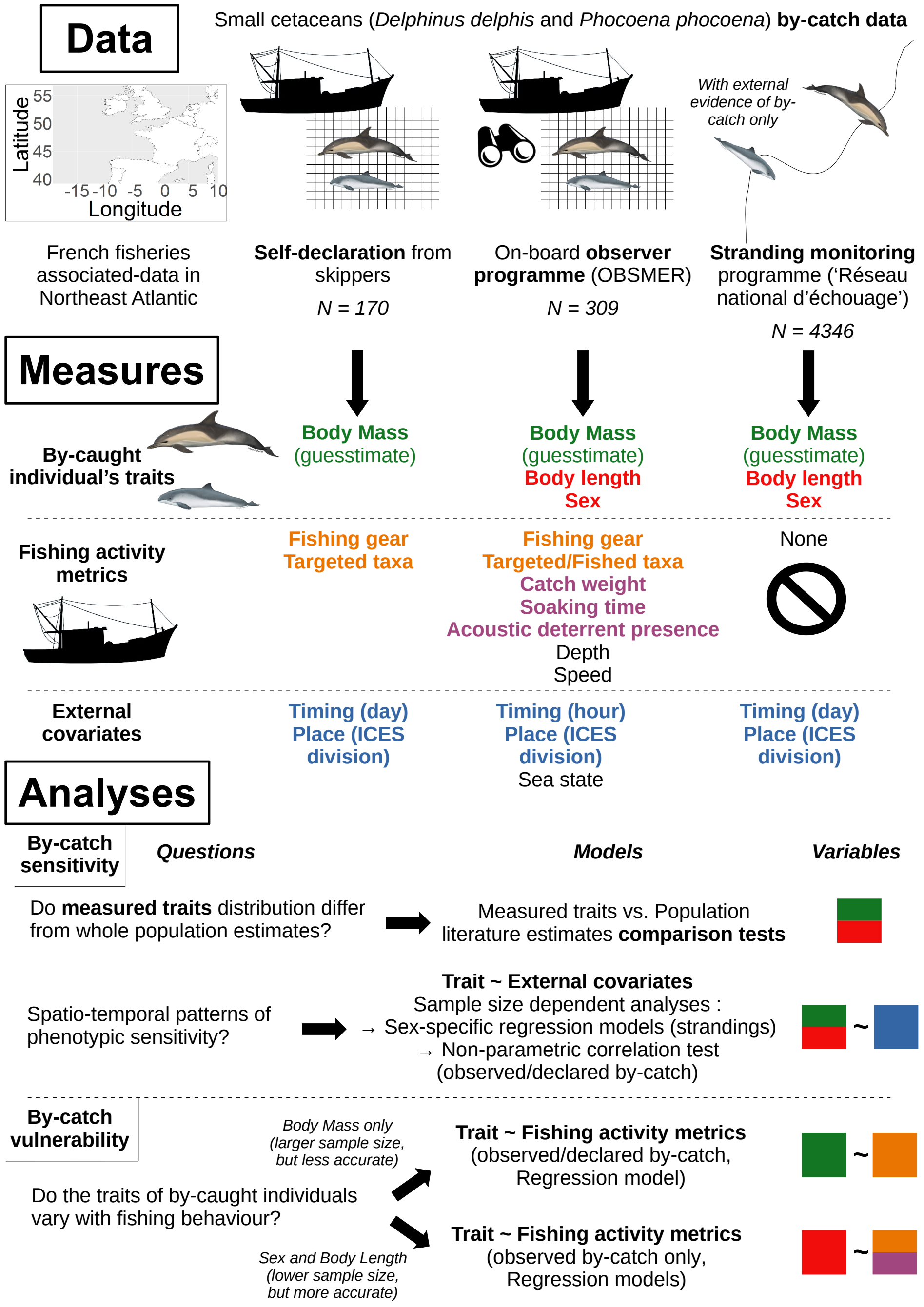

Figure S1: Graphical summary of the used analytical framework. This figure shows the analytical framework used in the study. The first part presents the data used, the second the types of measurements associated with them, and the third the main statistical analyses carried out. In this last part, the different measurements used for each analysis are represented by a colour code corresponding to that used in the previous section. Images of studied small cetaceans (https://www.fisheries.noaa.gov/species/short-beaked-common-dolphin and https://www.fisheries.noaa.gov/species/harbor-porpoise) are in the public domain (U.S. National Oceanic and Atmospheric Administration Fisheries; https://www.noaa.gov/digital-collections/photo-library). The binoculars illustration is an image obtained from https://openclipart.org, produced by Last-Dino, and is in the public domain. The fishing boat silhouette was obtained from Creazilla (https://creazilla.com/media/silhouette/2467/fishing-boat) under a Creative Commons BY 4.0 license (https://creativecommons.org/licenses/by/4.0/deed.en) and was produced by Natasha Sinegina. The map vignette was produced using R software.

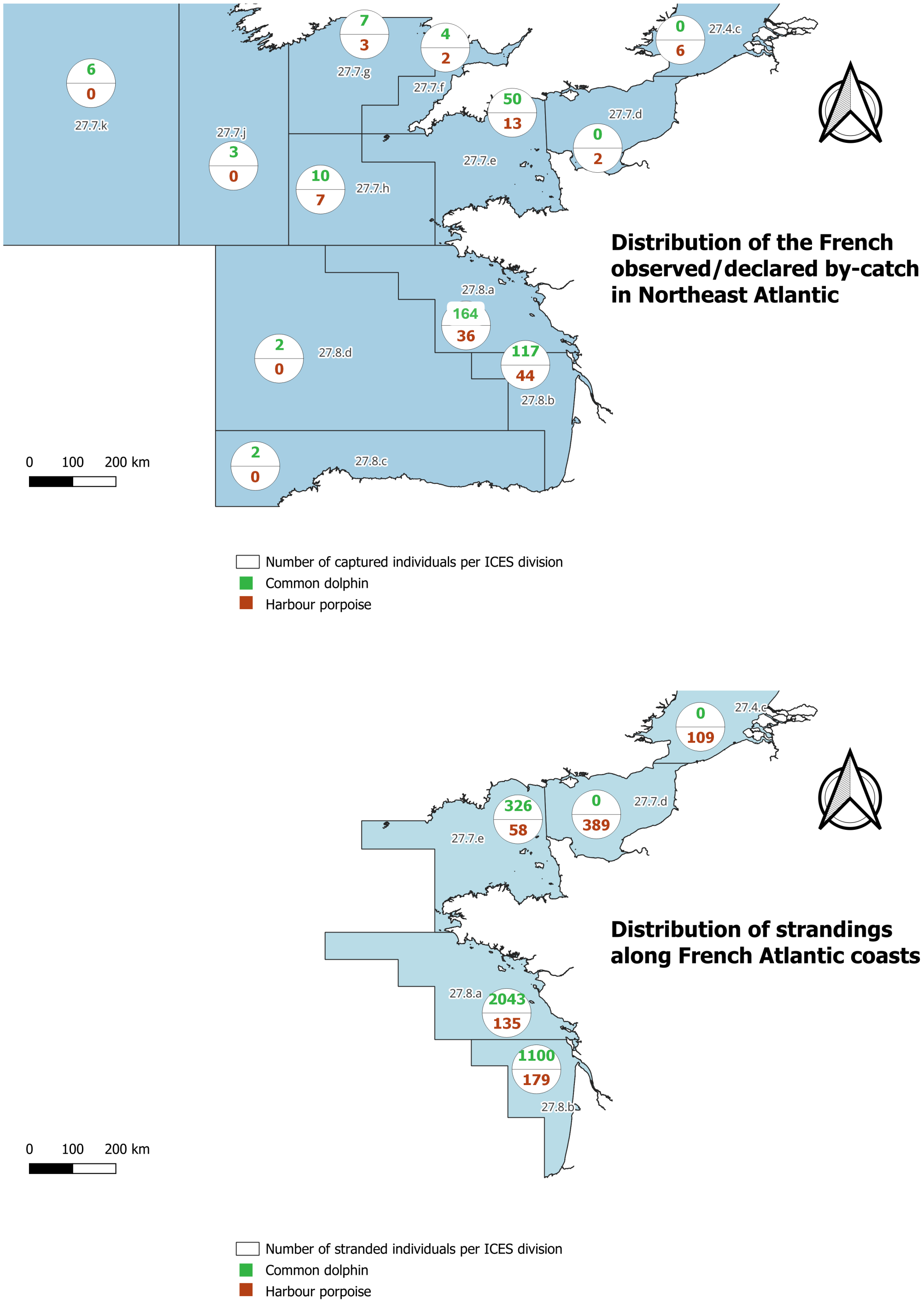

Figure S2: Spatial distribution of reported by-caught individuals. These maps show the distribution of all individually reported by-catch events (top graph) from the observation and declaration datasets, and of all stranded individuals with external evidence of by-catch (bottom graph). The number of individuals is provided by ICES divisions (with black labels on the map) and by species studied (number in green for common dolphin, red for harbour porpoise). In both cases, we only considered spatially located data with available phenotypic measurements. For stranded individuals, the indicated ICES division corresponds to where the individual was found stranded. This is not necessarily where it was by-caught, but it gives a good general indication of the capture area. We produced these maps using QGIS software.

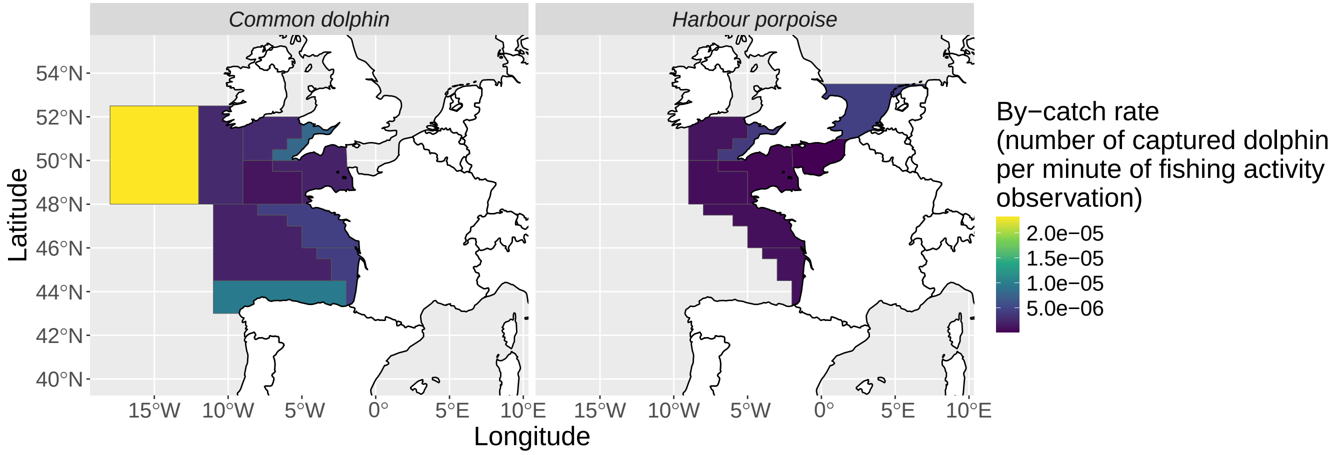

Figure S3: Spatial variation of observed by-catch rates. These maps show the variation in observed by-catch rates (with phenotypic measurements) between ICES divisions (refer to Figure S2 for division IDs) for each studied species. We used only by-catches from the observation dataset. The number of by-catches per ICES division was then divided by the total monitored soaking time summed for each ICES division to obtain estimates of by-catch rates.

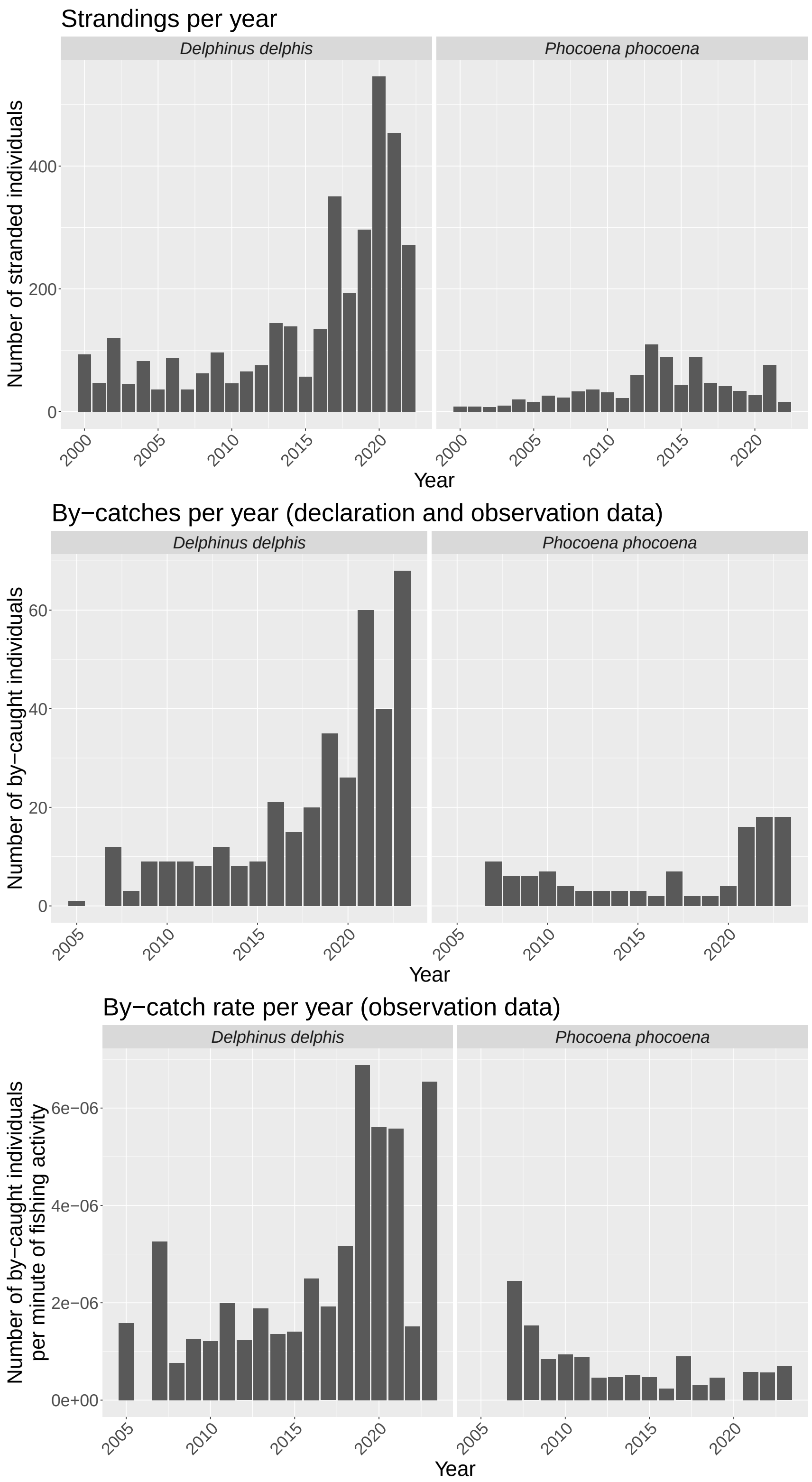

Figure S4: Annual distribution of reported by-catch. Barplots displaying the distribution of reported by-catch (with phenotypic measurements) over the years, respectively for common dolphin (left graphs) and harbour porpoise (right graphs), considering the different data sources (strandings with evidence of by-catch: top graphs; observed and declared by-catch: middle graphs; observed by-catch rate: bottom graphs). By-catch rates were obtained by dividing the number of observed by-catches by the total monitored soaking time summed for each year.

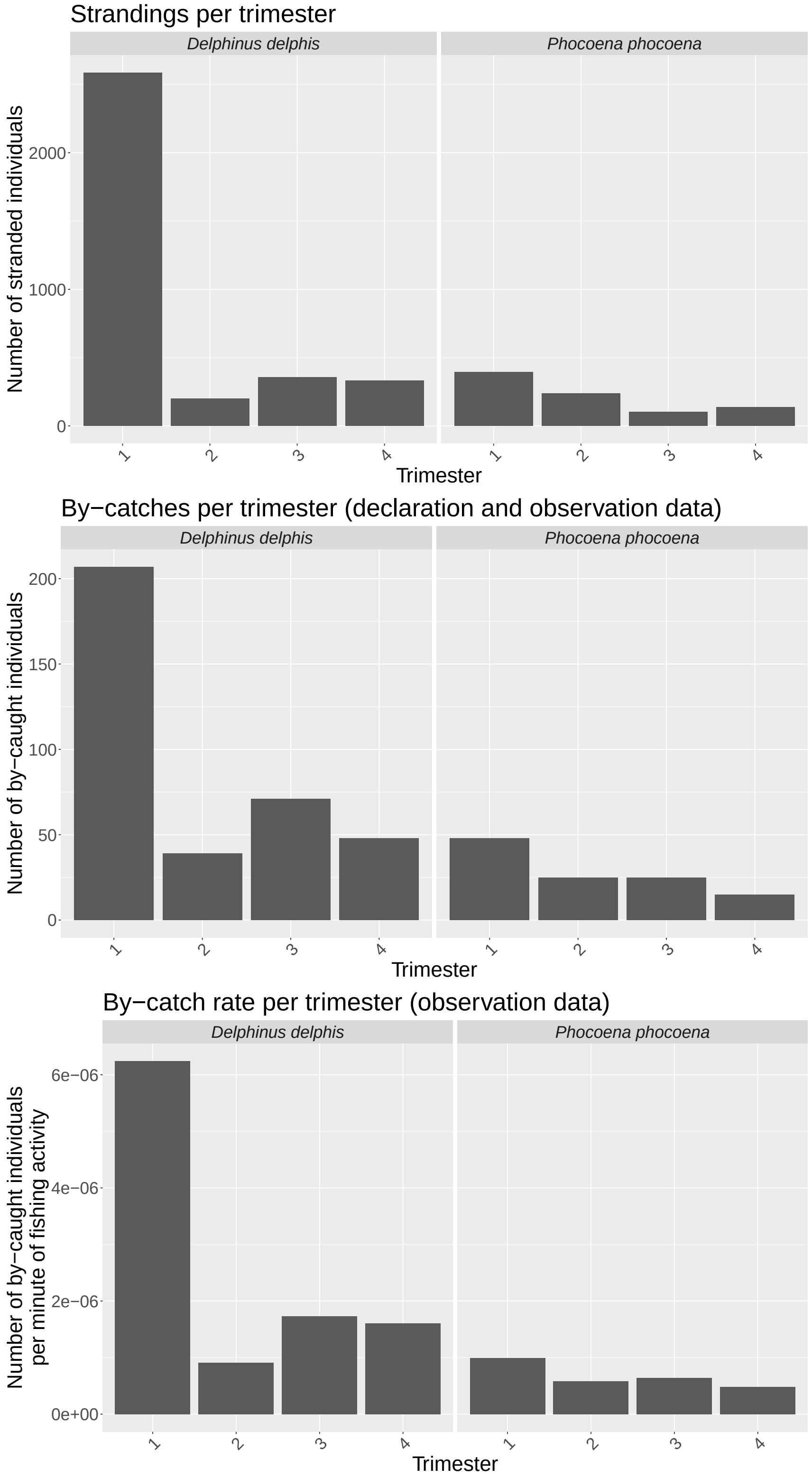

Figure S5: Trimestrial distribution of reported by-catch. Barplots displaying the distribution of reported by-catch (with phenotypic measurements) over trimesters, respectively for common dolphin (left graphs) and harbour porpoise (right graphs), considering the different data sources (strandings with evidence of by-catch: top graphs; observed and declared by-catch: middle graphs; observed by-catch rate: bottom graphs). By-catch rates were obtained by dividing the number of observed by-catches by the total monitored soaking time summed for each trimester.

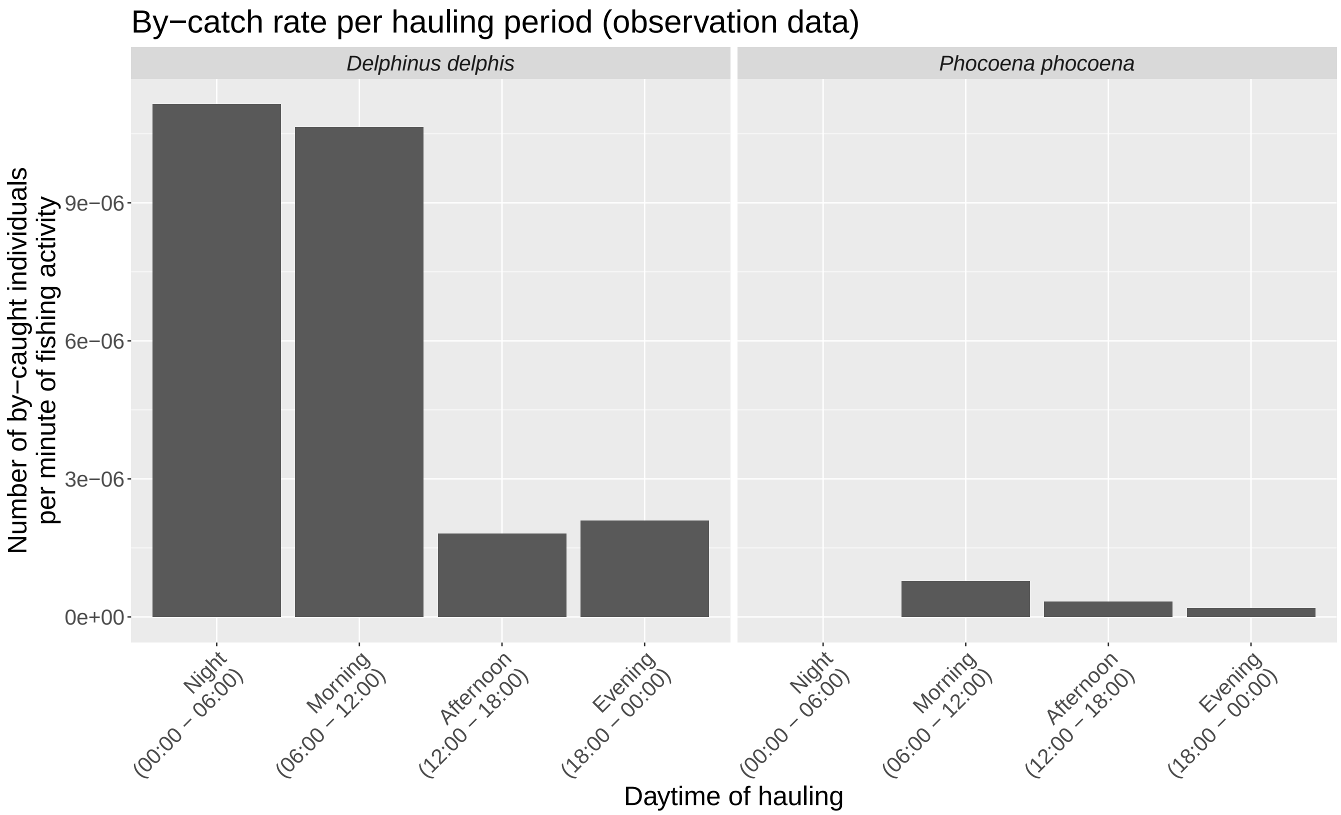

Figure S6: Daily distribution of observed by-catch when using towed gears. Barplots displaying the distribution of observed by-catch (with phenotypic measurements) throughout the day, when using towed gears. The plots are shown separately for the common dolphin (left graph) and the harbour porpoise (right graph). The time of day is represented by the median time of the fishing operation, which is representative of the main activity period for most observed towed gear operations, as more than 99.9% of them last less than 12 hours. The day is divided into four six-hour periods: night (00:00 to 06:00), morning (06:00 to 12:00), afternoon (12:00 to 18:00), and evening (18:00 to 00:00). These data are only available in the observation dataset, so we only presented by-catch rates here. It was only done for towed gears as the soaking times of passive gears are too long to determine exact capture timing (see Appendix, section S2). By-catch rates were obtained by dividing the number of observed by-catches by the total monitored soaking time (of towed gears) summed for each period of the day.

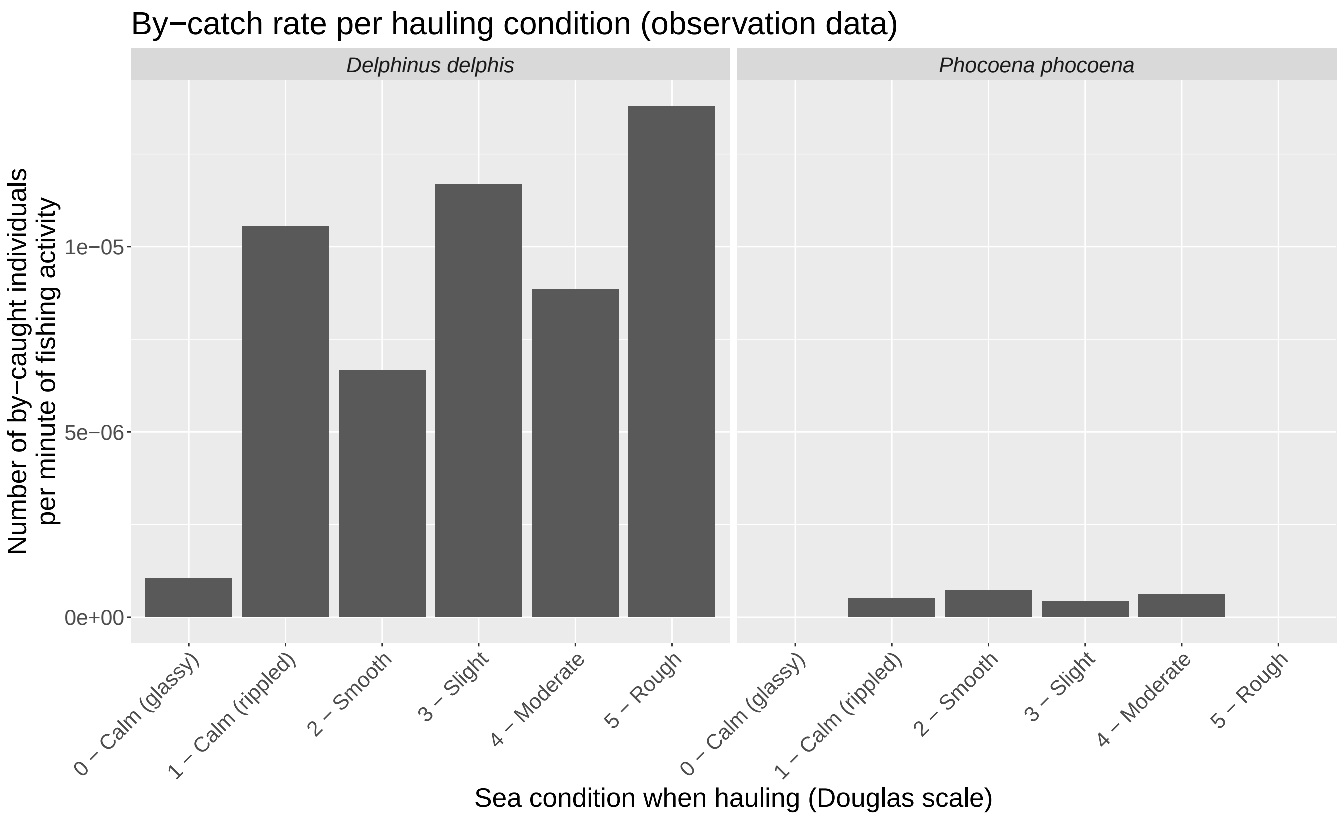

Figure S7: Distribution of observed by-catch by sea state when using towed gears. Barplots displaying the distribution of reported by-catch (with phenotypic measurements) across sea conditions, when using towed gears. The plots are shown separately for the common dolphin (left graph) and the harbour porpoise (right graph). The sea state was recorded when hauling occurred. The Douglas scale was used to describe the sea state. These data are only available in the observation dataset; therefore, we only present here by-catch rates. Overall, 13% of the values in the entire observation dataset were missing (though less than 1% were missing for events with by-catch), primarily in the most remote years. Sea state was never reported before 2006 but was almost always reported after 2018. We only displayed by-catch rates in towed gears as the soaking times of passive gears are too long for the entire fishing event to be well represented by the conditions during hauling. By-catch rates were obtained by dividing the number of observed by-catches by the total monitored soaking time (of towed gears) summed for each sea state.

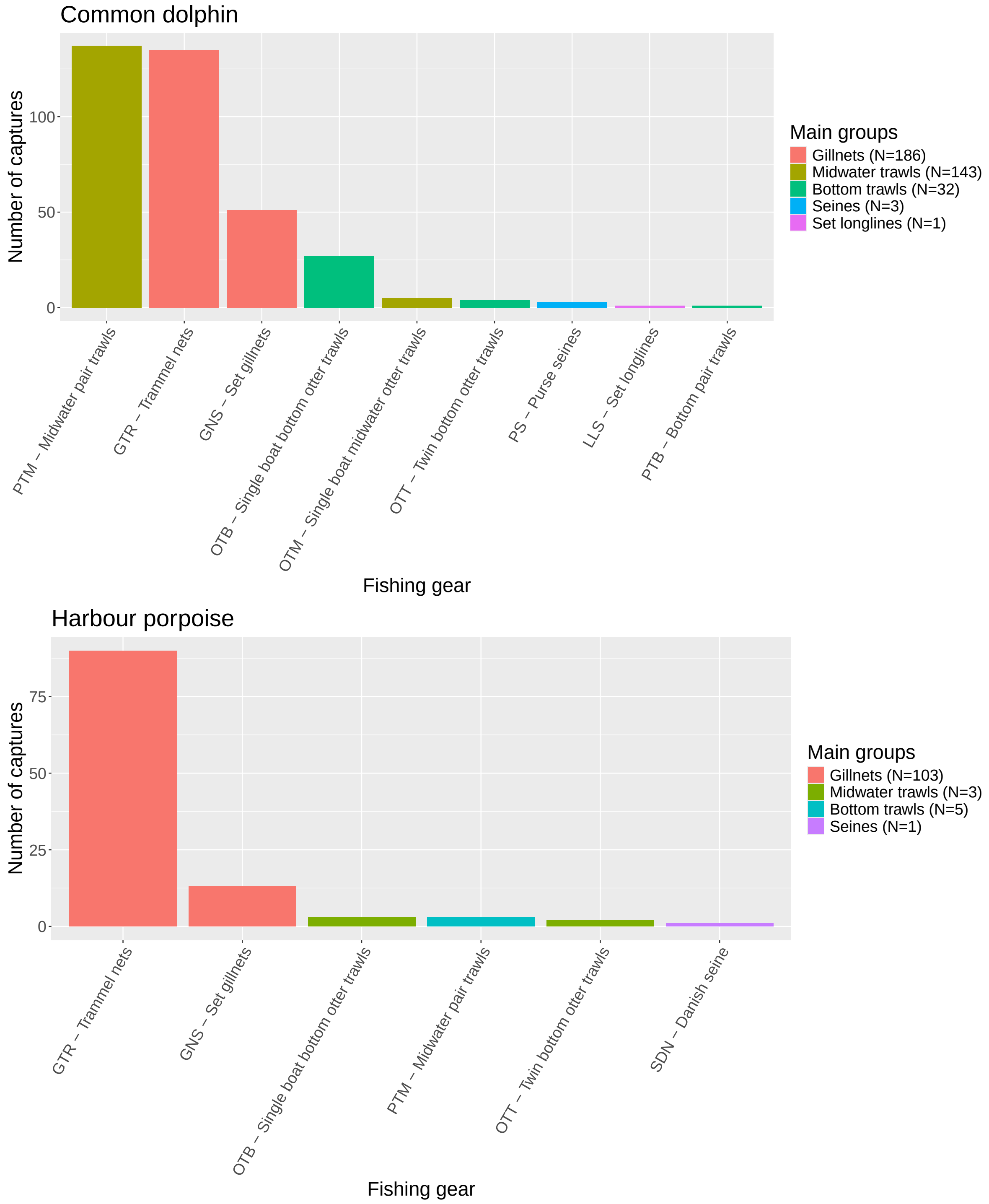

Figure S8: Fishing gear used during reported by-catch events. Barplots displaying the distribution of fishing gears associated with observed/declared by-catch (with phenotypic measurements), respectively for common dolphin and harbour porpoise. Colours indicate the main fishing gear categories, with the number of by-catches per category provided in the legend.

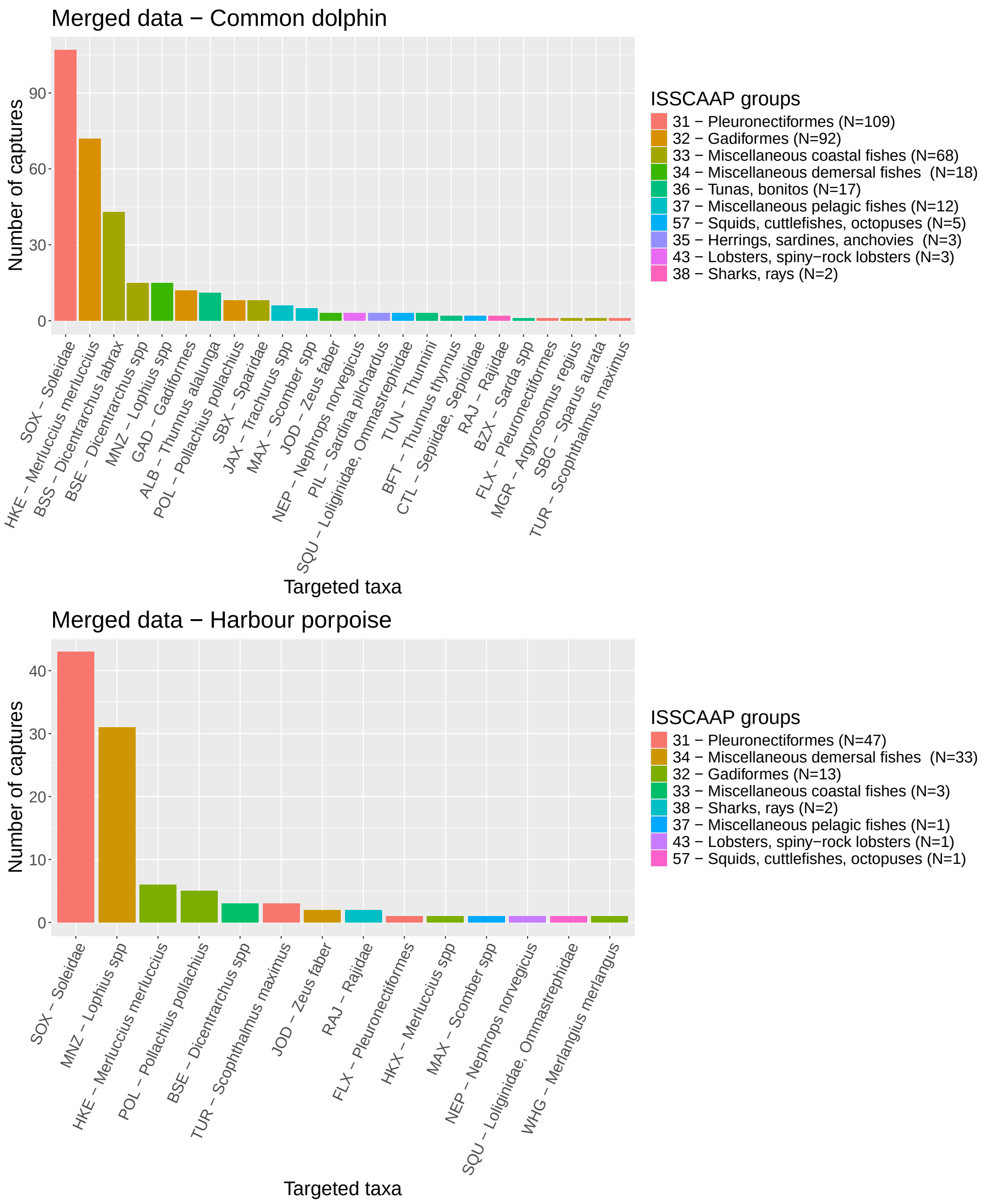

Figure S9: Targeted taxa associated with reported by-catch events (all datasets merged). Barplots displaying the distribution of targeted taxa associated with observed/declared by-catch (with phenotypic measurements), respectively for common dolphin and harbour porpoise. Targeted taxa were declared by fishermen and used as a proxy for main fished taxa when merging observation and declaration data (as direct measurements of main fished taxa were not available in the declaration dataset). It is important to note that this proxy is quite accurate, as there is a 75% overlap between the most fished taxa and the declared targeted taxa at the species level in the observation dataset. Colours indicate associated ISSCAAP groups (International Standard Statistical Classification of Aquatic Animals and Plants, from FAO) used for subsequent analyses, with the number of by-catches per category provided in the legend.

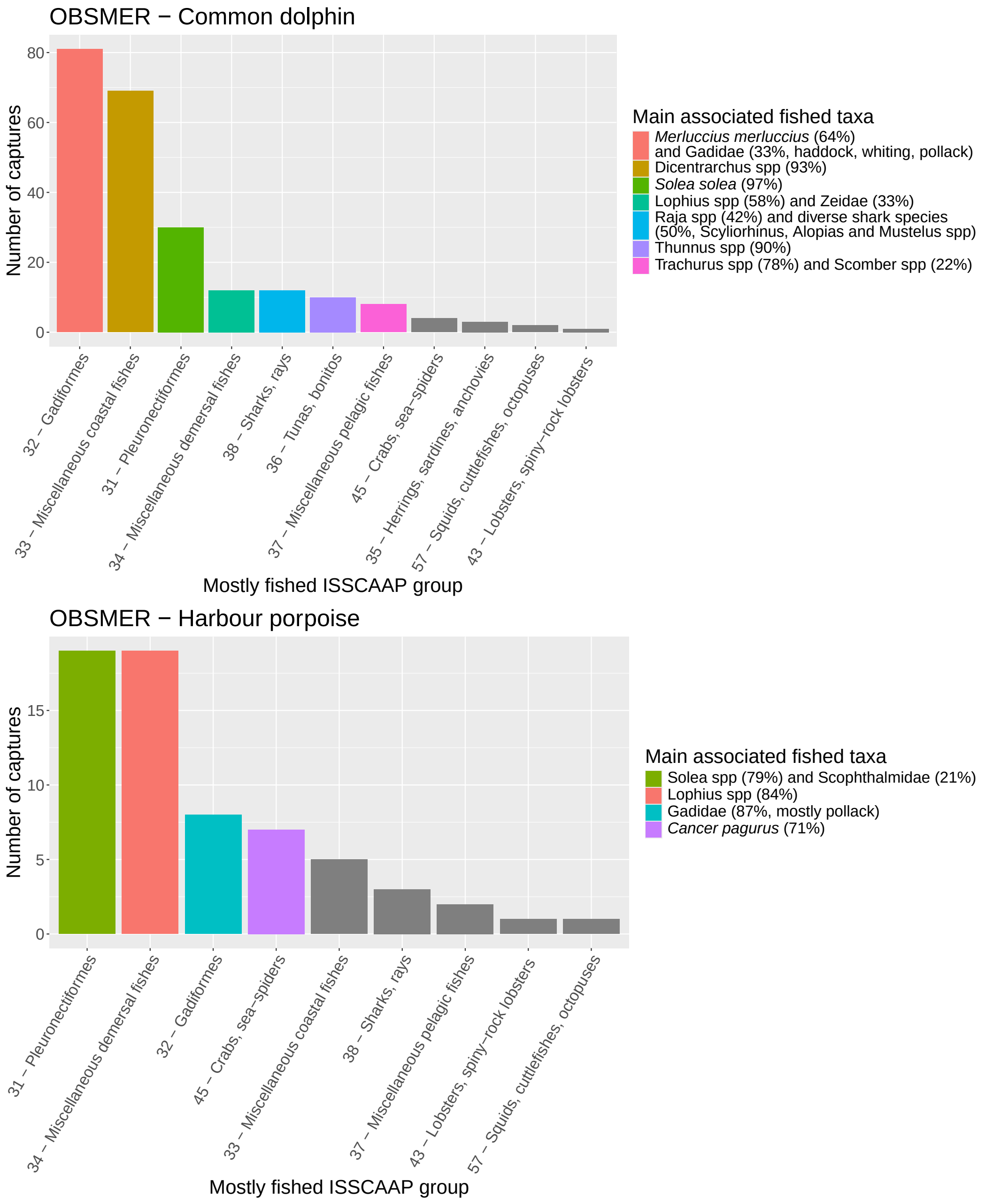

Figure S10: Fished ISSCAAP classes associated with reported by-catch events (observation data only). Barplots displaying the distribution of the most fished ISSCAAP groups (in terms of weight) associated with by-catch events (with phenotypic measurements). This information is only available in the observation dataset (OBSMER), which includes the weight of fished taxa measured by observers. Two distinct graphs are provided here for each of the two species studied. Colours indicate which fished taxa (most fished species or group of species) are mainly associated with the most fished ISSCAAP groups. Fished ISSCAAP groups rarely associated with by-caught individuals are coloured in grey (N<6) and were excluded from subsequent analyses. The main species composition is not detailed for these rare ISSCAAP groups.

ISSCAAP: International Standard Statistical Classification of Aquatic Animals and Plants (from FAO)

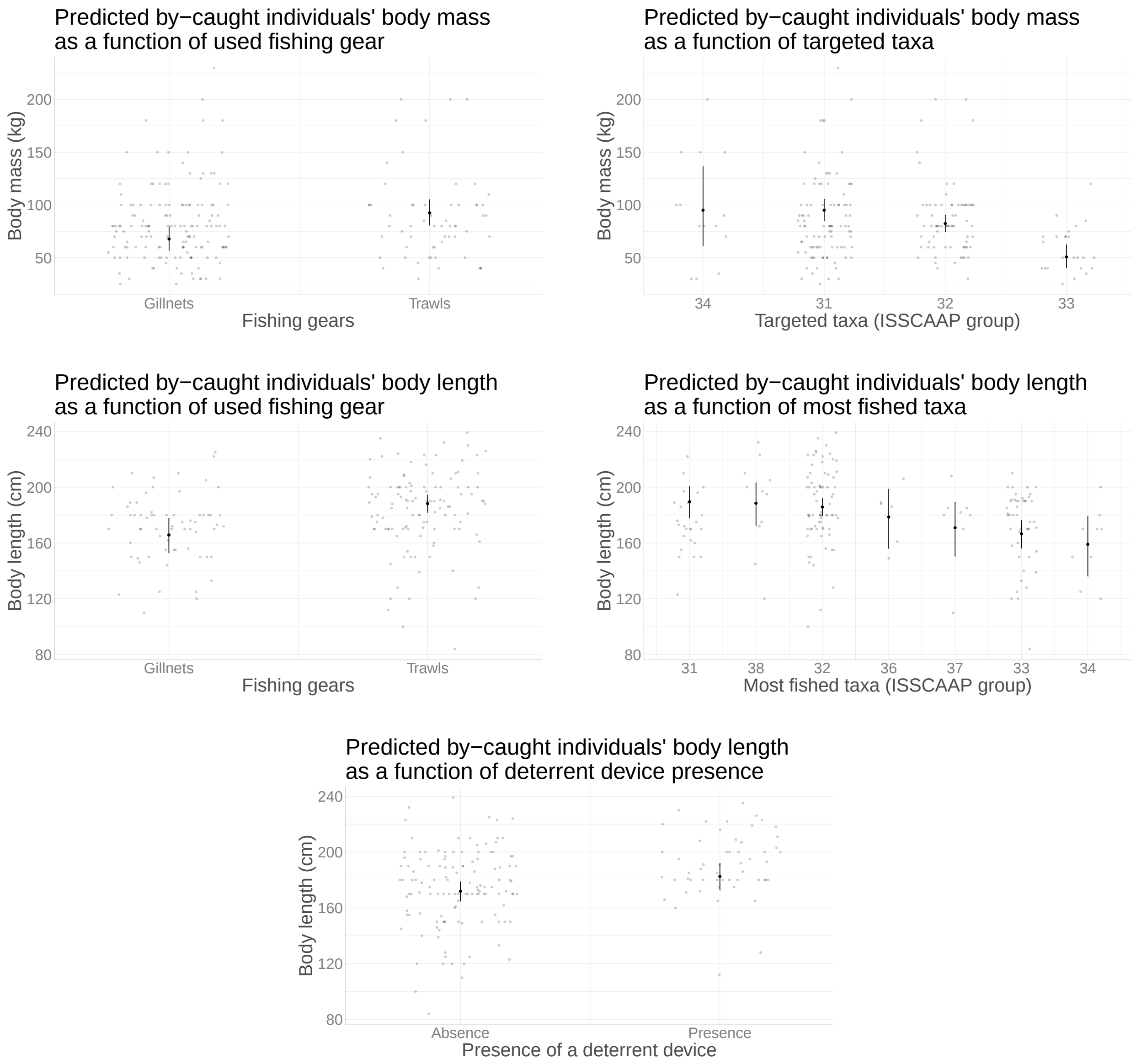

Figure S11: Effects of fishing gear, fished/targeted taxa and deterrent devices on body mass and length of by-caught common dolphin (after transformation of the response variable). The figures show the expected marginal effects of fishing gear and fished/targeted taxa on the BM or BL of by-caught common dolphins, after transforming these variables to avoid deviations from the model's statistical assumptions (see Methods for details). Models have square root and squared transformed response respectively. Predictions have been transformed back to the original response scale in the plots. Standard errors are still on the transformed scale. We displayed the original data points in grey and slightly jittered them (small amount of random variation in the location of the data points, to avoid overplotting) to improve readability. Results were obtained using the `ggemmeans` function of the `ggeffects` `R` package (Lüdecke, 2018). Dark dots indicate the predicted value of body mass or body length as a function of fishing gear or fished/targeted taxa. Error bars indicate the confidence intervals based on standard errors, assuming a normal distribution. The ISSCAAP code descriptions and main associated taxa (see Figures S9 and S10) are provided below.

ISSCAAP: International Standard Statistical Classification of Aquatic Animals and Plants (from FAO), 31 - Pleuronectiformes (mainly soles in our case) / 32 - Gadiformes (mainly hake in our case) / 33 - Miscellaneous coastal fishes (mainly sea bass in our case) / 34 - Miscellaneous demersal fishes (mainly monkfishes in our case) / 36 - Tunas, bonitos / 37 - Miscellaneous pelagic fishes (mainly mackerells in our case) / 38 - Sharks, rays

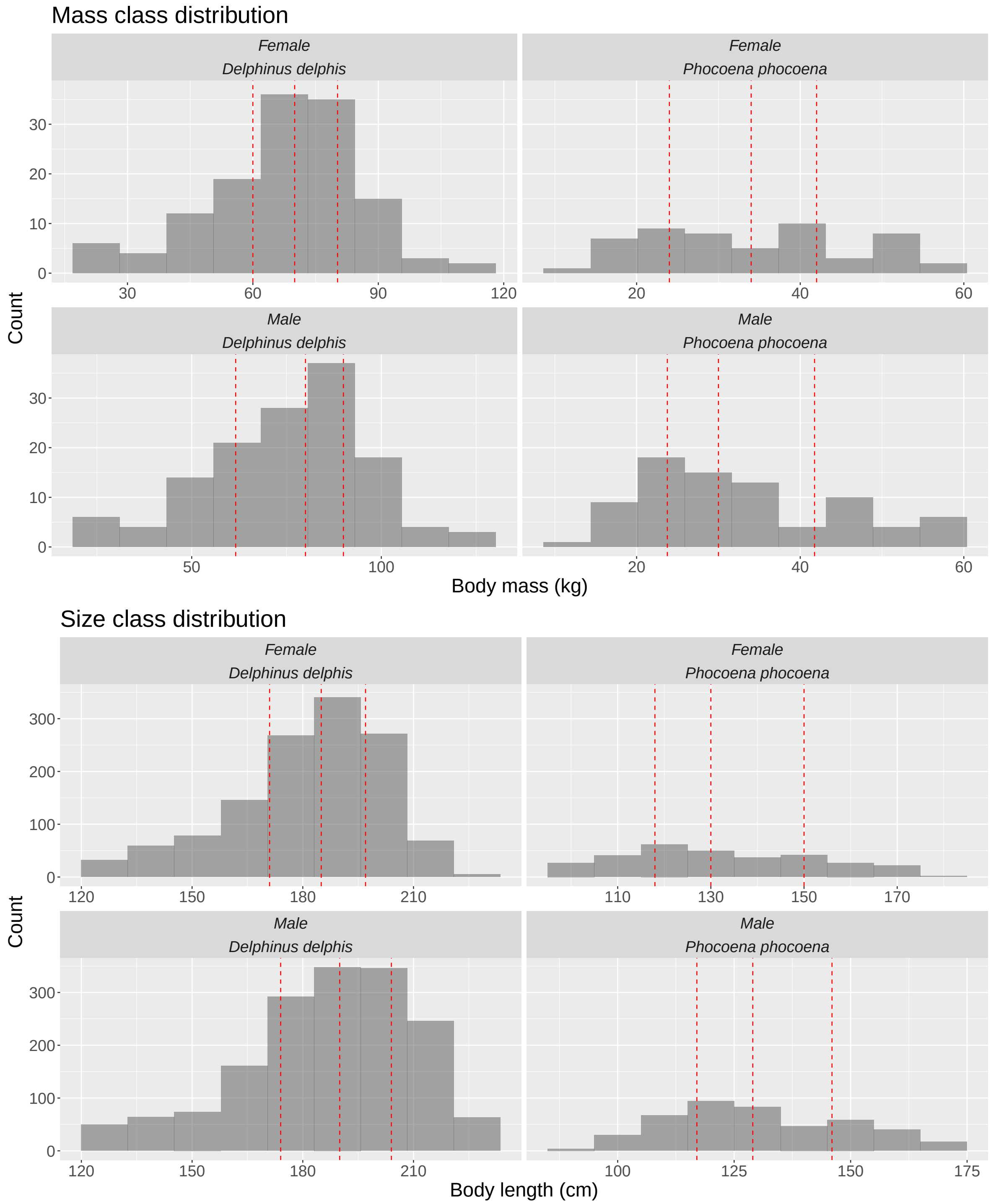

Figure S12: Body length and mass class distribution of stranded individuals (per sex and species). Barplots displaying the distribution of stranded individuals across body length and mass classes, respectively for common dolphin (left graphs) and harbour porpoise (right graphs), males (bottom graphs) and females (top graphs). For each combination of sex and species, we considered nine equal-size classes, ranging from the 2.5th quantile to the 97.5th quantile of the distribution for each species (we discarded the distribution edges to avoid including outliers when comparing distributions). We indicated the quartiles of these truncated distributions by the dashed red horizontal lines.

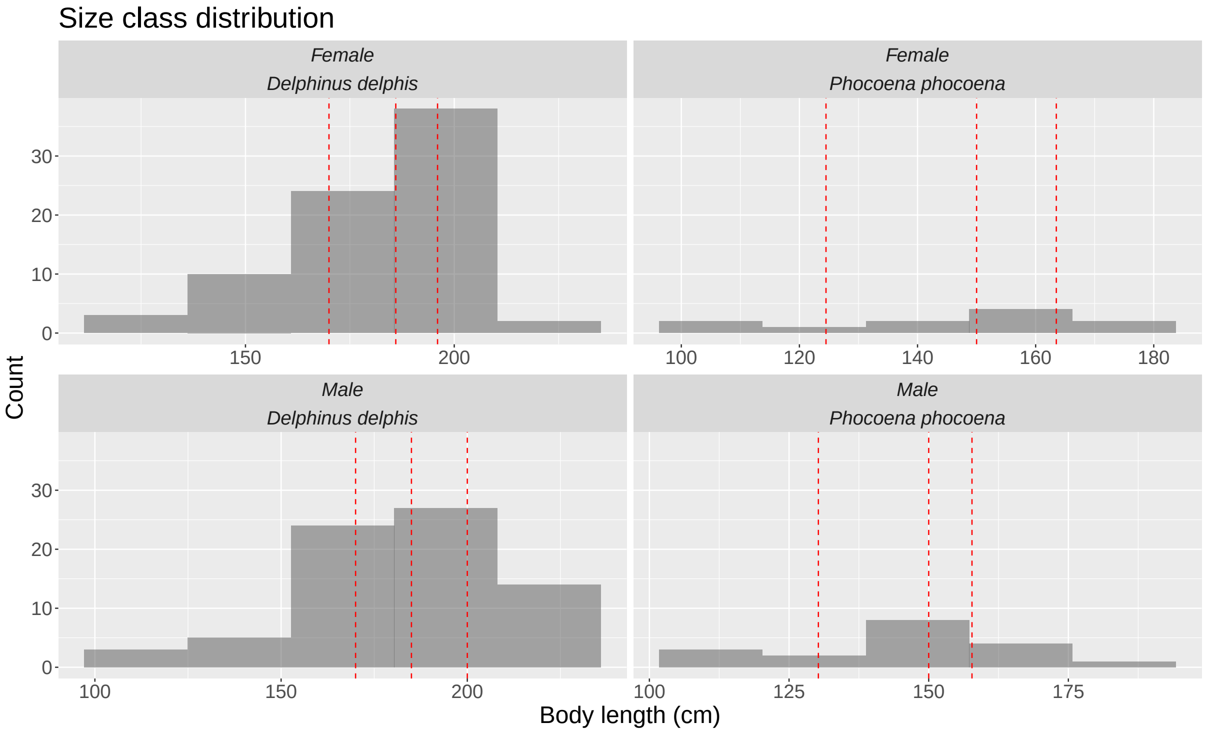

Figure S13: Body length class distribution in observed/declared by-catch (per sex and species). Barplots displaying the distribution of stranded individuals across body length classes, respectively for common dolphin (left graphs) and harbour porpoise (right graphs), males (bottom graphs) and females (top graphs). For each combination of sex and species, we considered nine equal-size classes ranging from the 2.5th quantile to the 97.5th quantile of the distribution for each species (we discarded the distribution edges to avoid including outliers when comparing distributions). We indicated the quartiles of these truncated distributions by the dashed red horizontal lines. We did not provide the body mass class distribution per sex as declarations did not include the sex of by-caught individuals.

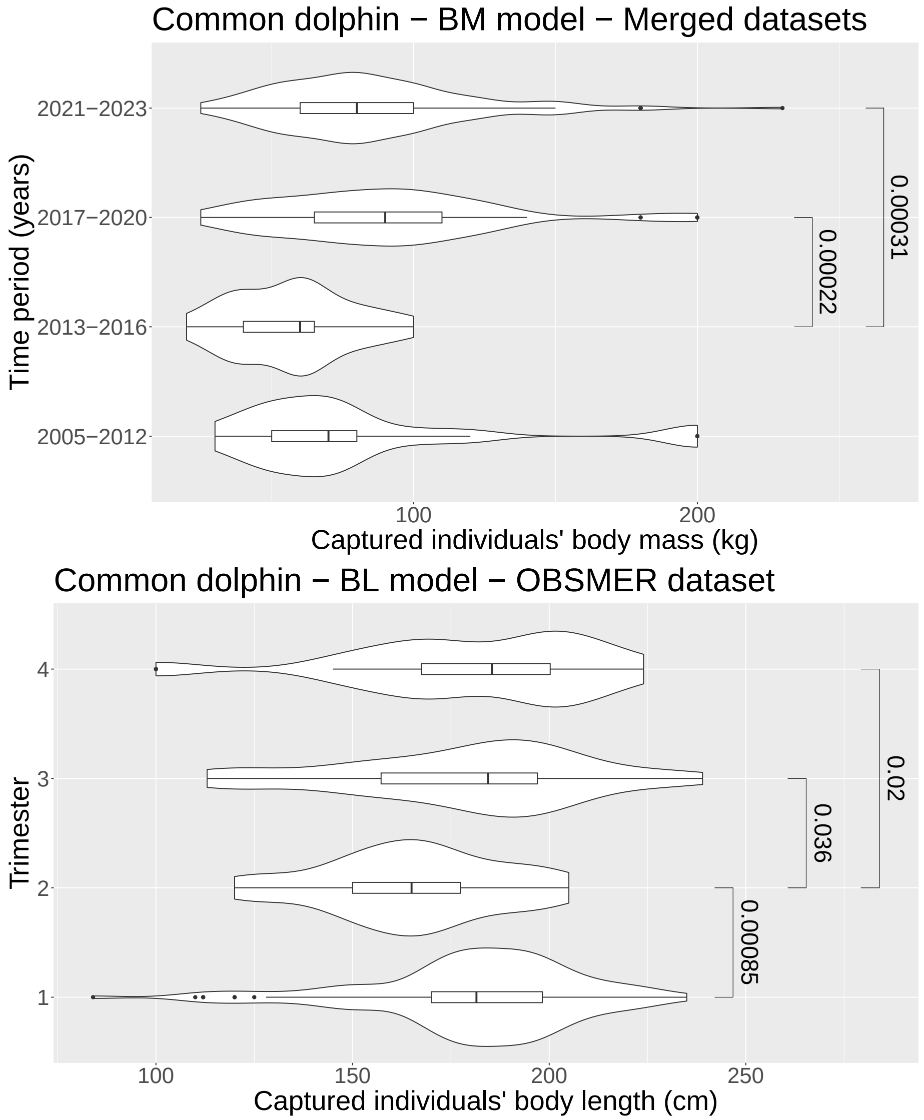

Figure S14: A priori effects of time period and trimester on by-caught individuals' body length (BL) and body mass (BM). These boxplots and violin plots compare the BL distribution of by-caught common dolphins between time periods (range of years) and trimesters, considering either both observed/declared data (merged datasets) or observation (OBSMER) data only (indicated in graph headings). Only cases where we found a significant effect on body length or body mass are shown (Kruskal-Wallis test, $p<0.05$). We then performed pairwise post-hoc tests (Wilcoxon tests) to compare categories of variables. Brackets to the right of the graphs indicate significant differences between categories, with p-values provided above. No correction for test repetition was applied.

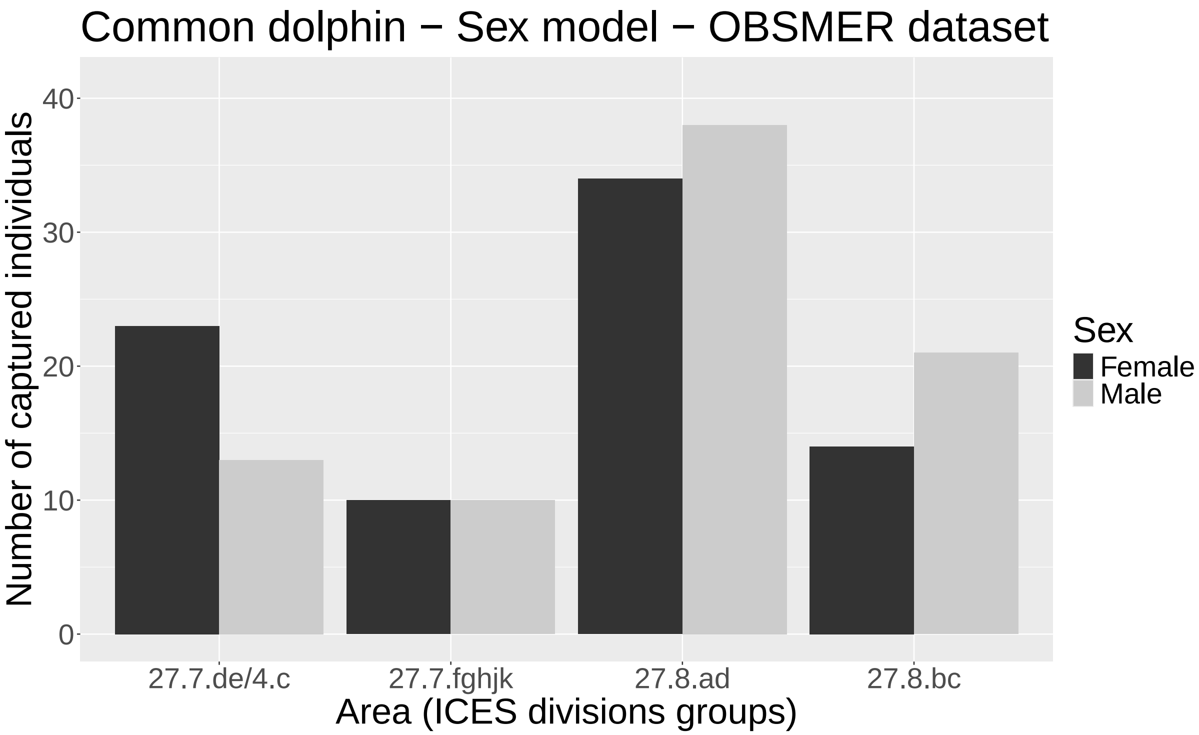

Figure S15: Sex distribution of by-catch as a function of ICES divisions. These barplots compare the sex distribution of by-caught common dolphins between spatial areas (ICES divisions) considering observation (OBSMER) data only.

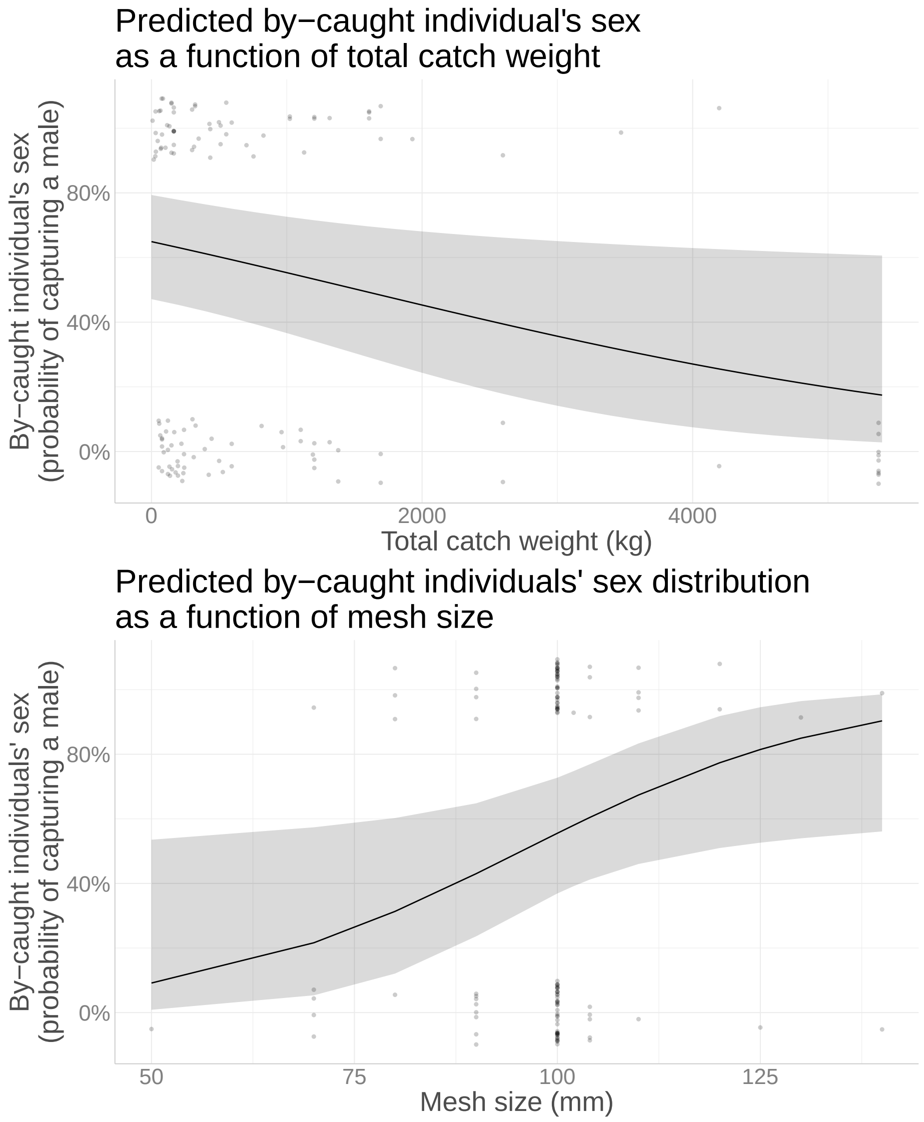

Figure S16: Effects of the total catch weight and mesh size on the sex distribution of by-caught common dolphin. These figures show the predicted marginal effects of total catch weight (during fishing operations associated with by-catches) and fishing gears mesh size on the distribution of by-caught common dolphins' sex. Results were obtained using the `ggemmeans` function of the `ggeffects` `R` package (Lüdecke, 2018). We displayed the original data points in grey and slightly jittered them (adding a small amount of random variation to the location of the data points, to avoid overplotting) to improve readability. The predicted value of captured individuals' sex-ratio (proportion of males) is provided by dark lines, as a function of total catch weight or mesh size, respectively. Grey areas indicate confidence intervals based on standard errors, assuming a normal distribution.

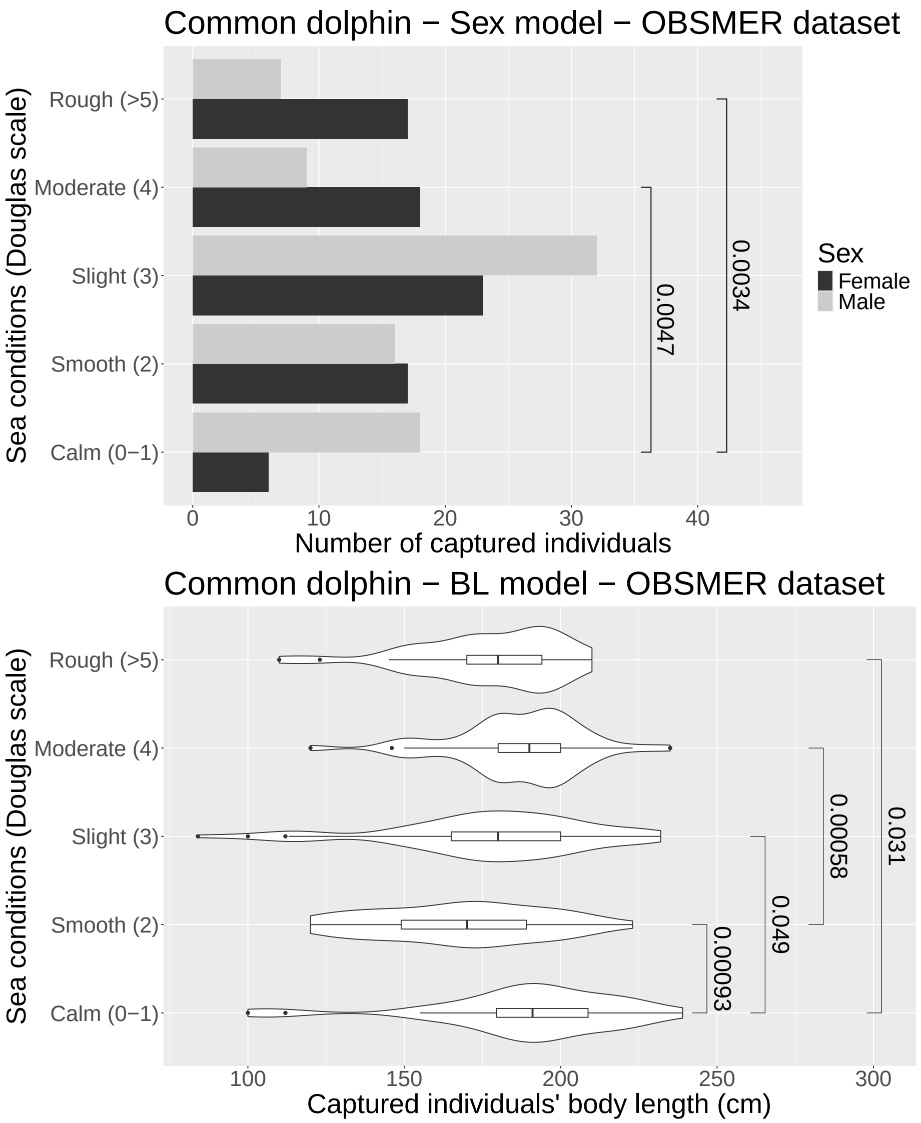

Figure S17: A priori effects of sea state on by-caught individuals' sex and body length (BL). These barplots (for sex) and combined boxplots and violin plots (for BL) compare the sex/BL distribution of by-caught common dolphins between sea states (Douglas scale), considering observation (OBSMER) data only. Only cases where we found a significant effect on sex or BL are displayed here (Fisher tests or Kruskal-Wallis, $p<0.05$). We then conducted pairwise post-hoc tests using respectively Fisher (for sex) or Wilcoxon (for BL) tests to compare variables' categories. Brackets in graphs indicate significant differences between categories, with p-values provided above. No correction for test repetition was applied.
